# An Intruder-Free Fock Space Coupled-Cluster Study of the Potential Energy Curves of LiMg^+^ within the (2,0) Sector

**DOI:** 10.3390/molecules29102364

**Published:** 2024-05-17

**Authors:** Grzegorz Skrzyński, Monika Musial

**Affiliations:** Institute of Chemistry, University of Silesia in Katowice, Szkolna 9, 40-006 Katowice, Poland

**Keywords:** Fock space multireference coupled-cluster method, intermediate Hamiltonian, potential energy curves, spectroscopic constants, LiMg^+^ molecular cation

## Abstract

The potential energy curves (PECs) and spectroscopic constants of the ground and excited states of a LiMg^+^ molecular cation were investigated. We obtained accurate results for the fifteen lowest-lying states of the LiMg^+^ cation using the Intermediate Hamiltonian Fock Space Multireference Coupled Cluster (IH-FS-CC) method applied to the (2,0) sector. Relativistic corrections were accounted for using the third-order Douglas–Kroll method. In each instance, smooth PECs were successfully computed across the entire range of interatomic distances from equilibrium to the dissociation limit. The results are in good accordance with previous studies of this molecular cation. Notably, this study marks the first application of IH-FS-CC in investigating a mixed alkali and alkaline earth molecular cation, proving its usability in determining accurate PECs of such diatomics and their spectroscopic constants.

## 1. Introduction

In recent decades, researchers have shown interest in alkali and alkaline earth molecular dimers given their applications in studies conducted at ultralow temperatures, such as the controlled preparation of many-body entangled states [1] or the measurement of proton-to-electron mass ratio [2]. Also, their respective molecular cations recently attracted the interest of experimental scientists in the context of ultracold studies. Various studies have demonstrated the usefulness of these systems: the KCa^+^ cation was applied in the quantum simulation of solid-state physics [3], NaCa^+^ cations can be used in quantum information processing as quantum logic gates, and RbBa^+^ can be used to study strong-coupling polaronic effects [4,5]. Furthermore, previous findings show that ion-atom sympathetic cooling in a magneto-optical trap is possible for NaCa^+^ cations [6]. The LiCa^+^ cation can be applied as a high-spatial-resolution probe in ultracold chemical reactions [7], and the LiMg^+^ cation can be used to measure the electron dipole moment [8]. Moreover, RbCa^+^ and RbSr^+^ cations can both be applied in studies of collisional quantum features such as scattering resonances or intermolecular effects [9,10].

A highly precise knowledge of potential energy curves (PECs) is essential for the evaluation of the properties of a system and for understanding collision and dissociation processes. Moreover, theoretically computed PECs provide necessary information for experimentalists, aiding them in adjusting their equipment to obtain ultracold species [11]. However, calculating PECs using standard computational schemes can be challenging, even with relatively small systems like diatomic molecular cations. Difficulty arises when treating the homolytic dissociation of a single bond in which open-shell fragments are produced. Therefore, using the restricted Hartree–Fock scheme (RHF) for distances far from equilibrium is incorrect. Alternatively, unrestricted Hartree–Fock (UHF) or restricted open-shell Hartree–Fock (ROHF) methods are required, which are known for their convergence problems with the HF and post-HF equations. Theoretical chemistry has proposed several solutions to address this issue. A frequently employed method in studies of excited states (EEs) is the Equation-of-Motion Coupled-Cluster (EE-EOM-CC) method [12,13,14,15,16,17]. Unfortunately, this method is not size-extensive, which is the reason for its limited use in studies of PECs [18,19]. A more commonly used approach is the multireference configuration interaction (MRCI) method, which is size-extensive only in its full CI (FCI) variant, making it impossible to carry out in a reasonable time period for systems with large numbers of electrons. As a workaround, researchers often opt to perform calculations exclusively for valence electrons while freezing the core, impacting the accuracy of the results. To overcome this limitation, some studies use pseudopotential or ECP (Effective Core Potential) approximations [20,21,22]. In this approach, electron correlation is accounted for only for valence electrons, and it is usually reduced to the CISD (S–singles; D–doubles) method, which is to the FCI for the two-electron case. This approach also requires considering some additional parameters representing the potential of core electrons, and they cannot be used universally for any chosen system.

In this study, we focus on computing the accurate potential energy curves and spectroscopic constants of a molecular cation, LiMg^+^. We decided to utilize an alternative approach using the IH-FS-CCSD method (the Intermediate Hamiltonian Fock Space Multireference CC method with singles and doubles) [23], obtaining results in the (2,0) sector, which is explained further in the Methods section of this manuscript. This method allows one to choose a doubly ionized system as a reference, dissociating it into closed-shell fragments. This makes it possible to use the RHF as a reference function for any internuclear distance. Then, calculations using the double-electron attachment (DEA) formalism are performed to produce energies of the ground and excited states of the studied system. In the case of the system studied here in the form of the LiMg^+^ molecular cation, the reference function was determined for the triple-positive ion LiMg^3+^:

(1) $$LiMg^{3+}\to Li^{+}+Mg^{2+},$$

then, utilizing the DEA formalism:

(2) $$LiMg^{3+}\overset{DEA}{\to }LiMg^{+},$$

we obtain the energies of the ground and excited states of the LiMg^+^. This approach allows smooth PECs to be obtained for the whole range of internuclear distances. IH-FS-CCSD(2,0) is also a strictly size-extensive method—this property ensures that the energies of the electronic states of the system converge at an infinite distance to the sum of its atomic values.

The IH-FS-CCSD(2,0) method [23] was previously proven successful in obtaining PECs and the spectroscopic constants of diatomic molecules composed of alkali metals in several studies, as demonstrated in recent papers involving NaLi [24] or LiRb [25] molecules. However, this marks the inaugural application of this computational approach to study a molecular cation composed of alkali and alkaline earth metals, specifically the LiMg^+^ molecular cation. Our objective was to define a suitable methodology for the calculation of such species and present benchmark accuracy results.

The next section of this work presents computational details and the results obtained, along with a discussion. The Methods section describes the theoretical background of the IH-FS-CCSD(2,0) method, and the final section offers conclusion, new insights, and future perspectives.

## 2. Results and Discussion

### 2.1. Background

The LiMg^+^ system was previously studied with respect to its spectroscopic properties, both theoretically and experimentally, in only a few papers. The oldest theoretical works concerned only the spectroscopic constants of the ground state of the molecular cation [26,27,28]. More recent papers published in the last decade employed various methods to study the spectroscopic constants of the ground and excited states of LiMg^+^, including the MRCI calculations of Gao and Gao [29], the pseudopotential study of ElOualhazi and Berriche [8], the calculations of Fedorov et al. using CC and MRCI methods [30], the EE-EOM-CC study of Bala et al. [18], and the CC calculations of Śmiałkowski and Tomza [11]. In terms of experimental studies, there is a single paper by Persinger et al. in which the spectroscopic values of only the ground state of LiMg^+^ are given since the paper was focused on the neutral LiMg molecule [31].

### 2.2. Computational Details

Calculations were carried out using the IH-FS-CCSD(2,0) method [23], which is explained in detail in the Methods section of this manuscript. The main results were computed using the uncontracted ANO-RCC [32] basis set with additional diffuse functions, which we called unANO-RCC+. For each atom, we have added six diffuse functions which are presented in Table 1. These exponents were determined through the even-tempered scheme [33]—the ratio between the subsequent exponents is equal to 0.35 for lithium and 0.4 for magnesium. This ensures the accurate ordering of atomic states. The final dimension of the unANO-RCC+ basis set is 242 spherical harmonic basis functions.

The LiMg^3+^ cation was chosen as the reference system for all double-electron attachment calculations, and all electrons were correlated. The reference function employed throughout this study was always the restricted Hartree–Fock function. To assess the impact of relativistic effects on the spectroscopic constants of the LiMg^+^ cation, we adopted a two-step approach. Initially, the results for the LiMg^+^ cation were computed using the uncontracted ANO-RCC basis set without the additional diffuse functions that are presented above—we called this basis unANO-RCC. Afterward, scalar relativistic corrections were applied using the third order Douglass–Kroll [34] method, and the calculations were repeated using the standard ANO-RCC basis set. The differences between obtained relativistic and non-relativistic values were computed and added to the spectroscopic constants derived from the unANO-RCC+ basis set.

All calculations were carried out using our own local modules for the IH-FS-CCSD(2,0) calculations [23,24], implemented in the GAMESS [35] ver. 2021 R2 Patch 1 and ACES II [36] ver. 2.7.0 software packages. The spectroscopic constants were determined using Robert J. LeRoy’s LEVEL program ver. 8.0 [37]. The active space size for the IH-FS-CCSD(2,0)/unANO-RCC+ calculations was set to 70 (i.e., the 70 lowest virtual orbitals were chosen as active), resulting in a model space size of 4900. Similarly, for both the IH-FS-CCSD(2,0)/unANO-RCC and IH-FS-CCSD(2,0) DK3/ANO-RCC calculations, the active space size was set to 54, and the model space size was equal to 2916.

### 2.3. Atomic Energies at the Dissociation Limit

Size extensivity is a crucial property in PEC calculations—the energies of the electronic states of a system must converge to the sum of their atomic values at an infinite distance. IH-FS-CCSD(2,0) is a strictly size-extensive method. It is presented in Table 2, which displays values computed using the three basis sets that were utilized in this work. The first column depicts a given dissociation limit, while the next columns depict energy values for Li or Li^+^ and Mg or Mg^+^, respectively. The second-to-last column displays the sum of these energies, while the last column presents the energy at the corresponding dissociation limit obtained using the IH-FS-CCSD(2,0) method. The energies of the Li^+^ cation were computed using the CCSD method (no electrons attached) for the Li atom; for the Mg^+^ cation, we used IH-FS-CCSD(1,0) equivalent to EA-EOM-CCSD (one electron attached); and for the Mg atom, the IH-FS-CCSD(2,0) method was utilized (two electrons attached). The results are equal, proving the size extensivity of the method.

We also compared the obtained excitation energies of the lithium and magnesium atoms with available experimental data [38], as shown in Table 3. In the columns, we depict the four lowest excitation states of Li and the same for Mg; in the rows, there are values for the three basis sets used in this work. The agreement between the experimental values is dependent on the basis set. For lithium, the ANO-RCC basis set performs accurately only for the first two excited states, altering the order of the third and fourth. The unANO-RCC+ and unANO-RCC basis sets are more accurate, with differences ranging from less than 0.001 eV to 0.003 eV and from 0.001 eV to 0.084 eV, respectively. For magnesium, ANO-RCC is more accurate, with discrepancies ranging from 0.011 eV to 0.042 eV. The differences for unANO-RCC+ and unANO-RCC are between 0.015 eV and 0.025 eV and between 0.002 eV and 0.028 eV, respectively. Importantly, only the first two terms of lithium are a part of the dissociation limit presented in this work; therefore, we were able to use the ANO-RCC basis set. In summary, our method closely reproduces the experimental excitation energies.

### 2.4. Potential Energy Curves

**Figure 1:**
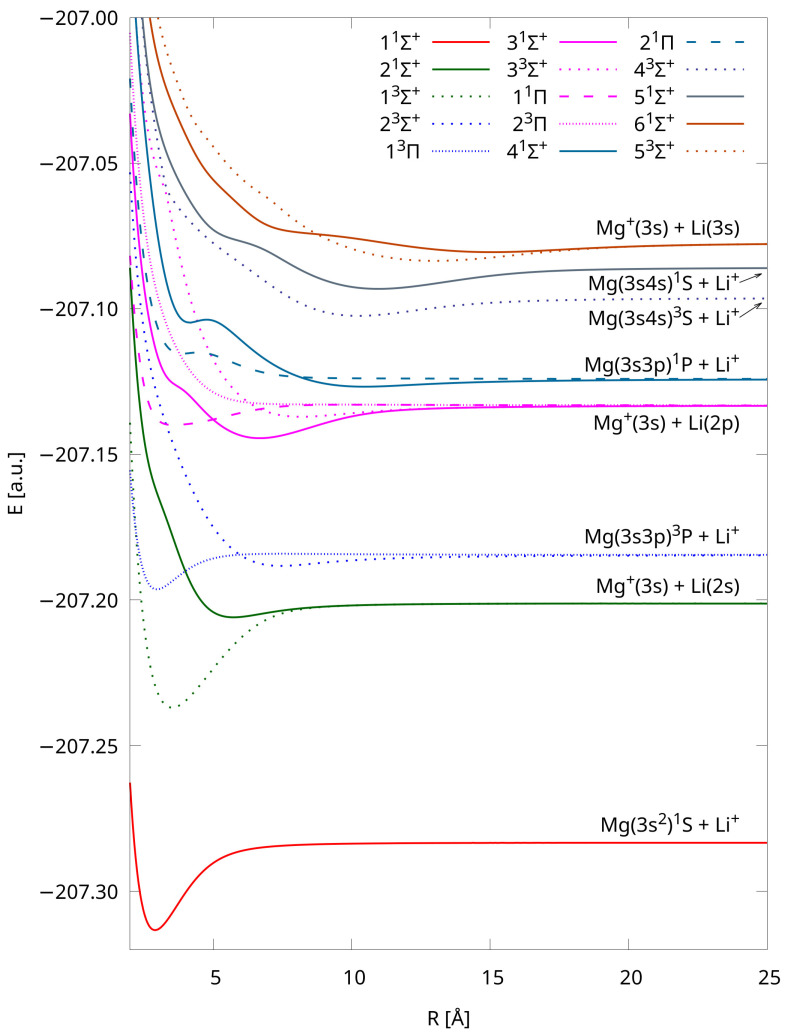
Potential energy curves of LiMg^+^ calculated using the IH-FS-CCSD(2,0)/unANO-RCC+ method for the eight lowest dissociation limits.

The potential energy curves of the fifteen lowest-lying electronic states of LiMg^+^ were calculated using the IH-FS-CCSD(2,0) method and the unANO-RCC+ basis set, and they are presented in Figure 1. The curves are distinguished by eight different colors corresponding to eight dissociation limits and four different point types depending on the multiplicity and symmetry of their states. The interatomic distances presented in Figure 1 are limited to 25 Å, but the tabulated total energies of each state up to 200 Å are provided in the Supplementary Materials.

**Figure 2:**
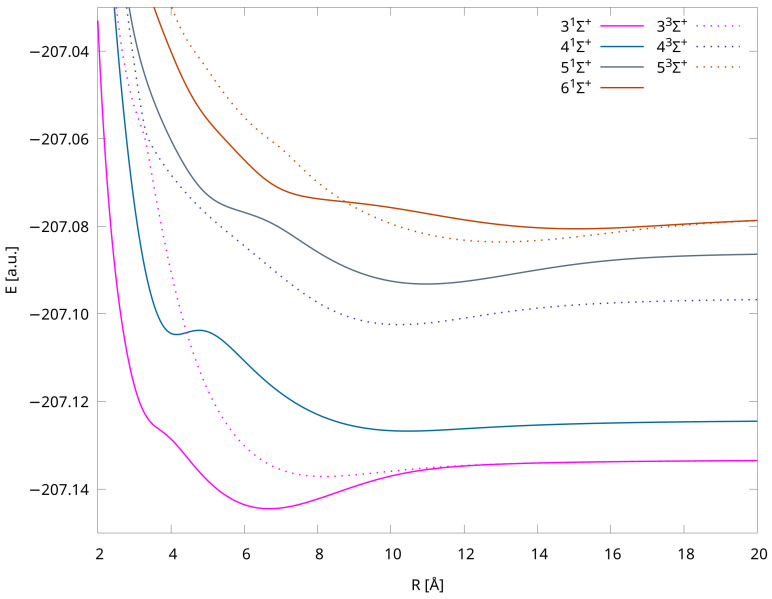
Potential energy curves of LiMg^+^ cation for states in which avoided crossing phenomenon occurs. Obtained using IH-FS-CCSD(2,0)/unANO-RCC+ method.

Among the fifteen PECs presented in this work, the $4^{1}\Sigma^{+}$ state is the only one characterized as having two minima. As expected, the shapes of higher-lying curves are distorted, with noticeable undulations, and they do not represent regular Morse-like potentials due to the phenomenon of avoided crossing between adjacent adiabatic potential energy curves of the same symmetry and multiplicity. Its presence can be explained by the occurrence of the interaction and charge transfer process between the electronic states of Li-Mg^+^ and Li^+^-Mg. To better illustrate these interactions, we present specific PECs in Figure 2. The exact positions of the avoided crossing interactions are shown in Table 4, and they are compared with positions presented in [8]. The agreement is very good, with differences of less than 2%.

**Figure 3:**
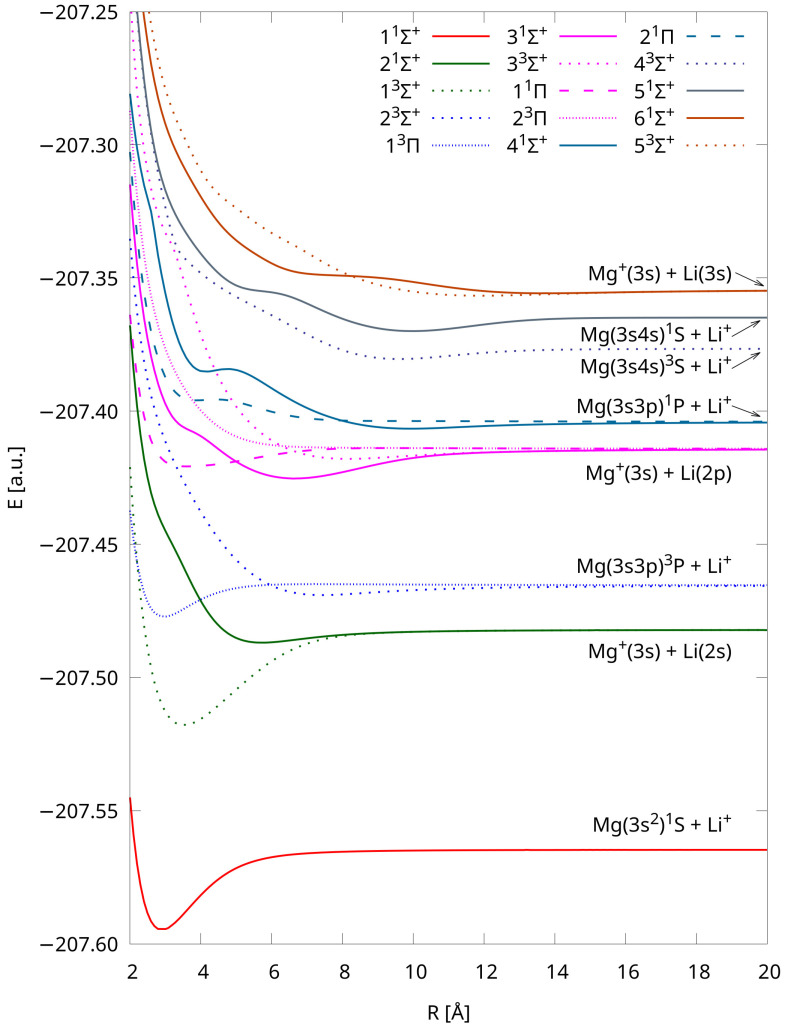
Potential energy curves of LiMg^+^ calculated using the IH-FS-CCSD(2,0) DK3/ANO-RCC method for the eight lowest dissociation limits.

We also depicted PECs obtained using the IH-FS-CCSD(2,0) DK3/ANO-RCC method in Figure 3. The tabulated total energies of each state up to 300 Å are also provided in the Supplementary Material. These PECs are shown purely for comparison as they were used only while evaluating the influence of relativistic effects on the spectroscopic constants. Nonetheless, their shapes closely resemble those obtained using the unANO-RCC+ method (cf. Figure 1). Furthermore, the PECs presented in Figure 1 and Figure 3 uniformly dissociate into the same limits, and their shapes align with those available in the literature [8].

### 2.5. Spectroscopic Constants

The following spectroscopic constants of the LiMg^+^ molecular cation were obtained: equilibrium distances $R_{e}$, well depths $D_{e}$, adiabatic excitation energies $T_{e}$, harmonic frequencies $\omega_{e}$, anharmonicity constants $\omega_{e}$$X_{e}$, and equilibrium rotational constants $B_{e}$. The results obtained using the IH-FS-CCSD(2,0)/unANO-RCC+ method are shown in Table 5. The relativistic contributions for each constant are given in parentheses. The magnitude of the impact of relativistic effects on the spectroscopic constants of the LiMg^+^ cation varies for different states, but we observe that the contribution is smaller for lower-lying excited states than for higher-energy states. These relativistic contributions are smaller than for, e.g., LiRb [25], but they are still non-negligible, and they have some impact on the quality of the results.

For comparison purposes, we cited previous results from a number of different papers for LiMg^+^ which were obtained using different methods. These include CCSD(T)/aug-cc-pCVQZ [11], (CCSD(T) ≡ CCSD + perturbative triples [39]), CCSDT/cc-pCVQZ (CCSDT ≡ CCSD + full triples [40]), MRCI/cc-pCVQZ [30], and CCSD(T)/cc-pVQZ [18] calculations for the ground state; EE-EOM-CCSD/cc-pVQZ [18] calculations for excited states; MRCI/aug-cc-pV5Z [29] and an ab initio approach based on non-empirical pseudopotentials [8] for both the ground and excited states; and, as far as we know, the only experimental values for the ground state of LiMg^+^, $\omega_{e}$ and $\omega_{e}x_{e}$ [31].

Comparing our results for $\omega_{e}$ and $\omega_{e}x_{e}$ with the paper by Persinger et al. [31], we can clearly see that our calculations reproduce the experimental values very well, with absolute differences of less than 1% and ∼7.7% for $\omega_{e}$ and $\omega_{e}x_{e}$, respectively. Moreover, our $\omega_{e}$ value is the closest to the experimental one among all previous theoretical works.

Our results are also in good agreement with theoretical values obtained in previous works for most cases. The exceptions are values calculated for the 2^1^$\Sigma^{+}$ and 3^1^$\Sigma^{+}$ states in a paper by Bala et al. [18], in which they explicitly stated that their $R_{e}$ was largely different from that considered in [8], which makes it impossible to compare the values directly.

For the ground state, our $R_{e}$ value is the same as the CCSDT value obtained by Fedorov et al. [30]. Also, our $D_{e}$ value is the closest to the result [11] calculated using the so-called “gold standard” CCSD(T) method [39]. The IH-FS-CCSD(2,0) method is capable of providing very accurate results even if it does not consider the effect of triple excitations.

The comparison with the paper by ElOualhazi and Berriche [8] is the most extensive as it is the only work comprising values for all of the excited states of LiMg^+^ studied here. The closest $R_{e}$ values are observed for the 1^3^$\Sigma^{+}$ and 3^1^$\Sigma^{+}$ states, for which the absolute difference is 0.002 Å. The most significant error—less than 8%—is calculated for the 6^1^$\Sigma^{+}$ state. The good alignment of $R_{e}$ indicates a similar agreement in the derived $B_{e}$ values.

Also, we identified one repulsive state, i.e., the $2^{3}\Pi$ state. The cited paper defines this state as having an extremely shallow $D_{e}$ of just 2 cm^−1^; thus, we believe it is probably a plateau. Moreover, we determined two states—4^1^$\Sigma^{+}$ and 2^1^Π—to have barriers, both of which retain the same dissociation limit. The heights of these barriers are 212 cm^−1^ and 91 cm^−1^, respectively. These values correlate to the $D_{e}$ values reported in the cited paper. For the other states, our $D_{e}$ is closest in the case of the 1^1^Π state (an absolute difference of 20 cm^−1^) and farthest in the case of the 5^3^$\Sigma^{+}$ state.

The $T_{e}$ values show the best agreement throughout all states among the considered spectroscopic constants; the largest difference is less than 3% for the 5^3^$\Sigma^{+}$ state, and for most states, it is less than 1%. In nearly all cases, our $\omega_{e}$ values are similar to the cited ones. The exceptions are the 1^1^Π state and the inner minimum of the 4^1^$\Sigma^{+}$ state, for which the absolute differences are larger than 10 cm^−1^. As for $\omega_{e}$$x_{e}$, the agreement is weak in most cases; this is due to the shapes of the PECs in large internuclear distances, which are described well by the IH-FS-CCSD(2,0) method but significantly less well by pseudopotential-based methods.

In summary, the accuracy achieved is reliable in comparison with Ref. [8], though it varies depending upon the specific state and the spectroscopic constant in consideration.

## 3. Methods

Within the single-reference (SR) coupled-cluster theory, the Schrödinger equation

(3) H|Ψ〉=E|Ψ〉

is solved, assuming the exponential form of the wave function |Ψ〉:

(4) $$|\Psi \rangle =e^{T}|\Phi_{0}\rangle$$

where H is a Hamilton operator, E is the total energy of the system, $|\Phi_{0}\rangle$ is a reference (usually Hartree–Fock) function, and T is a coupled-cluster excitation operator. Within the current approximation of singles and doubles, it takes the form

(5) $$T=T_{1}+T_{2}$$

where the operator $T_{1}=\sum_{ia}t_{i}^{a}a^{†}i$ is responsible for single excitation, i.e., moving an electron from an occupied level *i* to an unoccupied level *a* and, analogously, the $T_{2}=\sum_{ijab}t_{ij}^{ab}a^{†}b^{†}ji$ is responsible for double excitation, i.e., moving two electrons from occupied levels *i*,*j* to unoccupied levels *a*,*b*. In situations in which the reference function $|\Phi_{0}\rangle$ is of a multiconfigurational character (which is the case for potential energy curve calculations) it is necessary to generalize the SR approach to the multireference (MR) case, i.e., to replace the Schrödinger equation, Equation (3), with its Bloch equivalent:

(6) $$H_{eff}|\Psi_{0}\rangle =E|\Psi_{0}\rangle$$

The superiority of the multireference approach relies on the fact that the exact energy, *E*, is obtained by solving the above equation or, equivalently, by the diagonalization of the effective Hamiltonian operator $H_{eff}$:

(7) $$H_{eff}=\hat{P}H\Omega \hat{P}$$

within a small subspace (**M**) of the full configurational space (**M^0^**), which is called a model space and is defined by the projection operator $\hat{P}$, note that $|\Psi_{0}\rangle$ is a component of the exact wave function |Ψ〉 residing within the model space, i.e., $|\Psi_{0}\rangle =\hat{P}|\Psi \rangle$. Of course, the construction of the $H_{eff}$ is not trivial, and it requires employing the wave operator Ω, reproducing, by definition, the exact wave function |Ψ〉 by operating on the model function $|\Psi_{0}\rangle$:

(8) $$\left|\Psi \rangle =\Omega \right|\Psi_{0}\rangle$$

The form of the wave operator Ω depends on the assumed variant of the multireference theory. The characteristic feature of the Fock space formulation [41,42] used in the current work is a specific form of the model space which, by definition, can contain a variable number of electrons. Within the scheme adopted here, the valence Fock space is composed of three sectors defined by the following projection operators, respectively:

(9) $$\hat{P}={\hat{P}}^{\left(0,0\right)}+{\hat{P}}^{\left(1,0\right)}+{\hat{P}}^{\left(2,0\right)}$$

The 0-valence sector corresponds to the reference function $|\Phi_{0}\rangle$, and its projection operator is equal to ${\hat{P}}^{\left(0,0\right)}=|\Phi_{0}\rangle \langle \Phi_{0}|$. The projectors for the remaining two sectors, one-valence ${\hat{P}}^{\left(1,0\right)}$ and two-valence ${\hat{P}}^{\left(2,0\right)}$, are written as

(10) $$\begin{array}{ccc} {\hat{P}}^{\left(1,0\right)} & = & \sum_{\alpha }|\Phi^{\alpha }\rangle \langle \Phi^{\alpha }| \end{array}$$

(11) $$\begin{array}{ccc} {\hat{P}}^{\left(2,0\right)} & = & \sum_{\alpha \beta }|\Phi^{\alpha \beta }\rangle \langle \Phi^{\alpha \beta }| \end{array}$$

where $|\Phi^{\alpha }\rangle$ represents the configuration with one additional electron placed on the valence level α, while $|\Phi^{\alpha \beta }\rangle$ represents the configuration with two additional electrons placed on the valence levels α and β.

The wave operator Ω for the FS-CCSD(2,0) approach takes the form

(12) $$\Omega =\left\{e^{{\tilde{\tilde{S}}}^{\left(2,0\right)}}\right\}\hat{P}$$

where the brackets { } indicate the normal order of the creation–annihilation operators. The coupled-cluster excitation operator ${\tilde{\tilde{S}}}^{\left(2,0\right)}$ which defines the wave operator for the (2,0) sector [23], Equation (12), is expressed as

(13) $${\tilde{\tilde{S}}}^{\left(2,0\right)}=S^{\left(0,0\right)}+S^{\left(1,0\right)}+S^{\left(2,0\right)}=S^{\left(0,0\right)}+{\tilde{S}}^{\left(2,0\right)}=T+{\tilde{S}}^{\left(2,0\right)}$$

and, by definition, the $S^{\left(k,0\right)}$ operator contains *k* valence particle annihilation operators. This implies that for l<k,

(14) $$S^{\left(k,0\right)}{\hat{P}}^{\left(l,0\right)}=0$$

The amplitude equations for each sector of the Fock space can be obtained on the basis of the general Bloch equation written as

(15) $$\hat{Q}H\Omega \hat{P}=\hat{Q}\Omega H_{eff}\hat{P}$$

where $\hat{Q}$ is an operator for the orthogonal part ($M_{⊥}$) of the full configuration space expressed as $\hat{Q}=\hat{1}-\hat{P}$. For the 0-valence sector, the latter equation becomes

(16) $${\hat{Q}}^{\left(0,0\right)}He^{S^{\left(0,0\right)}}{\hat{P}}^{\left(0,0\right)}={\hat{Q}}^{\left(0,0\right)}e^{S^{\left(0,0\right)}}H_{eff}{\hat{P}}^{\left(0,0\right)}$$

Note that the last equation is equivalent to the single-reference Schrödinger equation, Equation (3), since in the one-dimensional case, $H_{eff}=E$, $S^{\left(0,0\right)}=T$, and ${\hat{Q}}^{\left(0,0\right)}$ is an orthogonal subspace spanned by the determinants excited with respect to the $|\Phi_{0}\rangle$ reference.

For the (1,0) sector, we obtain

(17) $${\hat{Q}}^{\left(1,0\right)}He^{S^{\left(0,0\right)}+S^{\left(1,0\right)}}{\hat{P}}^{\left(1,0\right)}={\hat{Q}}^{\left(1,0\right)}e^{S^{\left(0,0\right)}+S^{\left(1,0\right)}}H_{eff}{\hat{P}}^{\left(1,0\right)}$$

and analogously, for the (2,0) sector, we have

(18) $${\hat{Q}}^{\left(2,0\right)}He^{S^{\left(0,0\right)}+S^{\left(1,0\right)}+S^{\left(2,0\right)}}{\hat{P}}^{\left(2,0\right)}={\hat{Q}}^{\left(2,0\right)}e^{S^{\left(0,0\right)}+S^{\left(1,0\right)}+S^{\left(2,0\right)}}H_{eff}{\hat{P}}^{\left(2,0\right)}$$

Owing to the killer condition, Equation (14), we eliminate from the exponential in Equation (16) the operators $S^{\left(1,0\right)}$ and $S^{\left(2,0\right)}$, and, similarly, from Equation (17), we eliminate the operator $S^{\left(2,0\right)}$. Restricting our model to singles and doubles (cf. Equation (5)), we have the following for the one-valence sector:

(19) $$S^{\left(1,0\right)}=S_{1}^{\left(1,0\right)}+S_{2}^{\left(1,0\right)}$$

and for the two-valence one:

(20) $$S^{\left(2,0\right)}=S_{2}^{\left(2,0\right)}$$

After solving the FS equations for the (0,0) sector, we may further simplify the equations for the (1,0) and (2,0) sectors by replacing the H operator with a similarity transformed Hamiltonian $\bar{H}$ defined as

(21) $$\bar{H}=e^{-S^{\left(0,0\right)}}He^{S^{\left(0,0\right)}}=e^{-T}He^{T}$$

Equations (17) and (18) now take form

(22) $$\begin{array}{ccc} {\hat{Q}}^{\left(1,0\right)}\bar{H}e^{S^{\left(1,0\right)}}{\hat{P}}^{\left(1,0\right)} & = & {\hat{Q}}^{\left(1,0\right)}e^{S^{\left(1,0\right)}}H_{eff}{\hat{P}}^{\left(1,0\right)} \end{array}$$

(23) $$\begin{array}{ccc} {\hat{Q}}^{\left(2,0\right)}\bar{H}e^{S^{\left(1,0\right)}+S^{\left(2,0\right)}}{\hat{P}}^{\left(2,0\right)} & = & {\hat{Q}}^{\left(2,0\right)}e^{S^{\left(1,0\right)}+S^{\left(2,0\right)}}H_{eff}{\hat{P}}^{\left(2,0\right)} \end{array}$$

The FS-CC method described here is usually referred to as a standard or $H_{eff}$-based scheme. In this approach, at each stage, one can encounter real problems with convergence due to the appearance of intruder states [43,44], i.e., determinants belonging to the orthogonal space with energies very close to those of the model determinants. Hence, the choice of the active space is, in fact, limited to small spaces. The larger the active space, the more difficult the solution is.

The intermediate Hamiltonian formalism [45,46,47] provides a way to avoid the troublesome iterative procedure of the FS problems and provides eigenvalues identical to those from the standard effective Hamiltonian-based method. Thus, the IH scheme can replace the iterative solution of the FS equations with a diagonalization of the properly constructed matrix; thanks to this, all limitations originating from the standard formulation disappear.

The main idea of the IH approach relies on selecting a part of the orthogonal space $M_{⊥}$ as an intermediate space $M_{I}$ connected by the ${\hat{P}}_{I}$ projector. In our case, the intermediate space is spanned by all determinants which are obtained by the direct action of $S_{2}^{\left(2,0\right)}$ on $M^{\left(2,0\right)}$. For the IH-FS-CCSD(2,0) case [23], we have the subspaces $M^{\left(2,0\right)}$, $M_{I}^{\left(2,0\right)}$, and $M_{I⊥}^{\left(2,0\right)}$ with the projectors ${\hat{P}}^{\left(2,0\right)}$, ${\hat{P}}_{I}^{\left(2,0\right)}$, and ${\hat{Q}}_{0}^{\left(2,0\right)}$, respectively. The projection operators are related through the following relations:

(24) $${\hat{P}}_{0}^{\left(2,0\right)}={\hat{P}}^{\left(2,0\right)}+{\hat{P}}_{I}^{\left(2,0\right)}$$

(25) $${\hat{Q}}^{\left(2,0\right)}={\hat{P}}_{I}^{\left(2,0\right)}+{\hat{Q}}_{0}^{\left(2,0\right)}$$

In this approach, we introduce the following wave operators:

(26) $$X^{\left(2,0\right)}=\left\{e^{{\tilde{S}}^{\left(2,0\right)}}-1\right\}{\hat{P}}^{\left(2,0\right)}$$

(27) $$Z^{\left(2,0\right)}=P_{I}^{\left(2,0\right)}X^{\left(2,0\right)}{\hat{P}}^{\left(2,0\right)}$$

(28) $$Y^{\left(2,0\right)}=Q_{0}^{\left(2,0\right)}X^{\left(2,0\right)}{\hat{P}}^{\left(2,0\right)}$$

(29) $$X^{\left(2,0\right)}=Z^{\left(2,0\right)}+Y^{\left(2,0\right)}$$

which, at the IH-FS-CCSD(2,0) level take the forms

(30) $$\begin{array}{ccc} Z^{\left(2,0\right)} & = & {\hat{P}}_{I}^{\left(2,0\right)}\left\{\left(S_{1}^{\left(1,0\right)}+S_{1}^{\left(1,0\right)}S_{1}^{\left(1,0\right)}+S_{2}^{\left(2,0\right)}\right)\right\}{\hat{P}}^{\left(2,0\right)} \end{array}$$

(31) $$\begin{array}{ccc} Y^{\left(2,0\right)} & = & Q_{0}^{\left(2,0\right)}\left\{\left(S_{2}^{\left(1,0\right)}+S_{1}^{\left(1,0\right)}S_{2}^{\left(1,0\right)}+S_{2}^{\left(1,0\right)}S_{2}^{\left(1,0\right)}\right)\right\}{\hat{P}}^{\left(2,0\right)} \end{array}$$

finally, the intermediate Hamiltonian operator looks like:

(32) $$\begin{array}{c} H_{I}^{\left(2,0\right)}={\hat{P}}_{0}^{\left(2,0\right)}\bar{H}{\hat{P}}_{0}^{\left(2,0\right)}+{\hat{P}}_{0}^{\left(2,0\right)}\bar{H}Y^{\left(2,0\right)}{\hat{P}}^{\left(2,0\right)} \end{array}$$

with:

(33) $$\begin{array}{c} {\hat{P}}_{0}^{\left(2,0\right)}=\sum_{ab}|\Phi^{ab}\rangle \langle \Phi^{ab}| \end{array}$$

where the a,b, …indices refer to unoccupied one-particle levels. The diagonalization of $H_{I}^{\left(2,0\right)}$ over double-electron attached configurations gives eigenvalues and eigenvectors for the (2,0) problem.

Thus, in summary, the Fock space coupled-cluster approach has two realizations in the (2,0) sector. The first one, which we denote FS-CCSD(2,0), is based on solving Equations (22) and (23), and it is impractical to use due to the intruder problem. The second one, which we denote IH-FS-CCSD(2,0), relies on the construction and diagonalization of the $H_{I}^{\left(2,0\right)}$ matrix. Note that both approaches give identical solutions as far as the energies of the electronic states are concerned, and the method we use to compute the PECs and spectroscopic constants is the IH-FS-CCSD(2,0) method.

One should also mention that the Fock space approach at the one-valence level, i.e., the (1,0) and (0,1) sectors, is equivalent to the EOM-CC scheme applied to electron affinity and ionization potential cases, respectively. This equivalence means that the eigenvalues obtained via both approaches are identical, while the eigenvectors can be obtained from each other via a simple transformation. The EOM level, i.e., the final stage of the whole sequence of computational steps, is quite straightforward and relies on the diagonalization of the $\bar{H}$ operator within the configurational subspace selected in accordance with the problem under investigation. For the (1,0) sector, we have the following:

(34) $$\begin{array}{c} H_{I}^{\left(1,0\right)}={\hat{P}}_{0}^{\left(1,0\right)}\bar{H}{\hat{P}}_{0}^{\left(1,0\right)} \end{array}$$

(35) $$\begin{array}{c} {\hat{P}}_{0}^{\left(1,0\right)}=\sum_{a}|\Phi^{a}\rangle \langle \Phi^{a}|+\sum_{abi}|\Phi_{i}^{ab}\rangle \langle \Phi_{i}^{ab}| \end{array}$$

and *i* refers to the number of occupied one-particle levels. Thus, the key point here is the intermediate Hamiltonian formalism, which offers an easy way to replace the iterative solution of the standard Bloch equation, Equation (23), with the direct diagonalization of the IH matrix constructed on the basis of the amplitudes from the (1,0) sector and Equations (31) and (32), obtained via EOM method, see Equation (34). The final outcome of this is the elimination of the intruder state problem in the FS approach.

Moreover, the size-extensive Fock space approach has a built-in capability to provide, by selecting a proper FS sector, the correlated results for an altered (with respect to the Hartree–Fock (HF) reference) number of electrons. For example, the FS(m,0) sector produces results pertaining to a system with *m* electrons added to the HF function. Assuming that we take the neutral molecule as an HF reference, these results would correspond to the *m*-tuply negative anion. We may take advantage of this capability of the FS approach to choose—at the HF level—such a reference with a convenient property to dissociate into closed-shell fragments and to generate a smooth curve for the ground and excited states in the whole region of interatomic distances, keeping in mind that by selecting a proper sector of the Fock space, we will recover the original structure we want to study.

## 4. Conclusions

In this study, we performed accurate quantum–chemical calculations of the potential energy curves of a LiMg^+^ molecular cation using the multireference coupled-cluster method, the IH-FS-CCSD(2,0) method. We overcame the challenge of properly describing the dissociation of closed-shell species into open-shell parts by employing the double-electron attachment formalism. Our calculations for the ground and excited states covered, for the first time, the entire range of interatomic distances using the RHF reference function, correlating all electrons. This approach also involves the Intermediate Hamiltonian formulation, which allows for the direct diagonalization of a suitably constructed matrix, mitigating complications associated with intruder states instead of finding an iterative solution, as in the standard Bloch equation.

For the first time, the IH-FS-CCSD(2,0) formalism was used to study the PECs and spectroscopic constants of a molecular cation composed of alkali and alkaline earth metals. We obtained smooth and accurate PECs of the fifteen lowest-lying states of the LiMg^+^ cation approaching eight dissociation limits. Through this approach, we also obtained accurate spectroscopic constants. Among all previous theoretical studies, our $\omega_{e}$ value of the ground state exhibits the closest agreement with the experimental value. Furthermore, for the ground state, we obtained a level of accuracy comparable to those of CCSD(T) and CCSDT studies without the need to include the effect of triples. For the excited states, our results indicate that most of our computed values align well with those from prior theoretical papers, and observed disparities could likely be attributed to the constraints of the EE-EOM-CCSD and pseudopotential-based methods.

Based on the quality of results obtained in various prior studies involving diatomic molecules comprising alkali metals [23,24,25,48,49] using the IH-FS-CCSD(2,0) method, we anticipate that the accuracy of presented the PECs and spectroscopic constants of LiMg^+^ can be regarded as benchmark-level.

The present study aims to lay the groundwork for promising perspectives for future spectroscopic investigations of molecular cations of alkali and alkaline earth metals. It especially seeks to open a path for further theoretical and experimental studies on LiMg^+^, mainly in the context of research on ultracold species where accurate PECs and spectroscopic constants are much needed. A wide range of potential applications of these molecular cations, e.g., in the studies of ultracold chemical reactions, quantum information processing, or measurements of fundamental physical constants, will be of great importance in the future.

## Figures and Tables

**Table 1:** Additional diffuse functions for the Li and Mg atoms in the unANO-RCC+ basis set for the s, p, and d shells.

s	p	d
Li		
0.0027497	0.0017173	0.0067528
0.0009619	0.0006010	0.0023635
Mg		
0.0062002	0.0053322	0.0209515
0.0024801	0.0021329	0.0083806

**Table 2:** Energies of electronic states at dissociation limit of LiMg^+^ molecular cation compared to atomic energies in different basis sets.

Diss. Limit	Li/Li^+^		Mg/Mg^+^		Li/Li^+^ + Mg/Mg^+^	LiMg^+^ R = *∞*
	IH-FS-CCSD(1,0)/CCSD		IH-FS-CCSD(2,0)/IH-FS-CCSD(1,0)			IH-FS-CCSD(2,0)
	Config.	E (a.u.)	Config.	E (a.u.)	E (a.u.)	E (a.u.)
unANO-RCC+						
Mg(3s^2^)^1^S+Li^+^	[He]	−7.275561	[Ne] (3s^2^${)}^{1}$S	−200.007773	−207.283334	−207.283334
Mg^+^(3s)+Li(2s)	[He] 2s	−7.473553	[Ne] 3s	−199.727651	−207.201204	−207.201204
Mg(3s3p)^3^P+Li^+^	[He]	−7.275561	[Ne] (3s3p)^3^P	−199.909054	−207.184615	−207.184615
Mg^+^(3s)+Li(2p)	[He] 2p	−7.405598	[Ne] 3s	−199.727651	−207.133249	−207.133249
Mg(3s3p)^1^P+Li^+^	[He]	−7.275561	[Ne] (3s3p)^1^P	−199.848626	−207.124187	−207.124187
Mg(3s4s)^3^S+Li^+^	[He]	−7.275561	[Ne] (3s4s)^3^S	−199.820785	−207.096346	−207.096346
Mg(3s4s)^1^S+Li^+^	[He]	−7.275561	[Ne] (3s4s)^1^S	−199.810337	−207.085898	−207.085898
Mg^+^(3s)+Li(3s)	[He] 3s	−7.349683	[Ne] 3s	−199.727651	−207.077334	−207.077334
ANO-RCC DK3						
Mg(3s^2^)^1^S+Li^+^	[He]	−7.276222	[Ne] (3s^2^${)}^{1}$S	−200.288451	−207.564673	−207.564673
Mg^+^(3s)+Li(2s)	[He] 2s	−7.474225	[Ne] 3s	−200.007961	−207.482186	−207.482186
Mg(3s3p)^3^P+Li^+^	[He]	−7.276222	[Ne] (3s3p)^3^P	−200.189246	−207.465468	−207.465468
Mg^+^(3s)+Li(2p)	[He] 2p	−7.406256	[Ne] 3s	−200.007961	−207.414217	−207.414217
Mg(3s3p)^1^P+Li^+^	[He]	−7.276222	[Ne] (3s3p)^1^P	−200.127825	−207.404047	−207.404047
Mg(3s4s)^3^S+Li^+^	[He]	−7.276222	[Ne] (3s4s)^3^S	−200.100324	−207.376546	−207.376546
Mg(3s4s)^1^S+Li^+^	[He]	−7.276222	[Ne] (3s4s)^1^S	−200.088682	−207.364904	−207.364904
Mg^+^(3s)+Li(3s)	[He] 3s	−7.346511	[Ne] 3s	−200.007961	−207.354472	−207.354472
unANO-RCC						
Mg(3s^2^)^1^S+Li^+^	[He]	−7.275560	[Ne] (3s^2^${)}^{1}$S	−200.007844	−207.283404	−207.283404
Mg^+^(3s)+Li(2s)	[He] 2s	−7.473552	[Ne] 3s	−199.727647	−207.201199	−207.201199
Mg(3s3p)^3^P+Li^+^	[He]	−7.275560	[Ne] (3s3p)^3^P	−199.909071	−207.184631	−207.184631
Mg^+^(3s)+Li(2p)	[He] 2p	−7.405596	[Ne] 3s	−199.727647	−207.133243	−207.133243
Mg(3s3p)^1^P+Li^+^	[He]	−7.275560	[Ne] (3s3p)^1^P	−199.848581	−207.124141	−207.124141
Mg(3s4s)^3^S+Li^+^	[He]	−7.275560	[Ne] (3s4s)^3^S	−199.820188	−207.095748	−207.095748
Mg(3s4s)^1^S+Li^+^	[He]	−7.275560	[Ne] (3s4s)^1^S	−199.808600	−207.084160	−207.084160
Mg^+^(3s)+Li(3s)	[He] 3s	−7.349680	[Ne] 3s	−199.727647	−207.077327	−207.077327

**Table 3:** Excitation energies of Li and Mg atoms. All values are in eV.

Li				
IH-FS-CCSD(1,0)				
(2p)^2^P	(3s)^2^S	(3p)^2^P	(3d)^2^D	Source/Basis Set
1.848	3.373	3.834	3.879	Exp. [38]
1.849	3.371	3.834	3.876	unANO-RCC+
1.850	3.475	4.392	3.963	ANO-RCC DK3
1.849	3.371	3.835	3.963	unANO-RCC
**Mg**				
**IH-FS-CCSD(2,0)**				
**(3s3p)^3^P**	**(3s3p)^1^P**	**(3s4s)^3^S**	**(3s4s)^1^S**	**Source/Basis Set**
2.711	4.346	5.108	5.394	Exp. [38]
2.686	4.331	5.088	5.372	unANO-RCC+
2.700	4.371	5.119	5.436	ANO-RCC DK3
2.688	4.334	5.106	5.422	unANO-RCC

**Table 4:** Avoided-crossing positions of LiMg^+^ cation obtained using IH-FS-CCSD(2,0)/unANO-RCC+ in comparison with Ref. [8].

States	Position in This Work (Å)	Position in [8] (Å)
$3^{1}\Sigma^{+}$/$4^{1}\Sigma^{+}$	3.90	3.84
	12.26	12.22
$5^{1}\Sigma^{+}$/$6^{1}\Sigma^{+}$	6.88	6.93
	17.00	16.67
$3^{3}\Sigma^{+}$/$4^{3}\Sigma^{+}$	3.29	3.23
	10.83	11.03
$4^{3}\Sigma^{+}$/$5^{3}\Sigma^{+}$	14.39	14.14

**Table 5:** Spectroscopic constants of LiMg^+^. $D_{e}$, $T_{e}$, ω**_e_**, ω**_e_x_e_**, and $B_{e}$ are given in ${\mathrm{cm}}^{-1}$; $R_{e}$ is given in Å. DK3 relativistic corrections are given in parentheses.

Sym.	R_e_	D_e_	T_e_	ω _e_	ω_e_x_e_	B_e_	Source
Mg(3s^2^^1^S+Li^+^							
$X^{1}\Sigma^{+}$	2.898 (−0.006)	6605 (20)		266.57 (0.21)	2.28 (−0.10)	0.370 (0.002)	This work${\;}^{a}$
				268.7	2.47		Exp. [31]
	2.900	6628		266		0.369	Theor. [11]${\;}^{b}$
	2.898	6658.8		265.9	2.0		Theor. [30]${\;}^{c}$
	2.900	6649.2		265.4	2.0		Theor. [30]${\;}^{d}$
	2.905	6696.1		265.7	2.12	0.3628	Theor. [18] ^*e*^
	2.928	6557.3		266.4	2.48	0.3623	Theor. [29]${\;}^{f}$
	2.895	6575		264.22	2.63	0.372138	Theor. [8]${\;}^{g}$
Mg^+^(3s)+Li(2s)							
$2^{1}\Sigma^{+}$	5.727 (−0.007)	1052 (1)	23,642 (81)	77.56 (0.19)	1.49 (0.01)	0.095 (0.001)	This work${\;}^{a}$
	7.300	283.1	24,618.03	40.0	1.97	0.0595	Theor. [18] ^*e*^
	5.678	1248	23,647	79.59	1.12	0.096860	Theor. [8]${\;}^{g}$
$1^{3}\Sigma^{+}$	3.516 (−0.006)	7823 (−18)	16,870 (100)	189.53 (−0.67)	0.91 (−0.02)	0.251 (0.001)	This work${\;}^{a}$
	3.573	6908.1	17,992.74	179.5	0.45	0.2347	Theor. [18] ^*e*^
	3.546	7679.4	16,441.5	187.8	0.84	0.2470	Theor. [29]${\;}^{f}$
	3.514	7983	16,912	189.96	1.43	0.252539	Theor. [8]${\;}^{g}$
Mg(3s3p)^3^P+Li^+^							
$2^{3}\Sigma^{+}$	7.433 (−0.007)	806 (−10)	27,560 (125)	52.45 (−0.29)	1.03 (0.01)	0.056 (0.000)	This work${\;}^{a}$
	7.387	877	27,692	52.57	1.38	0.057214	Theor. [8]${\;}^{g}$
$1^{3}\Pi$	2.968 (−0.002)	2588 (3)	25,554 (−111)	210.54 (1.92)	3.55 (0.02)	0.353 (0.001)	This work${\;}^{a}$
	2.955	2578.6	26,824.62	215.4	4.38	0.3567	Theor. [18] ^*e*^
	2.990	2822.9	25,070.0	212.0	3.61	0.3475	Theor. [29]${\;}^{f}$
	2.963	2561	26,008	206.32	3.51	0.356099	Theor. [8]${\;}^{g}$
Mg^+^(3s)+Li(2p)							
$3^{1}\Sigma^{+}$	6.659 (−0.020)	2441 (−23)	37,169 (108)	70.10 (0.77)	0.32 (0.01)	0.070 (0.000)	This work${\;}^{a}$
	7.408	938.9	38,691.4	61.7	1.39	0.0573	Theor. [18] ^*e*^
	6.657	2548	37,252	70.38	0.48	0.070509	Theor. [8]${\;}^{g}$
$3^{3}\Sigma^{+}$	8.172 (−0.012)	826 (−24)	38,784 (108)	45.17 (−0.03)	0.64 (0.03)	0.047 (0.001)	This work${\;}^{a}$
	8.043	916	38,884	46.98	0.53	0.048258	Theor. [8]${\;}^{g}$
$1^{1}\Pi$	3.564 (0.001)	1438 (−32)	38,171 (116)	71.83 (−0.17)	0.21 (0.10)	0.244 (−0.001)	This work${\;}^{a}$
	3.778	1418	38,383	55.48	1.32	0.218563	Theor. [8]${\;}^{g}$
	3.482	1540.5	37,852.2	87.3	0.57	0.2547	Theor. [29]${\;}^{f}$
$2^{3}\Pi$	Repulsive						This work${\;}^{a}$
	7.615	2	39,799	13.98	16.26	0.055180	Theor. [8]${\;}^{g}$
Mg(3s3p)^1^P+Li^+^							
$4^{1}\Sigma^{+}$	4.140 (−0.009)	−4148 (119)	45,981 (200)	135.97	-	0.181 (0.001)	This work${\;}^{a}$
1^st^ min.	4.101	204	46,132	161.01	31.76	0.185477	Theor. [8]${\;}^{g}$
$4^{1}\Sigma^{+}$	10.067 (−0.394)	604 (33)	41,229 (285)	37.61 (5.61)	0.63 (0.12)	0.031 (0.003)	This work${\;}^{a}$
2^nd^ min.	10.335	644	41,250	34.52	0.46	0.029241	Theor. [8]${\;}^{g}$
$2^{1}\Pi$	3.870 (−0.003)	−1779 (135)	43,609 (180)	98.89	-	0.207 (0.000)	This work${\;}^{a}$
	3.884	54	43,734	95.55	42.26	0.205913	Theor. [8]${\;}^{g}$
Mg(3s4s)^3^S+Li^+^							
$4^{3}\Sigma^{+}$	9.807 (−0.431)	861 (−483)	46,887 (606)	49.15 (4.70)	0.51 (0.25)	0.032 (0.002)	This work${\;}^{a}$
	10.187	1297	46,433	41.99	0.01	0.030084	Theor. [8]${\;}^{g}$
Mg(3s4s)^1^S+Li^+^							
$5^{1}\Sigma^{+}$	10.299 (−0.665)	955 (−647)	49,098 (782)	48.94 (7.10)	0.35 (0.17)	0.029 (0.003)	This work${\;}^{a}$
	10.933	1524	48,523	40.12	0.50	0.026131	Theor. [8] ${\;}^{g}$
Mg^+^(3s)+Li(3s)							
$6^{1}\Sigma^{+}$	13.603 (−1.461)	446 (−267)	52,275 (1191)	22.27 (−1.16)	0.42 (0.26)	0.017 (0.003)	This work${\;}^{a}$
	14.78	807	51,300	24.61	0.15	0.014297	Theor. [8]${\;}^{g}$
$5^{3}\Sigma^{+}$	12.011 (−0.974)	772 (−598)	51,950 (1523)	25.94 (−5.43)	0.40 (0.19)	0.022 (0.004)	This work${\;}^{a}$
	12.801	1505	50,601	31.69	0.18	0.019050	Theor. [8] ${\;}^{g}$

^*a*^ the method used in this work, which is IH-FS-CCSD(2,0)/unANO-RCC+ plus DK3 relativistic correction, as described in detail in the text. *^b^* the method used in [11] is CCSD(T)/aug-cc-pCVQZ. ^*c*^ the first method used in [30] is CCSDT/cc-pCVQZ. *^d^* the second method used in [30] is MRCI/cc-pCVQZ. *^e^* the method used in [18] is CCSD(T)/cc-pVQZ with a relativistic correction for the ground state and EE-EOM-CCSD/cc-pVQZ for excited states. *^f^* the methods used in [29] are the MRCI plus Davidson and third-order Douglas–Kroll–Hess relativistic correction using the aug-cc-pV5Z basis set. *^g^* the method used in [8] is based on pseudopotentials. The basis set for lithium was (9s8p5d/8s6p3d); for magnesium, it was (9s7p5d4f/7s7p4d4f).

## Data Availability

The data that support the findings of this study are available within the article and the Supplementary Materials.

## Supplementary Materials

The following supporting information can be downloaded at https://www.mdpi.com/article/10.3390/molecules29102364/s1, Table S1: Total IH-FS-CCSD (2,0)/unANO-RCC+ energies for the $X^{1}\Sigma^{+}$ state of LiMg^+^; Table S2: Total IH-FS-CCSD (2,0)/unANO-RCC+ energies for the $2^{1}\Sigma^{+}$ state of LiMg^+^; Table S3: Total IH-FS-CCSD (2,0)/unANO-RCC+ energies for the $3^{1}\Sigma^{+}$ state of LiMg^+^; Table S4: Total IH-FS-CCSD (2,0)/unANO-RCC+ energies for the $4^{1}\Sigma^{+}$ state of LiMg^+^; Table S5: Total IH-FS-CCSD (2,0)/unANO-RCC+ energies for the $5^{1}\Sigma^{+}$ state of LiMg^+^; Table S6: Total IH-FS-CCSD (2,0)/unANO-RCC+ energies for the $6^{1}\Sigma^{+}$ state of LiMg^+^; Table S7: Total IH-FS-CCSD (2,0)/unANO-RCC+ energies for the $1^{3}\Sigma^{+}$ state of LiMg^+^; Table S8: Total IH-FS-CCSD (2,0)/unANO-RCC+ energies for the $2^{3}\Sigma^{+}$ state of LiMg^+^; Table S9: Total IH-FS-CCSD (2,0)/unANO-RCC+ energies for the $3^{3}\Sigma^{+}$ state of LiMg^+^; Table S10: Total IH-FS-CCSD (2,0)/unANO-RCC+ energies for the $4^{3}\Sigma^{+}$ state of LiMg^+^; Table S11: Total IH-FS-CCSD (2,0)/unANO-RCC+ energies for the $5^{3}\Sigma^{+}$ state of LiMg^+^; Table S12: Total IH-FS-CCSD (2,0)/unANO-RCC+ energies for the $1^{1}\Pi$ state of LiMg^+^; Table S13: Total IH-FS-CCSD (2,0)/unANO-RCC+ energies for the $2^{1}\Pi$ state of LiMg^+^; Table S14: Total IH-FS-CCSD (2,0)/unANO-RCC+ energies for the $1^{3}\Pi$ state of LiMg^+^; Table S15: Total IH-FS-CCSD (2,0)/unANO-RCC+ energies for the $2^{3}\Pi$ state of LiMg^+^; Table S16: Total IH-FS-CCSD (2,0) DK3/ANO-RCC energies for the $X^{1}\Sigma^{+}$ state of LiMg^+^; Table S17: Total IH-FS-CCSD (2,0) DK3/ANO-RCC energies for the $2^{1}\Sigma^{+}$ state of LiMg^+^; Table S18: Total IH-FS-CCSD (2,0) DK3/ANO-RCC energies for the $3^{1}\Sigma^{+}$ state of LiMg^+^; Table S19: Total IH-FS-CCSD (2,0) DK3/ANO-RCC energies for the $4^{1}\Sigma^{+}$ state of LiMg^+^; Table S20: Total IH-FS-CCSD (2,0) DK3/ANO-RCC energies for the $5^{1}\Sigma^{+}$ state of LiMg^+^; Table S21: Total IH-FS-CCSD (2,0) DK3/ANO-RCC energies for the $6^{1}\Sigma^{+}$ state of LiMg^+^; Table S22: Total IH-FS-CCSD (2,0) DK3/ANO-RCC energies for the $1^{3}\Sigma^{+}$ state of LiMg^+^; Table S23: Total IH-FS-CCSD (2,0) DK3/ANO-RCC energies for the $2^{3}\Sigma^{+}$ state of LiMg^+^; Table S24: Total IH-FS-CCSD (2,0) DK3/ANO-RCC energies for the $3^{3}\Sigma^{+}$ state of LiMg^+^; Table S25: Total IH-FS-CCSD (2,0) DK3/ANO-RCC energies for the $4^{3}\Sigma^{+}$ state of LiMg^+^; Table S26: Total IH-FS-CCSD (2,0) DK3/ANO-RCC energies for the $5^{3}\Sigma^{+}$ state of LiMg^+^; Table S27: Total IH-FS-CCSD (2,0) DK3/ANO-RCC energies for the $1^{1}\Pi$ state of LiMg^+^; Table S28: Total IH-FS-CCSD (2,0) DK3/ANO-RCC energies for the $2^{1}\Pi$ state of LiMg^+^; Table S29: Total IH-FS-CCSD (2,0) DK3/ANO-RCC energies for the $1^{3}\Pi$ state of LiMg^+^; Table S30: Total IH-FS-CCSD (2,0) DK3/ANO-RCC energies for the $2^{3}\Pi$ state of LiMg^+^.
