# Supplementary material for: An Intruder-Free Fock Space Coupled-Cluster Study of the Potential Energy Curves of LiMg+ within the (2,0) Sector
Source: Molecules. 2024 May 17;29(10):2364. doi: 10.3390/molecules29102364 (PMC11124300; doi:10.3390/molecules29102364)
Supplement: Supplementary file 1 [file molecules-29-02364-s001.zip › molecules-2995269-supplementary.pdf]

# Supplementary Material for

# An Intruder-Free Fock Space Coupled-Cluster Study of the Potential Energy Curves of $LiMg^+$ within the (2,0) Sector

Grzegorz Skrzyński[*], Monika Musial[†]
*Institute of Chemistry*
*University of Silesia in Katowice*
*Szkolna 9, 40-006 Katowice, Poland*

April 18, 2024

[*]Corresponding author: e-mail address: grzegorz.skrzynski@us.edu.pl
[†]Corresponding author: e-mail address: monika.musial@us.edu.pl

Table S1: Total IH-FS-CCSD (2,0)/unANO-RCC+ energies for the $X^1\Sigma^+$ state of $LiMg^+$.

| R (Å) | E (a.u.) | R (Å) | E (a.u.) | R (Å) | E (a.u.) | R (Å) | E (a.u.) |
|---|---|---|---|---|---|---|---|
| 1.4 | -207.067712 | 8.0 | -207.284103 | 19.2 | -207.283351 | 30.4 | -207.283336 |
| 1.5 | -207.117425 | 8.2 | -207.284026 | 19.4 | -207.283350 | 30.6 | -207.283336 |
| 1.6 | -207.158798 | 8.4 | -207.283959 | 19.6 | -207.283350 | 30.8 | -207.283336 |
| 1.7 | -207.193188 | 8.6 | -207.283899 | 19.8 | -207.283349 | 31.0 | -207.283336 |
| 1.8 | -207.221467 | 8.8 | -207.283847 | 20.0 | -207.283348 | 31.2 | -207.283336 |
| 1.9 | -207.244392 | 9.0 | -207.283798 | 20.2 | -207.283348 | 31.4 | -207.283336 |
| 2.0 | -207.262690 | 9.2 | -207.283759 | 20.4 | -207.283347 | 31.6 | -207.283336 |
| 2.1 | -207.277058 | 9.4 | -207.283723 | 20.6 | -207.283347 | 31.8 | -207.283336 |
| 2.2 | -207.288143 | 9.6 | -207.283692 | 20.8 | -207.283346 | 32.0 | -207.283336 |
| 2.3 | -207.296525 | 9.8 | -207.283664 | 21.0 | -207.283346 | 33.0 | -207.283336 |
| 2.4 | -207.302710 | 10.0 | -207.283638 | 21.2 | -207.283345 | 34.0 | -207.283336 |
| 2.5 | -207.307127 | 10.2 | -207.283615 | 21.4 | -207.283345 | 35.0 | -207.283335 |
| 2.6 | -207.310133 | 10.4 | -207.283594 | 21.6 | -207.283344 | 36.0 | -207.283335 |
| 2.7 | -207.312020 | 10.6 | -207.283575 | 21.8 | -207.283344 | 37.0 | -207.283335 |
| 2.8 | -207.313027 | 10.8 | -207.283557 | 22.0 | -207.283343 | 38.0 | -207.283335 |
| 2.9 | -207.313340 | 11.0 | -207.283541 | 22.2 | -207.283343 | 39.0 | -207.283335 |
| 3.0 | -207.313122 | 11.2 | -207.283527 | 22.4 | -207.283343 | 40.0 | -207.283335 |
| 3.1 | -207.312506 | 11.4 | -207.283513 | 22.6 | -207.283342 | 42.0 | -207.283335 |
| 3.2 | -207.311589 | 11.6 | -207.283489 | 22.8 | -207.283342 | 44.0 | -207.283335 |
| 3.3 | -207.310452 | 11.8 | -207.283478 | 23.0 | -207.283342 | 46.0 | -207.283335 |
| 3.4 | -207.309160 | 12.0 | -207.283468 | 23.2 | -207.283341 | 48.0 | -207.283335 |
| 3.5 | -207.307768 | 12.2 | -207.283459 | 23.4 | -207.283341 | 50.0 | -207.283335 |
| 3.6 | -207.306317 | 12.4 | -207.283451 | 23.6 | -207.283341 | 52.0 | -207.283335 |
| 3.7 | -207.304843 | 12.6 | -207.283443 | 23.8 | -207.283340 | 54.0 | -207.283335 |
| 3.8 | -207.303372 | 12.8 | -207.283436 | 24.0 | -207.283340 | 56.0 | -207.283335 |
| 3.9 | -207.301927 | 13.0 | -207.283429 | 24.2 | -207.283340 | 58.0 | -207.283335 |
| 4.0 | -207.300523 | 13.2 | -207.283423 | 24.4 | -207.283340 | 60.0 | -207.283334 |
| 4.1 | -207.299174 | 13.4 | -207.283418 | 24.6 | -207.283340 | 62.0 | -207.283334 |
| 4.2 | -207.297890 | 13.6 | -207.283413 | 24.8 | -207.283339 | 64.0 | -207.283334 |
| 4.3 | -207.296678 | 13.8 | -207.283408 | 25.0 | -207.283339 | 66.0 | -207.283334 |
| 4.4 | -207.295540 | 14.0 | -207.283403 | 25.2 | -207.283339 | 68.0 | -207.283334 |
| 4.5 | -207.294480 | 14.2 | -207.283399 | 25.4 | -207.283339 | 70.0 | -207.283334 |
| 4.6 | -207.293498 | 14.4 | -207.283395 | 25.6 | -207.283339 | 72.0 | -207.283334 |
| 4.7 | -207.292593 | 14.6 | -207.283392 | 25.8 | -207.283338 | 74.0 | -207.283334 |
| 4.8 | -207.291763 | 14.8 | -207.283388 | 26.0 | -207.283338 | 76.0 | -207.283334 |
| 4.9 | -207.291005 | 15.0 | -207.283385 | 26.2 | -207.283338 | 78.0 | -207.283334 |
| 5.0 | -207.290314 | 15.2 | -207.283382 | 26.4 | -207.283338 | 80.0 | -207.283334 |
| 5.1 | -207.289687 | 15.4 | -207.283379 | 26.6 | -207.283338 | 82.0 | -207.283334 |
| 5.2 | -207.289119 | 15.6 | -207.283377 | 26.8 | -207.283338 | 84.0 | -207.283334 |
| 5.3 | -207.288605 | 15.8 | -207.283374 | 27.0 | -207.283338 | 86.0 | -207.283334 |
| 5.4 | -207.288142 | 16.0 | -207.283372 | 27.2 | -207.283338 | 88.0 | -207.283334 |
| 5.5 | -207.287723 | 16.2 | -207.283370 | 27.4 | -207.283337 | 90.0 | -207.283334 |
| 5.6 | -207.287347 | 16.4 | -207.283368 | 27.6 | -207.283337 | 92.0 | -207.283334 |
| 5.7 | -207.287008 | 16.6 | -207.283366 | 27.8 | -207.283337 | 94.0 | -207.283334 |
| 5.8 | -207.286703 | 16.8 | -207.283365 | 28.0 | -207.283337 | 96.0 | -207.283334 |
| 5.9 | -207.286428 | 17.0 | -207.283363 | 28.2 | -207.283337 | 98.0 | -207.283334 |
| 6.0 | -207.286182 | 17.2 | -207.283362 | 28.4 | -207.283337 | 100.0 | -207.283334 |
| 6.2 | -207.285760 | 17.4 | -207.283360 | 28.6 | -207.283337 | 150.0 | -207.283334 |
| 6.4 | -207.285417 | 17.6 | -207.283359 | 28.8 | -207.283337 | 200.0 | -207.283334 |
| 6.6 | -207.285136 | 17.8 | -207.283358 | 29.0 | -207.283337 | | |
| 6.8 | -207.284904 | 18.0 | -207.283357 | 29.2 | -207.283337 | | |
| 7.0 | -207.284711 | 18.2 | -207.283356 | 29.4 | -207.283337 | | |
| 7.2 | -207.284548 | 18.4 | -207.283355 | 29.6 | -207.283336 | | |
| 7.4 | -207.284410 | 18.6 | -207.283354 | 29.8 | -207.283336 | | |
| 7.6 | -207.284293 | 18.8 | -207.283353 | 30.0 | -207.283336 | | |
| 7.8 | -207.284191 | 19.0 | -207.283352 | 30.2 | -207.283336 | | |

Table S2: Total IH-FS-CCSD (2,0)/unANO-RCC+ energies for the $2^1\Sigma^+$ state of $LiMg^+$.

| R (Å) | E (a.u.) | R (Å) | E (a.u.) | R (Å) | E (a.u.) | R (Å) | E (a.u.) |
|---|---|---|---|---|---|---|---|
| 1.4 | -206.890931 | 8.0 | -207.203090 | 19.2 | -207.201252 | 30.4 | -207.201212 |
| 1.5 | -206.938611 | 8.2 | -207.202902 | 19.4 | -207.201250 | 30.6 | -207.201211 |
| 1.6 | -206.978355 | 8.4 | -207.202734 | 19.6 | -207.201248 | 30.8 | -207.201211 |
| 1.7 | -207.012132 | 8.6 | -207.202586 | 19.8 | -207.201247 | 31.0 | -207.201211 |
| 1.8 | -207.040961 | 8.8 | -207.202454 | 20.0 | -207.201245 | 31.2 | -207.201211 |
| 1.9 | -207.065422 | 9.0 | -207.202337 | 20.2 | -207.201243 | 31.4 | -207.201211 |
| 2.0 | -207.085940 | 9.2 | -207.202233 | 20.4 | -207.201242 | 31.6 | -207.201210 |
| 2.1 | -207.102927 | 9.4 | -207.202141 | 20.6 | -207.201240 | 31.8 | -207.201210 |
| 2.2 | -207.116814 | 9.6 | -207.202059 | 20.8 | -207.201239 | 32.0 | -207.201210 |
| 2.3 | -207.128042 | 9.8 | -207.201986 | 21.0 | -207.201238 | 33.0 | -207.201209 |
| 2.4 | -207.137036 | 10.0 | -207.201921 | 21.2 | -207.201236 | 34.0 | -207.201209 |
| 2.5 | -207.144195 | 10.2 | -207.201862 | 21.4 | -207.201235 | 35.0 | -207.201208 |
| 2.6 | -207.149884 | 10.4 | -207.201810 | 21.6 | -207.201234 | 36.0 | -207.201208 |
| 2.7 | -207.154433 | 10.6 | -207.201762 | 21.8 | -207.201233 | 37.0 | -207.201207 |
| 2.8 | -207.158137 | 10.8 | -207.201719 | 22.0 | -207.201232 | 38.0 | -207.201207 |
| 2.9 | -207.161257 | 11.0 | -207.201681 | 22.2 | -207.201231 | 39.0 | -207.201207 |
| 3.0 | -207.164022 | 11.2 | -207.201646 | 22.4 | -207.201230 | 40.0 | -207.201206 |
| 3.1 | -207.166618 | 11.4 | -207.201614 | 22.6 | -207.201229 | 42.0 | -207.201206 |
| 3.2 | -207.169176 | 11.6 | -207.201585 | 22.8 | -207.201228 | 44.0 | -207.201206 |
| 3.3 | -207.171777 | 11.8 | -207.201559 | 23.0 | -207.201227 | 46.0 | -207.201205 |
| 3.4 | -207.174448 | 12.0 | -207.201535 | 23.2 | -207.201226 | 48.0 | -207.201205 |
| 3.5 | -207.177177 | 12.2 | -207.201513 | 23.4 | -207.201226 | 50.0 | -207.201205 |
| 3.6 | -207.179927 | 12.4 | -207.201493 | 23.6 | -207.201225 | 52.0 | -207.201205 |
| 3.7 | -207.182649 | 12.6 | -207.201474 | 23.8 | -207.201224 | 54.0 | -207.201205 |
| 3.8 | -207.185297 | 12.8 | -207.201457 | 24.0 | -207.201224 | 56.0 | -207.201205 |
| 3.9 | -207.187830 | 13.0 | -207.201441 | 24.2 | -207.201223 | 58.0 | -207.201204 |
| 4.0 | -207.190215 | 13.2 | -207.201427 | 24.4 | -207.201222 | 60.0 | -207.201204 |
| 4.1 | -207.192430 | 13.4 | -207.201413 | 24.6 | -207.201222 | 62.0 | -207.201204 |
| 4.2 | -207.194462 | 13.6 | -207.201401 | 24.8 | -207.201221 | 64.0 | -207.201204 |
| 4.3 | -207.196302 | 13.8 | -207.201390 | 25.0 | -207.201221 | 66.0 | -207.201204 |
| 4.4 | -207.197950 | 14.0 | -207.201379 | 25.2 | -207.201220 | 68.0 | -207.201204 |
| 4.5 | -207.199410 | 14.2 | -207.201369 | 25.4 | -207.201220 | 70.0 | -207.201204 |
| 4.6 | -207.200687 | 14.4 | -207.201360 | 25.6 | -207.201219 | 72.0 | -207.201204 |
| 4.7 | -207.201791 | 14.6 | -207.201351 | 25.8 | -207.201219 | 74.0 | -207.201204 |
| 4.8 | -207.202734 | 14.8 | -207.201343 | 26.0 | -207.201218 | 76.0 | -207.201204 |
| 4.9 | -207.203527 | 15.0 | -207.201336 | 26.2 | -207.201218 | 78.0 | -207.201204 |
| 5.0 | -207.204183 | 15.2 | -207.201329 | 26.4 | -207.201217 | 80.0 | -207.201204 |
| 5.1 | -207.204715 | 15.4 | -207.201322 | 26.6 | -207.201217 | 82.0 | -207.201204 |
| 5.2 | -207.205135 | 15.6 | -207.201316 | 26.8 | -207.201217 | 84.0 | -207.201204 |
| 5.3 | -207.205458 | 15.8 | -207.201311 | 27.0 | -207.201216 | 86.0 | -207.201204 |
| 5.4 | -207.205693 | 16.0 | -207.201305 | 27.2 | -207.201216 | 88.0 | -207.201204 |
| 5.5 | -207.205853 | 16.2 | -207.201300 | 27.4 | -207.201215 | 90.0 | -207.201204 |
| 5.6 | -207.205948 | 16.4 | -207.201296 | 27.6 | -207.201215 | 92.0 | -207.201204 |
| 5.7 | -207.205988 | 16.6 | -207.201291 | 27.8 | -207.201215 | 94.0 | -207.201204 |
| 5.8 | -207.205982 | 16.8 | -207.201287 | 28.0 | -207.201215 | 96.0 | -207.201204 |
| 5.9 | -207.205937 | 17.0 | -207.201283 | 28.2 | -207.201214 | 98.0 | -207.201204 |
| 6.0 | -207.205860 | 17.2 | -207.201279 | 28.4 | -207.201214 | 100.0 | -207.201204 |
| 6.2 | -207.205635 | 17.4 | -207.201276 | 28.6 | -207.201214 | 150.0 | -207.201204 |
| 6.4 | -207.205349 | 17.6 | -207.201273 | 28.8 | -207.201213 | 200.0 | -207.201204 |
| 6.6 | -207.205032 | 17.8 | -207.201270 | 29.0 | -207.201213 | | |
| 6.8 | -207.204705 | 18.0 | -207.201267 | 29.2 | -207.201213 | | |
| 7.0 | -207.204385 | 18.2 | -207.201264 | 29.4 | -207.201213 | | |
| 7.2 | -207.204080 | 18.4 | -207.201261 | 29.6 | -207.201212 | | |
| 7.4 | -207.203796 | 18.6 | -207.201259 | 29.8 | -207.201212 | | |
| 7.6 | -207.203536 | 18.8 | -207.201257 | 30.0 | -207.201212 | | |
| 7.8 | -207.203301 | 19.0 | -207.201254 | 30.2 | -207.201212 | | |

Table S3: Total IH-FS-CCSD (2,0)/unANO-RCC+ energies for the $3^1\Sigma^+$ state of $LiMg^+$.

| R (Å) | E (a.u.) | R (Å) | E (a.u.) | R (Å) | E (a.u.) | R (Å) | E (a.u.) |
|---|---|---|---|---|---|---|---|
| 1.4 | -206.862316 | 8.0 | -207.142261 | 19.2 | -207.133519 | 30.4 | -207.133312 |
| 1.5 | -206.898835 | 8.2 | -207.141702 | 19.4 | -207.133510 | 30.6 | -207.133311 |
| 1.6 | -206.927399 | 8.4 | -207.141126 | 19.6 | -207.133502 | 30.8 | -207.133309 |
| 1.7 | -206.959124 | 8.6 | -207.140545 | 19.8 | -207.133494 | 31.0 | -207.133308 |
| 1.8 | -206.988447 | 8.8 | -207.139968 | 20.0 | -207.133486 | 31.2 | -207.133307 |
| 1.9 | -207.012857 | 9.0 | -207.139406 | 20.2 | -207.133478 | 31.4 | -207.133306 |
| 2.0 | -207.033020 | 9.2 | -207.138865 | 20.4 | -207.133471 | 31.6 | -207.133305 |
| 2.1 | -207.049632 | 9.4 | -207.138351 | 20.6 | -207.133464 | 31.8 | -207.133304 |
| 2.2 | -207.063331 | 9.6 | -207.137869 | 20.8 | -207.133458 | 32.0 | -207.133303 |
| 2.3 | -207.074663 | 9.8 | -207.137423 | 21.0 | -207.133451 | 33.0 | -207.133298 |
| 2.4 | -207.084086 | 10.0 | -207.137013 | 21.2 | -207.133445 | 34.0 | -207.133293 |
| 2.5 | -207.091974 | 10.2 | -207.136642 | 21.4 | -207.133440 | 35.0 | -207.133289 |
| 2.6 | -207.098623 | 10.4 | -207.136308 | 21.6 | -207.133434 | 36.0 | -207.133286 |
| 2.7 | -207.104257 | 10.6 | -207.136010 | 21.8 | -207.133429 | 37.0 | -207.133283 |
| 2.8 | -207.109042 | 10.8 | -207.135746 | 22.0 | -207.133424 | 38.0 | -207.133280 |
| 2.9 | -207.113091 | 11.0 | -207.135514 | 22.2 | -207.133419 | 39.0 | -207.133278 |
| 3.0 | -207.116463 | 11.2 | -207.135309 | 22.4 | -207.133414 | 40.0 | -207.133276 |
| 3.1 | -207.119221 | 11.4 | -207.135130 | 22.6 | -207.133409 | 42.0 | -207.133272 |
| 3.2 | -207.121409 | 11.6 | -207.134974 | 22.8 | -207.133405 | 44.0 | -207.133269 |
| 3.3 | -207.123085 | 11.8 | -207.134837 | 23.0 | -207.133401 | 46.0 | -207.133266 |
| 3.4 | -207.124330 | 12.0 | -207.134716 | 23.2 | -207.133396 | 48.0 | -207.133264 |
| 3.5 | -207.125248 | 12.2 | -207.134609 | 23.4 | -207.133392 | 50.0 | -207.133262 |
| 3.6 | -207.125952 | 12.4 | -207.134515 | 23.6 | -207.133389 | 52.0 | -207.133261 |
| 3.7 | -207.126557 | 12.6 | -207.134431 | 23.8 | -207.133385 | 54.0 | -207.133259 |
| 3.8 | -207.127157 | 12.8 | -207.134356 | 24.0 | -207.133381 | 56.0 | -207.133258 |
| 3.9 | -207.127820 | 13.0 | -207.134288 | 24.2 | -207.133378 | 58.0 | -207.133257 |
| 4.0 | -207.128578 | 13.2 | -207.134227 | 24.4 | -207.133375 | 60.0 | -207.133256 |
| 4.1 | -207.129434 | 13.4 | -207.134172 | 24.6 | -207.133371 | 62.0 | -207.133256 |
| 4.2 | -207.130370 | 13.6 | -207.134122 | 24.8 | -207.133368 | 64.0 | -207.133255 |
| 4.3 | -207.131359 | 13.8 | -207.134075 | 25.0 | -207.133365 | 66.0 | -207.133254 |
| 4.4 | -207.132375 | 14.0 | -207.134033 | 25.2 | -207.133362 | 68.0 | -207.133254 |
| 4.5 | -207.133394 | 14.2 | -207.133994 | 25.4 | -207.133360 | 70.0 | -207.133253 |
| 4.6 | -207.134400 | 14.4 | -207.133957 | 25.6 | -207.133357 | 72.0 | -207.133253 |
| 4.7 | -207.135377 | 14.6 | -207.133923 | 25.8 | -207.133354 | 74.0 | -207.133253 |
| 4.8 | -207.136317 | 14.8 | -207.133892 | 26.0 | -207.133352 | 76.0 | -207.133252 |
| 4.9 | -207.137213 | 15.0 | -207.133863 | 26.2 | -207.133349 | 78.0 | -207.133252 |
| 5.0 | -207.138060 | 15.2 | -207.133835 | 26.4 | -207.133347 | 80.0 | -207.133252 |
| 5.1 | -207.138854 | 15.4 | -207.133810 | 26.6 | -207.133345 | 82.0 | -207.133252 |
| 5.2 | -207.139595 | 15.6 | -207.133785 | 26.8 | -207.133342 | 84.0 | -207.133251 |
| 5.3 | -207.140280 | 15.8 | -207.133763 | 27.0 | -207.133340 | 86.0 | -207.133251 |
| 5.4 | -207.140910 | 16.0 | -207.133741 | 27.2 | -207.133338 | 88.0 | -207.133251 |
| 5.5 | -207.141484 | 16.2 | -207.133721 | 27.4 | -207.133336 | 90.0 | -207.133251 |
| 5.6 | -207.142002 | 16.4 | -207.133702 | 27.6 | -207.133334 | 92.0 | -207.133251 |
| 5.7 | -207.142468 | 16.6 | -207.133684 | 27.8 | -207.133332 | 94.0 | -207.133251 |
| 5.8 | -207.142881 | 16.8 | -207.133667 | 28.0 | -207.133330 | 96.0 | -207.133250 |
| 5.9 | -207.143243 | 17.0 | -207.133651 | 28.2 | -207.133329 | 98.0 | -207.133250 |
| 6.0 | -207.143554 | 17.2 | -207.133636 | 28.4 | -207.133327 | 100.0 | -207.133250 |
| 6.2 | -207.144033 | 17.4 | -207.133622 | 28.6 | -207.133325 | 150.0 | -207.133249 |
| 6.4 | -207.144332 | 17.6 | -207.133608 | 28.8 | -207.133324 | 200.0 | -207.133249 |
| 6.6 | -207.144466 | 17.8 | -207.133595 | 29.0 | -207.133322 | | |
| 6.8 | -207.144452 | 18.0 | -207.133582 | 29.2 | -207.133320 | | |
| 7.0 | -207.144308 | 18.2 | -207.133570 | 29.4 | -207.133319 | | |
| 7.2 | -207.144053 | 18.4 | -207.133559 | 29.6 | -207.133317 | | |
| 7.4 | -207.143703 | 18.6 | -207.133548 | 29.8 | -207.133316 | | |
| 7.6 | -207.143277 | 18.8 | -207.133538 | 30.0 | -207.133315 | | |
| 7.8 | -207.142791 | 19.0 | -207.133528 | 30.2 | -207.133313 | | |

Table S4: Total IH-FS-CCSD (2,0)/unANO-RCC+ energies for the $4^1\Sigma^+$ state of $LiMg^+$.

| R (Å) | E (a.u.) | R (Å) | E (a.u.) | R (Å) | E (a.u.) | R (Å) | E (a.u.) |
|---|---|---|---|---|---|---|---|
| 1.4 | -206.775549 | 8.0 | -207.123015 | 19.2 | -207.124586 | 30.4 | -207.124271 |
| 1.5 | -206.822770 | 8.2 | -207.123705 | 19.4 | -207.124572 | 30.6 | -207.124270 |
| 1.6 | -206.859869 | 8.4 | -207.124310 | 19.6 | -207.124558 | 30.8 | -207.124268 |
| 1.7 | -206.890990 | 8.6 | -207.124837 | 19.8 | -207.124545 | 31.0 | -207.124266 |
| 1.8 | -206.918811 | 8.8 | -207.125288 | 20.0 | -207.124533 | 31.2 | -207.124264 |
| 1.9 | -206.943579 | 9.0 | -207.125668 | 20.2 | -207.124522 | 31.4 | -207.124263 |
| 2.0 | -206.965050 | 9.2 | -207.125985 | 20.4 | -207.124510 | 31.6 | -207.124261 |
| 2.1 | -206.983412 | 9.4 | -207.126242 | 20.6 | -207.124500 | 31.8 | -207.124260 |
| 2.2 | -206.999101 | 9.6 | -207.126444 | 20.8 | -207.124489 | 32.0 | -207.124258 |
| 2.3 | -207.012620 | 9.8 | -207.126594 | 21.0 | -207.124480 | 33.0 | -207.124251 |
| 2.4 | -207.024453 | 10.0 | -207.126698 | 21.2 | -207.124470 | 34.0 | -207.124245 |
| 2.5 | -207.035008 | 10.2 | -207.126760 | 21.4 | -207.124461 | 35.0 | -207.124240 |
| 2.6 | -207.044576 | 10.4 | -207.126785 | 21.6 | -207.124453 | 36.0 | -207.124235 |
| 2.7 | -207.053323 | 10.6 | -207.126780 | 21.8 | -207.124445 | 37.0 | -207.124231 |
| 2.8 | -207.061314 | 10.8 | -207.126748 | 22.0 | -207.124437 | 38.0 | -207.124228 |
| 2.9 | -207.068559 | 11.0 | -207.126695 | 22.2 | -207.124429 | 39.0 | -207.124224 |
| 3.0 | -207.075061 | 11.2 | -207.126626 | 22.4 | -207.124422 | 40.0 | -207.124221 |
| 3.1 | -207.080829 | 11.4 | -207.126545 | 22.6 | -207.124415 | 42.0 | -207.124216 |
| 3.2 | -207.085880 | 11.6 | -207.126451 | 22.8 | -207.124408 | 44.0 | -207.124212 |
| 3.3 | -207.090244 | 11.8 | -207.126355 | 23.0 | -207.124402 | 46.0 | -207.124209 |
| 3.4 | -207.093957 | 12.0 | -207.126257 | 23.2 | -207.124395 | 48.0 | -207.124206 |
| 3.5 | -207.097054 | 12.2 | -207.126159 | 23.4 | -207.124389 | 50.0 | -207.124204 |
| 3.6 | -207.099566 | 12.4 | -207.126062 | 23.6 | -207.124384 | 52.0 | -207.124202 |
| 3.7 | -207.101523 | 12.6 | -207.125967 | 23.8 | -207.124378 | 54.0 | -207.124200 |
| 3.8 | -207.102959 | 12.8 | -207.125876 | 24.0 | -207.124373 | 56.0 | -207.124198 |
| 3.9 | -207.103924 | 13.0 | -207.125789 | 24.2 | -207.124368 | 58.0 | -207.124197 |
| 4.0 | -207.104483 | 13.2 | -207.125706 | 24.4 | -207.124363 | 60.0 | -207.124196 |
| 4.1 | -207.104720 | 13.4 | -207.125627 | 24.6 | -207.124358 | 62.0 | -207.124195 |
| 4.2 | -207.104722 | 13.6 | -207.125553 | 24.8 | -207.124353 | 64.0 | -207.124194 |
| 4.3 | -207.104574 | 13.8 | -207.125483 | 25.0 | -207.124349 | 66.0 | -207.124194 |
| 4.4 | -207.104351 | 14.0 | -207.125418 | 25.2 | -207.124345 | 68.0 | -207.124193 |
| 4.5 | -207.104117 | 14.2 | -207.125356 | 25.4 | -207.124340 | 70.0 | -207.124192 |
| 4.6 | -207.103921 | 14.4 | -207.125298 | 25.6 | -207.124336 | 72.0 | -207.124192 |
| 4.7 | -207.103800 | 14.6 | -207.125244 | 25.8 | -207.124333 | 74.0 | -207.124191 |
| 4.8 | -207.103781 | 14.8 | -207.125193 | 26.0 | -207.124329 | 76.0 | -207.124191 |
| 4.9 | -207.103878 | 15.0 | -207.125146 | 26.2 | -207.124325 | 78.0 | -207.124191 |
| 5.0 | -207.104097 | 15.2 | -207.125101 | 26.4 | -207.124322 | 80.0 | -207.124190 |
| 5.1 | -207.104437 | 15.4 | -207.125059 | 26.6 | -207.124318 | 82.0 | -207.124190 |
| 5.2 | -207.104888 | 15.6 | -207.125019 | 26.8 | -207.124315 | 84.0 | -207.124190 |
| 5.3 | -207.105438 | 15.8 | -207.124982 | 27.0 | -207.124312 | 86.0 | -207.124190 |
| 5.4 | -207.106072 | 16.0 | -207.124947 | 27.2 | -207.124309 | 88.0 | -207.124189 |
| 5.5 | -207.106773 | 16.2 | -207.124913 | 27.4 | -207.124306 | 90.0 | -207.124189 |
| 5.6 | -207.107525 | 16.4 | -207.124882 | 27.6 | -207.124303 | 92.0 | -207.124189 |
| 5.7 | -207.108313 | 16.6 | -207.124853 | 27.8 | -207.124300 | 94.0 | -207.124189 |
| 5.8 | -207.109124 | 16.8 | -207.124825 | 28.0 | -207.124298 | 96.0 | -207.124189 |
| 5.9 | -207.109947 | 17.0 | -207.124799 | 28.2 | -207.124295 | 98.0 | -207.124189 |
| 6.0 | -207.110774 | 17.2 | -207.124774 | 28.4 | -207.124293 | 100.0 | -207.124188 |
| 6.2 | -207.112406 | 17.4 | -207.124750 | 28.6 | -207.124290 | 150.0 | -207.124187 |
| 6.4 | -207.113978 | 17.6 | -207.124728 | 28.8 | -207.124288 | 200.0 | -207.124187 |
| 6.6 | -207.115466 | 17.8 | -207.124707 | 29.0 | -207.124286 | | |
| 6.8 | -207.116857 | 18.0 | -207.124687 | 29.2 | -207.124283 | | |
| 7.0 | -207.118142 | 18.2 | -207.124668 | 29.4 | -207.124281 | | |
| 7.2 | -207.119321 | 18.4 | -207.124650 | 29.6 | -207.124279 | | |
| 7.4 | -207.120394 | 18.6 | -207.124633 | 29.8 | -207.124277 | | |
| 7.6 | -207.121365 | 18.8 | -207.124616 | 30.0 | -207.124275 | | |
| 7.8 | -207.122237 | 19.0 | -207.124601 | 30.2 | -207.124273 | | |

Table S5: Total IH-FS-CCSD (2,0)/unANO-RCC+ energies for the $5^1\Sigma^+$ state of $LiMg^+$.

| R (Å) | E (a.u.) | R (Å) | E (a.u.) | R (Å) | E (a.u.) | R (Å) | E (a.u.) |
|---|---|---|---|---|---|---|---|
| 1.4 | -206.743975 | 8.0 | -207.085882 | 19.2 | -207.086519 | 30.4 | -207.085974 |
| 1.5 | -206.795021 | 8.2 | -207.086880 | 19.4 | -207.086485 | 30.6 | -207.085972 |
| 1.6 | -206.836864 | 8.4 | -207.087811 | 19.6 | -207.086454 | 30.8 | -207.085970 |
| 1.7 | -206.870133 | 8.6 | -207.088669 | 19.8 | -207.086425 | 31.0 | -207.085968 |
| 1.8 | -206.895716 | 8.8 | -207.089451 | 20.0 | -207.086398 | 31.2 | -207.085966 |
| 1.9 | -206.916212 | 9.0 | -207.090150 | 20.2 | -207.086374 | 31.4 | -207.085964 |
| 2.0 | -206.934577 | 9.2 | -207.090778 | 20.4 | -207.086351 | 31.6 | -207.085963 |
| 2.1 | -206.952220 | 9.4 | -207.091327 | 20.6 | -207.086329 | 31.8 | -207.085961 |
| 2.2 | -206.968397 | 9.6 | -207.091801 | 20.8 | -207.086309 | 32.0 | -207.085959 |
| 2.3 | -206.982585 | 9.8 | -207.092200 | 21.0 | -207.086290 | 33.0 | -207.085952 |
| 2.4 | -206.994795 | 10.0 | -207.092528 | 21.2 | -207.086273 | 34.0 | -207.085946 |
| 2.5 | -207.005170 | 10.2 | -207.092786 | 21.4 | -207.086256 | 35.0 | -207.085940 |
| 2.6 | -207.013881 | 10.4 | -207.092979 | 21.6 | -207.086241 | 36.0 | -207.085936 |
| 2.7 | -207.021133 | 10.6 | -207.093109 | 21.8 | -207.086226 | 37.0 | -207.085932 |
| 2.8 | -207.027151 | 10.8 | -207.093180 | 22.0 | -207.086212 | 38.0 | -207.085928 |
| 2.9 | -207.032161 | 11.0 | -207.093197 | 22.2 | -207.086199 | 39.0 | -207.085925 |
| 3.0 | -207.036383 | 11.2 | -207.093163 | 22.4 | -207.086187 | 40.0 | -207.085922 |
| 3.1 | -207.039996 | 11.4 | -207.093084 | 22.6 | -207.086176 | 42.0 | -207.085918 |
| 3.2 | -207.043141 | 11.6 | -207.092962 | 22.8 | -207.086165 | 44.0 | -207.085915 |
| 3.3 | -207.045928 | 11.8 | -207.092806 | 23.0 | -207.086154 | 46.0 | -207.085912 |
| 3.4 | -207.048443 | 12.0 | -207.092618 | 23.2 | -207.086144 | 48.0 | -207.085910 |
| 3.5 | -207.050749 | 12.2 | -207.092404 | 23.4 | -207.086135 | 50.0 | -207.085908 |
| 3.6 | -207.052891 | 12.4 | -207.092168 | 23.6 | -207.086126 | 52.0 | -207.085906 |
| 3.7 | -207.054904 | 12.6 | -207.091915 | 23.8 | -207.086118 | 54.0 | -207.085905 |
| 3.8 | -207.056810 | 12.8 | -207.091649 | 24.0 | -207.086109 | 56.0 | -207.085904 |
| 3.9 | -207.058625 | 13.0 | -207.091374 | 24.2 | -207.086102 | 58.0 | -207.085904 |
| 4.0 | -207.060359 | 13.2 | -207.091094 | 24.4 | -207.086094 | 60.0 | -207.085903 |
| 4.1 | -207.062016 | 13.4 | -207.090810 | 24.6 | -207.086087 | 62.0 | -207.085902 |
| 4.2 | -207.063597 | 13.6 | -207.090528 | 24.8 | -207.086081 | 64.0 | -207.085902 |
| 4.3 | -207.065099 | 13.8 | -207.090248 | 25.0 | -207.086074 | 66.0 | -207.085901 |
| 4.4 | -207.066518 | 14.0 | -207.089973 | 25.2 | -207.086068 | 68.0 | -207.085901 |
| 4.5 | -207.067846 | 14.2 | -207.089706 | 25.4 | -207.086063 | 70.0 | -207.085901 |
| 4.6 | -207.069074 | 14.4 | -207.089446 | 25.6 | -207.086057 | 72.0 | -207.085900 |
| 4.7 | -207.070195 | 14.6 | -207.089197 | 25.8 | -207.086052 | 74.0 | -207.085900 |
| 4.8 | -207.071204 | 14.8 | -207.088960 | 26.0 | -207.086047 | 76.0 | -207.085900 |
| 4.9 | -207.072097 | 15.0 | -207.088734 | 26.2 | -207.086042 | 78.0 | -207.085900 |
| 5.0 | -207.072879 | 15.2 | -207.088521 | 26.4 | -207.086037 | 80.0 | -207.085900 |
| 5.1 | -207.073553 | 15.4 | -207.088321 | 26.6 | -207.086033 | 82.0 | -207.085900 |
| 5.2 | -207.074131 | 15.6 | -207.088134 | 26.8 | -207.086028 | 84.0 | -207.085899 |
| 5.3 | -207.074626 | 15.8 | -207.087961 | 27.0 | -207.086024 | 86.0 | -207.085899 |
| 5.4 | -207.075050 | 16.0 | -207.087801 | 27.2 | -207.086020 | 88.0 | -207.085899 |
| 5.5 | -207.075418 | 16.2 | -207.087654 | 27.4 | -207.086016 | 90.0 | -207.085899 |
| 5.6 | -207.075744 | 16.4 | -207.087520 | 27.6 | -207.086013 | 92.0 | -207.085899 |
| 5.7 | -207.076039 | 16.6 | -207.087396 | 27.8 | -207.086009 | 94.0 | -207.085899 |
| 5.8 | -207.076315 | 16.8 | -207.087284 | 28.0 | -207.086006 | 96.0 | -207.085899 |
| 5.9 | -207.076582 | 17.0 | -207.087182 | 28.2 | -207.086003 | 98.0 | -207.085899 |
| 6.0 | -207.076846 | 17.2 | -207.087090 | 28.4 | -207.086000 | 100.0 | -207.085899 |
| 6.2 | -207.077394 | 17.4 | -207.087005 | 28.6 | -207.085997 | 150.0 | -207.085898 |
| 6.4 | -207.078005 | 17.6 | -207.086929 | 28.8 | -207.085994 | 200.0 | -207.085898 |
| 6.6 | -207.078715 | 17.8 | -207.086860 | 29.0 | -207.085991 | | |
| 6.8 | -207.079543 | 18.0 | -207.086797 | 29.2 | -207.085988 | | |
| 7.0 | -207.080489 | 18.2 | -207.086739 | 29.4 | -207.085986 | | |
| 7.2 | -207.081528 | 18.4 | -207.086687 | 29.6 | -207.085983 | | |
| 7.4 | -207.082622 | 18.6 | -207.086639 | 29.8 | -207.085981 | | |
| 7.6 | -207.083733 | 18.8 | -207.086596 | 30.0 | -207.085978 | | |
| 7.8 | -207.084828 | 19.0 | -207.086556 | 30.2 | -207.085976 | | |

Table S6: Total IH-FS-CCSD (2,0)/unANO-RCC+ energies for the $6^1\Sigma^+$ state of $LiMg^+$.

| R (Å) | E (a.u.) | R (Å) | E (a.u.) | R (Å) | E (a.u.) | R (Å) | E (a.u.) |
|---|---|---|---|---|---|---|---|
| 1.4 | -206.728103 | 8.0 | -207.073716 | 19.2 | -207.078972 | 30.4 | -207.077540 |
| 1.5 | -206.777119 | 8.2 | -207.073926 | 19.4 | -207.078894 | 30.6 | -207.077534 |
| 1.6 | -206.817559 | 8.4 | -207.074113 | 19.6 | -207.078820 | 30.8 | -207.077529 |
| 1.7 | -206.851129 | 8.6 | -207.074289 | 19.8 | -207.078750 | 31.0 | -207.077523 |
| 1.8 | -206.878883 | 8.8 | -207.074463 | 20.0 | -207.078683 | 31.2 | -207.077518 |
| 1.9 | -206.901642 | 9.0 | -207.074643 | 20.2 | -207.078621 | 31.4 | -207.077514 |
| 2.0 | -206.922213 | 9.2 | -207.074834 | 20.4 | -207.078561 | 31.6 | -207.077509 |
| 2.1 | -206.938320 | 9.4 | -207.075037 | 20.6 | -207.078505 | 31.8 | -207.077504 |
| 2.2 | -206.951550 | 9.6 | -207.075255 | 20.8 | -207.078452 | 32.0 | -207.077500 |
| 2.3 | -206.963493 | 9.8 | -207.075488 | 21.0 | -207.078402 | 33.0 | -207.077480 |
| 2.4 | -206.974345 | 10.0 | -207.075736 | 21.2 | -207.078355 | 34.0 | -207.077463 |
| 2.5 | -206.983878 | 10.2 | -207.075998 | 21.4 | -207.078310 | 35.0 | -207.077448 |
| 2.6 | -206.992049 | 10.4 | -207.076271 | 21.6 | -207.078268 | 36.0 | -207.077436 |
| 2.7 | -206.998984 | 10.6 | -207.076553 | 21.8 | -207.078228 | 37.0 | -207.077425 |
| 2.8 | -207.004860 | 10.8 | -207.076841 | 22.0 | -207.078190 | 38.0 | -207.077415 |
| 2.9 | -207.009860 | 11.0 | -207.077132 | 22.2 | -207.078155 | 39.0 | -207.077407 |
| 3.0 | -207.014148 | 11.2 | -207.077423 | 22.4 | -207.078121 | 40.0 | -207.077400 |
| 3.1 | -207.017867 | 11.4 | -207.077711 | 22.6 | -207.078089 | 42.0 | -207.077388 |
| 3.2 | -207.021136 | 11.6 | -207.077991 | 22.8 | -207.078059 | 44.0 | -207.077379 |
| 3.3 | -207.024059 | 11.8 | -207.078265 | 23.0 | -207.078031 | 46.0 | -207.077371 |
| 3.4 | -207.026720 | 12.0 | -207.078529 | 23.2 | -207.078003 | 48.0 | -207.077365 |
| 3.5 | -207.029192 | 12.2 | -207.078781 | 23.4 | -207.077978 | 50.0 | -207.077361 |
| 3.6 | -207.031529 | 12.4 | -207.079020 | 23.6 | -207.077953 | 52.0 | -207.077357 |
| 3.7 | -207.033773 | 12.6 | -207.079245 | 23.8 | -207.077930 | 54.0 | -207.077354 |
| 3.8 | -207.035951 | 12.8 | -207.079453 | 24.0 | -207.077908 | 56.0 | -207.077351 |
| 3.9 | -207.038075 | 13.0 | -207.079645 | 24.2 | -207.077887 | 58.0 | -207.077349 |
| 4.0 | -207.040145 | 13.2 | -207.079820 | 24.4 | -207.077867 | 60.0 | -207.077347 |
| 4.1 | -207.042152 | 13.4 | -207.079978 | 24.6 | -207.077848 | 62.0 | -207.077345 |
| 4.2 | -207.044083 | 13.6 | -207.080117 | 24.8 | -207.077830 | 64.0 | -207.077344 |
| 4.3 | -207.045920 | 13.8 | -207.080238 | 25.0 | -207.077812 | 66.0 | -207.077343 |
| 4.4 | -207.047652 | 14.0 | -207.080341 | 25.2 | -207.077796 | 68.0 | -207.077342 |
| 4.5 | -207.049270 | 14.2 | -207.080425 | 25.4 | -207.077780 | 70.0 | -207.077341 |
| 4.6 | -207.050768 | 14.4 | -207.080491 | 25.6 | -207.077765 | 72.0 | -207.077340 |
| 4.7 | -207.052151 | 14.6 | -207.080539 | 25.8 | -207.077750 | 74.0 | -207.077340 |
| 4.8 | -207.053424 | 14.8 | -207.080570 | 26.0 | -207.077736 | 76.0 | -207.077339 |
| 4.9 | -207.054600 | 15.0 | -207.080583 | 26.2 | -207.077723 | 78.0 | -207.077339 |
| 5.0 | -207.055692 | 15.2 | -207.080580 | 26.4 | -207.077710 | 80.0 | -207.077338 |
| 5.1 | -207.056718 | 15.4 | -207.080562 | 26.6 | -207.077698 | 82.0 | -207.077338 |
| 5.2 | -207.057692 | 15.6 | -207.080529 | 26.8 | -207.077687 | 84.0 | -207.077337 |
| 5.3 | -207.058631 | 15.8 | -207.080483 | 27.0 | -207.077675 | 86.0 | -207.077337 |
| 5.4 | -207.059547 | 16.0 | -207.080426 | 27.2 | -207.077665 | 88.0 | -207.077337 |
| 5.5 | -207.060450 | 16.2 | -207.080358 | 27.4 | -207.077654 | 90.0 | -207.077337 |
| 5.6 | -207.061345 | 16.4 | -207.080280 | 27.6 | -207.077645 | 92.0 | -207.077337 |
| 5.7 | -207.062237 | 16.6 | -207.080196 | 27.8 | -207.077635 | 94.0 | -207.077336 |
| 5.8 | -207.063125 | 16.8 | -207.080105 | 28.0 | -207.077626 | 96.0 | -207.077336 |
| 5.9 | -207.064006 | 17.0 | -207.080010 | 28.2 | -207.077617 | 98.0 | -207.077336 |
| 6.0 | -207.064874 | 17.2 | -207.079912 | 28.4 | -207.077609 | 100.0 | -207.077336 |
| 6.2 | -207.066547 | 17.4 | -207.079812 | 28.6 | -207.077601 | 150.0 | -207.077335 |
| 6.4 | -207.068090 | 17.6 | -207.079711 | 28.8 | -207.077593 | 200.0 | -207.077334 |
| 6.6 | -207.069450 | 17.8 | -207.079611 | 29.0 | -207.077585 | | |
| 6.8 | -207.070591 | 18.0 | -207.079511 | 29.2 | -207.077578 | | |
| 7.0 | -207.071505 | 18.2 | -207.079414 | 29.4 | -207.077571 | | |
| 7.2 | -207.072211 | 18.4 | -207.079319 | 29.6 | -207.077564 | | |
| 7.4 | -207.072746 | 18.6 | -207.079227 | 29.8 | -207.077558 | | |
| 7.6 | -207.073152 | 18.8 | -207.079138 | 30.0 | -207.077552 | | |
| 7.8 | -207.073465 | 19.0 | -207.079053 | 30.2 | -207.077546 | | |

Table S7: Total IH-FS-CCSD (2,0)/unANO-RCC+ energies for the $1^3\Sigma^+$ state of $LiMg^+$.

| R (Å) | E (a.u.) | R (Å) | E (a.u.) | R (Å) | E (a.u.) | R (Å) | E (a.u.) |
|---|---|---|---|---|---|---|---|
| 1.4 | -206.927881 | 8.0 | -207.203512 | 19.2 | -207.201252 | 30.4 | -207.201212 |
| 1.5 | -206.975890 | 8.2 | -207.203200 | 19.4 | -207.201250 | 30.6 | -207.201211 |
| 1.6 | -207.017805 | 8.4 | -207.202943 | 19.6 | -207.201248 | 30.8 | -207.201211 |
| 1.7 | -207.054947 | 8.6 | -207.202731 | 19.8 | -207.201247 | 31.0 | -207.201211 |
| 1.8 | -207.087508 | 8.8 | -207.202555 | 20.0 | -207.201245 | 31.2 | -207.201211 |
| 1.9 | -207.115514 | 9.0 | -207.202406 | 20.2 | -207.201243 | 31.4 | -207.201211 |
| 2.0 | -207.139185 | 9.2 | -207.202281 | 20.4 | -207.201242 | 31.6 | -207.201210 |
| 2.1 | -207.158931 | 9.4 | -207.202174 | 20.6 | -207.201240 | 31.8 | -207.201210 |
| 2.2 | -207.175246 | 9.6 | -207.202082 | 20.8 | -207.201239 | 32.0 | -207.201210 |
| 2.3 | -207.188626 | 9.8 | -207.202001 | 21.0 | -207.201238 | 33.0 | -207.201209 |
| 2.4 | -207.199532 | 10.0 | -207.201931 | 21.2 | -207.201236 | 34.0 | -207.201209 |
| 2.5 | -207.208369 | 10.2 | -207.201869 | 21.4 | -207.201235 | 35.0 | -207.201208 |
| 2.6 | -207.215484 | 10.4 | -207.201814 | 21.6 | -207.201234 | 36.0 | -207.201208 |
| 2.7 | -207.221167 | 10.6 | -207.201765 | 21.8 | -207.201233 | 37.0 | -207.201207 |
| 2.8 | -207.225659 | 10.8 | -207.201722 | 22.0 | -207.201232 | 38.0 | -207.201207 |
| 2.9 | -207.229158 | 11.0 | -207.201682 | 22.2 | -207.201231 | 39.0 | -207.201207 |
| 3.0 | -207.231832 | 11.2 | -207.201647 | 22.4 | -207.201230 | 40.0 | -207.201206 |
| 3.1 | -207.233821 | 11.4 | -207.201615 | 22.6 | -207.201229 | 42.0 | -207.201206 |
| 3.2 | -207.235237 | 11.6 | -207.201586 | 22.8 | -207.201228 | 44.0 | -207.201206 |
| 3.3 | -207.236176 | 11.8 | -207.201559 | 23.0 | -207.201227 | 46.0 | -207.201205 |
| 3.4 | -207.236716 | 12.0 | -207.201535 | 23.2 | -207.201226 | 48.0 | -207.201205 |
| 3.5 | -207.236923 | 12.2 | -207.201513 | 23.4 | -207.201226 | 50.0 | -207.201205 |
| 3.6 | -207.236854 | 12.4 | -207.201493 | 23.6 | -207.201225 | 52.0 | -207.201205 |
| 3.7 | -207.236553 | 12.6 | -207.201474 | 23.8 | -207.201224 | 54.0 | -207.201205 |
| 3.8 | -207.236061 | 12.8 | -207.201457 | 24.0 | -207.201224 | 56.0 | -207.201205 |
| 3.9 | -207.235411 | 13.0 | -207.201441 | 24.2 | -207.201223 | 58.0 | -207.201204 |
| 4.0 | -207.234631 | 13.2 | -207.201427 | 24.4 | -207.201222 | 60.0 | -207.201204 |
| 4.1 | -207.233745 | 13.4 | -207.201413 | 24.6 | -207.201222 | 62.0 | -207.201204 |
| 4.2 | -207.232773 | 13.6 | -207.201401 | 24.8 | -207.201221 | 64.0 | -207.201204 |
| 4.3 | -207.231734 | 13.8 | -207.201390 | 25.0 | -207.201221 | 66.0 | -207.201204 |
| 4.4 | -207.230643 | 14.0 | -207.201379 | 25.2 | -207.201220 | 68.0 | -207.201204 |
| 4.5 | -207.229512 | 14.2 | -207.201369 | 25.4 | -207.201220 | 70.0 | -207.201204 |
| 4.6 | -207.228354 | 14.4 | -207.201360 | 25.6 | -207.201219 | 72.0 | -207.201204 |
| 4.7 | -207.227177 | 14.6 | -207.201351 | 25.8 | -207.201219 | 74.0 | -207.201204 |
| 4.8 | -207.225991 | 14.8 | -207.201343 | 26.0 | -207.201218 | 76.0 | -207.201204 |
| 4.9 | -207.224804 | 15.0 | -207.201336 | 26.2 | -207.201218 | 78.0 | -207.201204 |
| 5.0 | -207.223621 | 15.2 | -207.201329 | 26.4 | -207.201217 | 80.0 | -207.201204 |
| 5.1 | -207.222449 | 15.4 | -207.201322 | 26.6 | -207.201217 | 82.0 | -207.201204 |
| 5.2 | -207.221293 | 15.6 | -207.201316 | 26.8 | -207.201217 | 84.0 | -207.201204 |
| 5.3 | -207.220158 | 15.8 | -207.201311 | 27.0 | -207.201216 | 86.0 | -207.201204 |
| 5.4 | -207.219047 | 16.0 | -207.201305 | 27.2 | -207.201216 | 88.0 | -207.201204 |
| 5.5 | -207.217965 | 16.2 | -207.201300 | 27.4 | -207.201215 | 90.0 | -207.201204 |
| 5.6 | -207.216914 | 16.4 | -207.201296 | 27.6 | -207.201215 | 92.0 | -207.201204 |
| 5.7 | -207.215898 | 16.6 | -207.201291 | 27.8 | -207.201215 | 94.0 | -207.201204 |
| 5.8 | -207.214919 | 16.8 | -207.201287 | 28.0 | -207.201215 | 96.0 | -207.201204 |
| 5.9 | -207.213979 | 17.0 | -207.201283 | 28.2 | -207.201214 | 98.0 | -207.201204 |
| 6.0 | -207.213079 | 17.2 | -207.201279 | 28.4 | -207.201214 | 100.0 | -207.201204 |
| 6.2 | -207.211406 | 17.4 | -207.201276 | 28.6 | -207.201214 | 150.0 | -207.201204 |
| 6.4 | -207.209908 | 17.6 | -207.201273 | 28.8 | -207.201213 | 200.0 | -207.201204 |
| 6.6 | -207.208586 | 17.8 | -207.201270 | 29.0 | -207.201213 | | |
| 6.8 | -207.207437 | 18.0 | -207.201267 | 29.2 | -207.201213 | | |
| 7.0 | -207.206453 | 18.2 | -207.201264 | 29.4 | -207.201213 | | |
| 7.2 | -207.205624 | 18.4 | -207.201261 | 29.6 | -207.201212 | | |
| 7.4 | -207.204932 | 18.6 | -207.201259 | 29.8 | -207.201212 | | |
| 7.6 | -207.204362 | 18.8 | -207.201257 | 30.0 | -207.201212 | | |
| 7.8 | -207.203895 | 19.0 | -207.201254 | 30.2 | -207.201212 | | |

Table S8: Total IH-FS-CCSD (2,0)/unANO-RCC+ energies for the $2^3\Sigma^+$ state of $LiMg^+$.

| R (Å) | E (a.u.) | R (Å) | E (a.u.) | R (Å) | E (a.u.) | R (Å) | E (a.u.) |
|---|---|---|---|---|---|---|---|
| 1.4 | -206.838899 | 8.0 | -207.188091 | 19.2 | -207.184827 | 30.4 | -207.184665 |
| 1.5 | -206.896836 | 8.2 | -207.187931 | 19.4 | -207.184821 | 30.6 | -207.184664 |
| 1.6 | -206.943510 | 8.4 | -207.187753 | 19.6 | -207.184814 | 30.8 | -207.184663 |
| 1.7 | -206.980731 | 8.6 | -207.187569 | 19.8 | -207.184808 | 31.0 | -207.184662 |
| 1.8 | -207.010363 | 8.8 | -207.187385 | 20.0 | -207.184802 | 31.2 | -207.184661 |
| 1.9 | -207.034084 | 9.0 | -207.187201 | 20.2 | -207.184796 | 31.4 | -207.184660 |
| 2.0 | -207.053198 | 9.2 | -207.187031 | 20.4 | -207.184790 | 31.6 | -207.184659 |
| 2.1 | -207.068683 | 9.4 | -207.186872 | 20.6 | -207.184785 | 31.8 | -207.184658 |
| 2.2 | -207.081289 | 9.6 | -207.186722 | 20.8 | -207.184780 | 32.0 | -207.184657 |
| 2.3 | -207.091612 | 9.8 | -207.186583 | 21.0 | -207.184775 | 33.0 | -207.184653 |
| 2.4 | -207.100131 | 10.0 | -207.186455 | 21.2 | -207.184770 | 34.0 | -207.184650 |
| 2.5 | -207.107235 | 10.2 | -207.186337 | 21.4 | -207.184766 | 35.0 | -207.184647 |
| 2.6 | -207.113234 | 10.4 | -207.186227 | 21.6 | -207.184761 | 36.0 | -207.184644 |
| 2.7 | -207.118378 | 10.6 | -207.186127 | 21.8 | -207.184757 | 37.0 | -207.184642 |
| 2.8 | -207.122862 | 10.8 | -207.186034 | 22.0 | -207.184753 | 38.0 | -207.184640 |
| 2.9 | -207.126841 | 11.0 | -207.185948 | 22.2 | -207.184749 | 39.0 | -207.184638 |
| 3.0 | -207.130428 | 11.2 | -207.185869 | 22.4 | -207.184745 | 40.0 | -207.184636 |
| 3.1 | -207.133719 | 11.4 | -207.185796 | 22.6 | -207.184742 | 42.0 | -207.184633 |
| 3.2 | -207.136781 | 11.6 | -207.185724 | 22.8 | -207.184738 | 44.0 | -207.184631 |
| 3.3 | -207.139666 | 11.8 | -207.185661 | 23.0 | -207.184735 | 46.0 | -207.184629 |
| 3.4 | -207.142410 | 12.0 | -207.185603 | 23.2 | -207.184731 | 48.0 | -207.184627 |
| 3.5 | -207.145041 | 12.2 | -207.185550 | 23.4 | -207.184728 | 50.0 | -207.184626 |
| 3.6 | -207.147577 | 12.4 | -207.185500 | 23.6 | -207.184725 | 52.0 | -207.184624 |
| 3.7 | -207.150031 | 12.6 | -207.185453 | 23.8 | -207.184722 | 54.0 | -207.184623 |
| 3.8 | -207.152408 | 12.8 | -207.185410 | 24.0 | -207.184720 | 56.0 | -207.184622 |
| 3.9 | -207.154714 | 13.0 | -207.185370 | 24.2 | -207.184717 | 58.0 | -207.184622 |
| 4.0 | -207.156950 | 13.2 | -207.185333 | 24.4 | -207.184714 | 60.0 | -207.184621 |
| 4.1 | -207.159114 | 13.4 | -207.185298 | 24.6 | -207.184712 | 62.0 | -207.184620 |
| 4.2 | -207.161204 | 13.6 | -207.185265 | 24.8 | -207.184709 | 64.0 | -207.184620 |
| 4.3 | -207.163219 | 13.8 | -207.185235 | 25.0 | -207.184707 | 66.0 | -207.184619 |
| 4.4 | -207.165154 | 14.0 | -207.185206 | 25.2 | -207.184705 | 68.0 | -207.184619 |
| 4.5 | -207.167008 | 14.2 | -207.185179 | 25.4 | -207.184702 | 70.0 | -207.184619 |
| 4.6 | -207.168778 | 14.4 | -207.185154 | 25.6 | -207.184700 | 72.0 | -207.184618 |
| 4.7 | -207.170462 | 14.6 | -207.185130 | 25.8 | -207.184698 | 74.0 | -207.184618 |
| 4.8 | -207.172059 | 14.8 | -207.185107 | 26.0 | -207.184696 | 76.0 | -207.184618 |
| 4.9 | -207.173569 | 15.0 | -207.185086 | 26.2 | -207.184694 | 78.0 | -207.184618 |
| 5.0 | -207.174990 | 15.2 | -207.185066 | 26.4 | -207.184692 | 80.0 | -207.184617 |
| 5.1 | -207.176325 | 15.4 | -207.185048 | 26.6 | -207.184691 | 82.0 | -207.184617 |
| 5.2 | -207.177573 | 15.6 | -207.185030 | 26.8 | -207.184689 | 84.0 | -207.184617 |
| 5.3 | -207.178737 | 15.8 | -207.185013 | 27.0 | -207.184687 | 86.0 | -207.184617 |
| 5.4 | -207.179818 | 16.0 | -207.184997 | 27.2 | -207.184685 | 88.0 | -207.184617 |
| 5.5 | -207.180819 | 16.2 | -207.184982 | 27.4 | -207.184684 | 90.0 | -207.184617 |
| 5.6 | -207.181742 | 16.4 | -207.184968 | 27.6 | -207.184682 | 92.0 | -207.184617 |
| 5.7 | -207.182590 | 16.6 | -207.184954 | 27.8 | -207.184681 | 94.0 | -207.184616 |
| 5.8 | -207.183366 | 16.8 | -207.184941 | 28.0 | -207.184679 | 96.0 | -207.184616 |
| 5.9 | -207.184072 | 17.0 | -207.184929 | 28.2 | -207.184678 | 98.0 | -207.184616 |
| 6.0 | -207.184712 | 17.2 | -207.184917 | 28.4 | -207.184676 | 100.0 | -207.184616 |
| 6.2 | -207.185803 | 17.4 | -207.184906 | 28.6 | -207.184675 | 150.0 | -207.184615 |
| 6.4 | -207.186663 | 17.6 | -207.184896 | 28.8 | -207.184674 | 200.0 | -207.184615 |
| 6.6 | -207.187317 | 17.8 | -207.184886 | 29.0 | -207.184672 | | |
| 6.8 | -207.187786 | 18.0 | -207.184876 | 29.2 | -207.184671 | | |
| 7.0 | -207.188095 | 18.2 | -207.184867 | 29.4 | -207.184670 | | |
| 7.2 | -207.188269 | 18.4 | -207.184858 | 29.6 | -207.184669 | | |
| 7.4 | -207.188333 | 18.6 | -207.184850 | 29.8 | -207.184668 | | |
| 7.6 | -207.188310 | 18.8 | -207.184842 | 30.0 | -207.184667 | | |
| 7.8 | -207.188223 | 19.0 | -207.184835 | 30.2 | -207.184666 | | |

Table S9: Total IH-FS-CCSD (2,0)/unANO-RCC+ energies for the $3^3\Sigma^+$ state of $LiMg^+$.

| R (Å) | E (a.u.) | R (Å) | E (a.u.) | R (Å) | E (a.u.) | R (Å) | E (a.u.) |
|---|---|---|---|---|---|---|---|
| 1.4 | -206.762869 | 8.0 | -207.137094 | 19.2 | -207.133519 | 30.4 | -207.133312 |
| 1.5 | -206.812403 | 8.2 | -207.137120 | 19.4 | -207.133510 | 30.6 | -207.133311 |
| 1.6 | -206.853590 | 8.4 | -207.137088 | 19.6 | -207.133502 | 30.8 | -207.133309 |
| 1.7 | -206.888154 | 8.6 | -207.137010 | 19.8 | -207.133494 | 31.0 | -207.133308 |
| 1.8 | -206.917500 | 8.8 | -207.136896 | 20.0 | -207.133486 | 31.2 | -207.133307 |
| 1.9 | -206.943885 | 9.0 | -207.136756 | 20.2 | -207.133478 | 31.4 | -207.133306 |
| 2.0 | -206.965881 | 9.2 | -207.136598 | 20.4 | -207.133471 | 31.6 | -207.133305 |
| 2.1 | -206.984095 | 9.4 | -207.136429 | 20.6 | -207.133464 | 31.8 | -207.133304 |
| 2.2 | -206.999090 | 9.6 | -207.136254 | 20.8 | -207.133458 | 32.0 | -207.133303 |
| 2.3 | -207.011377 | 9.8 | -207.136079 | 21.0 | -207.133451 | 33.0 | -207.133298 |
| 2.4 | -207.021408 | 10.0 | -207.135906 | 21.2 | -207.133445 | 34.0 | -207.133293 |
| 2.5 | -207.029578 | 10.2 | -207.135738 | 21.4 | -207.133440 | 35.0 | -207.133289 |
| 2.6 | -207.036226 | 10.4 | -207.135578 | 21.6 | -207.133434 | 36.0 | -207.133286 |
| 2.7 | -207.041640 | 10.6 | -207.135426 | 21.8 | -207.133429 | 37.0 | -207.133283 |
| 2.8 | -207.046060 | 10.8 | -207.135283 | 22.0 | -207.133424 | 38.0 | -207.133280 |
| 2.9 | -207.049690 | 11.0 | -207.135150 | 22.2 | -207.133419 | 39.0 | -207.133278 |
| 3.0 | -207.052708 | 11.2 | -207.135025 | 22.4 | -207.133414 | 40.0 | -207.133276 |
| 3.1 | -207.055272 | 11.4 | -207.134910 | 22.6 | -207.133409 | 42.0 | -207.133272 |
| 3.2 | -207.057578 | 11.6 | -207.134804 | 22.8 | -207.133405 | 44.0 | -207.133269 |
| 3.3 | -207.060359 | 11.8 | -207.134707 | 23.0 | -207.133401 | 46.0 | -207.133266 |
| 3.4 | -207.064706 | 12.0 | -207.134617 | 23.2 | -207.133396 | 48.0 | -207.133264 |
| 3.5 | -207.069376 | 12.2 | -207.134534 | 23.4 | -207.133392 | 50.0 | -207.133262 |
| 3.6 | -207.073951 | 12.4 | -207.134458 | 23.6 | -207.133389 | 52.0 | -207.133261 |
| 3.7 | -207.078365 | 12.6 | -207.134388 | 23.8 | -207.133385 | 54.0 | -207.133259 |
| 3.8 | -207.082592 | 12.8 | -207.134324 | 24.0 | -207.133381 | 56.0 | -207.133258 |
| 3.9 | -207.086618 | 13.0 | -207.134264 | 24.2 | -207.133378 | 58.0 | -207.133257 |
| 4.0 | -207.090431 | 13.2 | -207.134209 | 24.4 | -207.133375 | 60.0 | -207.133256 |
| 4.1 | -207.094028 | 13.4 | -207.134159 | 24.6 | -207.133371 | 62.0 | -207.133256 |
| 4.2 | -207.097407 | 13.6 | -207.134112 | 24.8 | -207.133368 | 64.0 | -207.133255 |
| 4.3 | -207.100570 | 13.8 | -207.134068 | 25.0 | -207.133365 | 66.0 | -207.133254 |
| 4.4 | -207.103522 | 14.0 | -207.134027 | 25.2 | -207.133362 | 68.0 | -207.133254 |
| 4.5 | -207.106273 | 14.2 | -207.133989 | 25.4 | -207.133360 | 70.0 | -207.133253 |
| 4.6 | -207.108832 | 14.4 | -207.133954 | 25.6 | -207.133357 | 72.0 | -207.133253 |
| 4.7 | -207.111209 | 14.6 | -207.133921 | 25.8 | -207.133354 | 74.0 | -207.133253 |
| 4.8 | -207.113415 | 14.8 | -207.133890 | 26.0 | -207.133352 | 76.0 | -207.133252 |
| 4.9 | -207.115461 | 15.0 | -207.133861 | 26.2 | -207.133349 | 78.0 | -207.133252 |
| 5.0 | -207.117359 | 15.2 | -207.133834 | 26.4 | -207.133347 | 80.0 | -207.133252 |
| 5.1 | -207.119118 | 15.4 | -207.133809 | 26.6 | -207.133345 | 82.0 | -207.133252 |
| 5.2 | -207.120748 | 15.6 | -207.133785 | 26.8 | -207.133342 | 84.0 | -207.133251 |
| 5.3 | -207.122259 | 15.8 | -207.133762 | 27.0 | -207.133340 | 86.0 | -207.133251 |
| 5.4 | -207.123658 | 16.0 | -207.133741 | 27.2 | -207.133338 | 88.0 | -207.133251 |
| 5.5 | -207.124954 | 16.2 | -207.133721 | 27.4 | -207.133336 | 90.0 | -207.133251 |
| 5.6 | -207.126153 | 16.4 | -207.133702 | 27.6 | -207.133334 | 92.0 | -207.133251 |
| 5.7 | -207.127262 | 16.6 | -207.133684 | 27.8 | -207.133332 | 94.0 | -207.133251 |
| 5.8 | -207.128287 | 16.8 | -207.133667 | 28.0 | -207.133330 | 96.0 | -207.133250 |
| 5.9 | -207.129233 | 17.0 | -207.133651 | 28.2 | -207.133329 | 98.0 | -207.133250 |
| 6.0 | -207.130104 | 17.2 | -207.133636 | 28.4 | -207.133327 | 100.0 | -207.133250 |
| 6.2 | -207.131641 | 17.4 | -207.133621 | 28.6 | -207.133325 | 150.0 | -207.133249 |
| 6.4 | -207.132931 | 17.6 | -207.133608 | 28.8 | -207.133324 | 200.0 | -207.133249 |
| 6.6 | -207.134000 | 17.8 | -207.133595 | 29.0 | -207.133322 | | |
| 6.8 | -207.134873 | 18.0 | -207.133582 | 29.2 | -207.133320 | | |
| 7.0 | -207.135571 | 18.2 | -207.133570 | 29.4 | -207.133319 | | |
| 7.2 | -207.136115 | 18.4 | -207.133559 | 29.6 | -207.133317 | | |
| 7.4 | -207.136522 | 18.6 | -207.133548 | 29.8 | -207.133316 | | |
| 7.6 | -207.136809 | 18.8 | -207.133538 | 30.0 | -207.133315 | | |
| 7.8 | -207.136995 | 19.0 | -207.133528 | 30.2 | -207.133313 | | |

Table S10: Total IH-FS-CCSD (2,0)/unANO-RCC+ energies for the $4^3\Sigma^+$ state of $LiMg^+$.

| R (Å) | E (a.u.) | R (Å) | E (a.u.) | R (Å) | E (a.u.) | R (Å) | E (a.u.) |
|---|---|---|---|---|---|---|---|
| 1.4 | -206.710397 | 8.0 | -207.097411 | 19.2 | -207.096838 | 30.4 | -207.096416 |
| 1.5 | -206.761501 | 8.2 | -207.098352 | 19.4 | -207.096816 | 30.6 | -207.096414 |
| 1.6 | -206.807281 | 8.4 | -207.099191 | 19.6 | -207.096794 | 30.8 | -207.096412 |
| 1.7 | -206.847067 | 8.6 | -207.099927 | 19.8 | -207.096774 | 31.0 | -207.096410 |
| 1.8 | -206.881121 | 8.8 | -207.100561 | 20.0 | -207.096756 | 31.2 | -207.096409 |
| 1.9 | -206.909891 | 9.0 | -207.101092 | 20.2 | -207.096738 | 31.4 | -207.096407 |
| 2.0 | -206.934010 | 9.2 | -207.101530 | 20.4 | -207.096721 | 31.6 | -207.096405 |
| 2.1 | -206.954161 | 9.4 | -207.101877 | 20.6 | -207.096705 | 31.8 | -207.096404 |
| 2.2 | -206.971000 | 9.6 | -207.102137 | 20.8 | -207.096690 | 32.0 | -207.096402 |
| 2.3 | -206.985122 | 9.8 | -207.102318 | 21.0 | -207.096676 | 33.0 | -207.096396 |
| 2.4 | -206.997050 | 10.0 | -207.102426 | 21.2 | -207.096663 | 34.0 | -207.096390 |
| 2.5 | -207.007236 | 10.2 | -207.102468 | 21.4 | -207.096650 | 35.0 | -207.096385 |
| 2.6 | -207.016059 | 10.4 | -207.102451 | 21.6 | -207.096638 | 36.0 | -207.096381 |
| 2.7 | -207.023834 | 10.6 | -207.102381 | 21.8 | -207.096627 | 37.0 | -207.096377 |
| 2.8 | -207.030813 | 10.8 | -207.102266 | 22.0 | -207.096616 | 38.0 | -207.096374 |
| 2.9 | -207.037192 | 11.0 | -207.102113 | 22.2 | -207.096605 | 39.0 | -207.096371 |
| 3.0 | -207.043117 | 11.2 | -207.101928 | 22.4 | -207.096596 | 40.0 | -207.096369 |
| 3.1 | -207.048684 | 11.4 | -207.101717 | 22.6 | -207.096586 | 42.0 | -207.096365 |
| 3.2 | -207.053881 | 11.6 | -207.101485 | 22.8 | -207.096577 | 44.0 | -207.096362 |
| 3.3 | -207.058113 | 11.8 | -207.101241 | 23.0 | -207.096569 | 46.0 | -207.096359 |
| 3.4 | -207.060384 | 12.0 | -207.100988 | 23.2 | -207.096561 | 48.0 | -207.096357 |
| 3.5 | -207.061999 | 12.2 | -207.100730 | 23.4 | -207.096553 | 50.0 | -207.096355 |
| 3.6 | -207.063415 | 12.4 | -207.100473 | 23.6 | -207.096546 | 52.0 | -207.096354 |
| 3.7 | -207.064720 | 12.6 | -207.100219 | 23.8 | -207.096539 | 54.0 | -207.096353 |
| 3.8 | -207.065949 | 12.8 | -207.099972 | 24.0 | -207.096532 | 56.0 | -207.096352 |
| 3.9 | -207.067119 | 13.0 | -207.099733 | 24.2 | -207.096526 | 58.0 | -207.096351 |
| 4.0 | -207.068242 | 13.2 | -207.099504 | 24.4 | -207.096520 | 60.0 | -207.096351 |
| 4.1 | -207.069323 | 13.4 | -207.099288 | 24.6 | -207.096514 | 62.0 | -207.096350 |
| 4.2 | -207.070364 | 13.6 | -207.099083 | 24.8 | -207.096508 | 64.0 | -207.096350 |
| 4.3 | -207.071369 | 13.8 | -207.098892 | 25.0 | -207.096503 | 66.0 | -207.096349 |
| 4.4 | -207.072336 | 14.0 | -207.098713 | 25.2 | -207.096497 | 68.0 | -207.096349 |
| 4.5 | -207.073268 | 14.2 | -207.098548 | 25.4 | -207.096493 | 70.0 | -207.096349 |
| 4.6 | -207.074166 | 14.4 | -207.098394 | 25.6 | -207.096488 | 72.0 | -207.096348 |
| 4.7 | -207.075030 | 14.6 | -207.098252 | 25.8 | -207.096483 | 74.0 | -207.096348 |
| 4.8 | -207.075864 | 14.8 | -207.098122 | 26.0 | -207.096479 | 76.0 | -207.096348 |
| 4.9 | -207.076668 | 15.0 | -207.098001 | 26.2 | -207.096475 | 78.0 | -207.096348 |
| 5.0 | -207.077447 | 15.2 | -207.097891 | 26.4 | -207.096471 | 80.0 | -207.096348 |
| 5.1 | -207.078202 | 15.4 | -207.097789 | 26.6 | -207.096467 | 82.0 | -207.096348 |
| 5.2 | -207.078938 | 15.6 | -207.097696 | 26.8 | -207.096463 | 84.0 | -207.096347 |
| 5.3 | -207.079658 | 15.8 | -207.097610 | 27.0 | -207.096460 | 86.0 | -207.096347 |
| 5.4 | -207.080365 | 16.0 | -207.097531 | 27.2 | -207.096456 | 88.0 | -207.096347 |
| 5.5 | -207.081062 | 16.2 | -207.097458 | 27.4 | -207.096453 | 90.0 | -207.096347 |
| 5.6 | -207.081752 | 16.4 | -207.097391 | 27.6 | -207.096450 | 92.0 | -207.096347 |
| 5.7 | -207.082439 | 16.6 | -207.097330 | 27.8 | -207.096447 | 94.0 | -207.096347 |
| 5.8 | -207.083124 | 16.8 | -207.097273 | 28.0 | -207.096444 | 96.0 | -207.096347 |
| 5.9 | -207.083810 | 17.0 | -207.097220 | 28.2 | -207.096441 | 98.0 | -207.096347 |
| 6.0 | -207.084497 | 17.2 | -207.097171 | 28.4 | -207.096438 | 100.0 | -207.096347 |
| 6.2 | -207.085878 | 17.4 | -207.097126 | 28.6 | -207.096435 | 150.0 | -207.096346 |
| 6.4 | -207.087270 | 17.6 | -207.097085 | 28.8 | -207.096433 | 200.0 | -207.096346 |
| 6.6 | -207.088664 | 17.8 | -207.097046 | 29.0 | -207.096430 | | |
| 6.8 | -207.090053 | 18.0 | -207.097010 | 29.2 | -207.096428 | | |
| 7.0 | -207.091421 | 18.2 | -207.096976 | 29.4 | -207.096426 | | |
| 7.2 | -207.092752 | 18.4 | -207.096945 | 29.6 | -207.096424 | | |
| 7.4 | -207.094031 | 18.6 | -207.096915 | 29.8 | -207.096422 | | |
| 7.6 | -207.095242 | 18.8 | -207.096888 | 30.0 | -207.096419 | | |
| 7.8 | -207.096372 | 19.0 | -207.096862 | 30.2 | -207.096418 | | |

Table S11: Total IH-FS-CCSD (2,0)/unANO-RCC+ energies for the $5^3\Sigma^+$ state of $LiMg^+$.

| R (Å) | E (a.u.) | R (Å) | E (a.u.) | R (Å) | E (a.u.) | R (Å) | E (a.u.) |
|---|---|---|---|---|---|---|---|
| 1.4 | -206.708963 | 8.0 | -207.069937 | 19.2 | -207.079057 | 30.4 | -207.077540 |
| 1.5 | -206.751350 | 8.2 | -207.071260 | 19.4 | -207.078964 | 30.6 | -207.077534 |
| 1.6 | -206.790944 | 8.4 | -207.072481 | 19.6 | -207.078878 | 30.8 | -207.077529 |
| 1.7 | -206.827380 | 8.6 | -207.073607 | 19.8 | -207.078797 | 31.0 | -207.077523 |
| 1.8 | -206.857790 | 8.8 | -207.074646 | 20.0 | -207.078722 | 31.2 | -207.077518 |
| 1.9 | -206.883059 | 9.0 | -207.075607 | 20.2 | -207.078652 | 31.4 | -207.077514 |
| 2.0 | -206.904017 | 9.2 | -207.076494 | 20.4 | -207.078587 | 31.6 | -207.077509 |
| 2.1 | -206.921380 | 9.4 | -207.077314 | 20.6 | -207.078526 | 31.8 | -207.077504 |
| 2.2 | -206.935775 | 9.6 | -207.078070 | 20.8 | -207.078469 | 32.0 | -207.077500 |
| 2.3 | -206.947781 | 9.8 | -207.078768 | 21.0 | -207.078416 | 33.0 | -207.077480 |
| 2.4 | -206.957986 | 10.0 | -207.079410 | 21.2 | -207.078366 | 34.0 | -207.077463 |
| 2.5 | -206.967092 | 10.2 | -207.079999 | 21.4 | -207.078319 | 35.0 | -207.077448 |
| 2.6 | -206.975411 | 10.4 | -207.080536 | 21.6 | -207.078275 | 36.0 | -207.077436 |
| 2.7 | -206.982771 | 10.6 | -207.081025 | 21.8 | -207.078234 | 37.0 | -207.077425 |
| 2.8 | -206.989180 | 10.8 | -207.081466 | 22.0 | -207.078195 | 38.0 | -207.077415 |
| 2.9 | -206.994748 | 11.0 | -207.081860 | 22.2 | -207.078159 | 39.0 | -207.077407 |
| 3.0 | -206.999598 | 11.2 | -207.082210 | 22.4 | -207.078124 | 40.0 | -207.077400 |
| 3.1 | -207.003844 | 11.4 | -207.082517 | 22.6 | -207.078092 | 42.0 | -207.077388 |
| 3.2 | -207.007593 | 11.6 | -207.082781 | 22.8 | -207.078061 | 44.0 | -207.077379 |
| 3.3 | -207.010942 | 11.8 | -207.083005 | 23.0 | -207.078032 | 46.0 | -207.077371 |
| 3.4 | -207.013982 | 12.0 | -207.083189 | 23.2 | -207.078005 | 48.0 | -207.077365 |
| 3.5 | -207.016798 | 12.2 | -207.083336 | 23.4 | -207.077979 | 50.0 | -207.077361 |
| 3.6 | -207.019474 | 12.4 | -207.083446 | 23.6 | -207.077954 | 52.0 | -207.077357 |
| 3.7 | -207.022087 | 12.6 | -207.083522 | 23.8 | -207.077931 | 54.0 | -207.077354 |
| 3.8 | -207.024669 | 12.8 | -207.083566 | 24.0 | -207.077908 | 56.0 | -207.077351 |
| 3.9 | -207.027182 | 13.0 | -207.083578 | 24.2 | -207.077887 | 58.0 | -207.077349 |
| 4.0 | -207.029550 | 13.2 | -207.083562 | 24.4 | -207.077867 | 60.0 | -207.077347 |
| 4.1 | -207.031721 | 13.4 | -207.083519 | 24.6 | -207.077848 | 62.0 | -207.077345 |
| 4.2 | -207.033677 | 13.6 | -207.083452 | 24.8 | -207.077830 | 64.0 | -207.077344 |
| 4.3 | -207.035420 | 13.8 | -207.083362 | 25.0 | -207.077812 | 66.0 | -207.077343 |
| 4.4 | -207.036967 | 14.0 | -207.083253 | 25.2 | -207.077796 | 68.0 | -207.077342 |
| 4.5 | -207.038347 | 14.2 | -207.083125 | 25.4 | -207.077780 | 70.0 | -207.077341 |
| 4.6 | -207.039597 | 14.4 | -207.082982 | 25.6 | -207.077765 | 72.0 | -207.077340 |
| 4.7 | -207.040767 | 14.6 | -207.082824 | 25.8 | -207.077750 | 74.0 | -207.077340 |
| 4.8 | -207.041916 | 14.8 | -207.082656 | 26.0 | -207.077736 | 76.0 | -207.077339 |
| 4.9 | -207.043090 | 15.0 | -207.082477 | 26.2 | -207.077723 | 78.0 | -207.077339 |
| 5.0 | -207.044298 | 15.2 | -207.082291 | 26.4 | -207.077710 | 80.0 | -207.077338 |
| 5.1 | -207.045522 | 15.4 | -207.082100 | 26.6 | -207.077698 | 82.0 | -207.077338 |
| 5.2 | -207.046738 | 15.6 | -207.081904 | 26.8 | -207.077687 | 84.0 | -207.077337 |
| 5.3 | -207.047929 | 15.8 | -207.081707 | 27.0 | -207.077675 | 86.0 | -207.077337 |
| 5.4 | -207.049086 | 16.0 | -207.081509 | 27.2 | -207.077665 | 88.0 | -207.077337 |
| 5.5 | -207.050203 | 16.2 | -207.081312 | 27.4 | -207.077654 | 90.0 | -207.077337 |
| 5.6 | -207.051275 | 16.4 | -207.081117 | 27.6 | -207.077645 | 92.0 | -207.077337 |
| 5.7 | -207.052303 | 16.6 | -207.080926 | 27.8 | -207.077635 | 94.0 | -207.077336 |
| 5.8 | -207.053284 | 16.8 | -207.080740 | 28.0 | -207.077626 | 96.0 | -207.077336 |
| 5.9 | -207.054220 | 17.0 | -207.080559 | 28.2 | -207.077617 | 98.0 | -207.077336 |
| 6.0 | -207.055111 | 17.2 | -207.080385 | 28.4 | -207.077609 | 100.0 | -207.077336 |
| 6.2 | -207.056762 | 17.4 | -207.080217 | 28.6 | -207.077601 | 150.0 | -207.077335 |
| 6.4 | -207.058253 | 17.6 | -207.080057 | 28.8 | -207.077593 | 200.0 | -207.077334 |
| 6.6 | -207.059611 | 17.8 | -207.079905 | 29.0 | -207.077585 | | |
| 6.8 | -207.060891 | 18.0 | -207.079760 | 29.2 | -207.077578 | | |
| 7.0 | -207.062207 | 18.2 | -207.079624 | 29.4 | -207.077571 | | |
| 7.2 | -207.063700 | 18.4 | -207.079495 | 29.6 | -207.077564 | | |
| 7.4 | -207.065335 | 18.6 | -207.079375 | 29.8 | -207.077558 | | |
| 7.6 | -207.066963 | 18.8 | -207.079261 | 30.0 | -207.077552 | | |
| 7.8 | -207.068504 | 19.0 | -207.079156 | 30.2 | -207.077546 | | |

Table S12: Total IH-FS-CCSD (2,0)/unANO-RCC+ energies for the $1^1\Pi$ state of $LiMg^+$.

| R (Å) | E (a.u.) | R (Å) | E (a.u.) | R (Å) | E (a.u.) | R (Å) | E (a.u.) |
|---|---|---|---|---|---|---|---|
| 1.4 | -206.921608 | 8.0 | -207.133260 | 19.2 | -207.133172 | 30.4 | -207.133226 |
| 1.5 | -206.962856 | 8.2 | -207.133178 | 19.4 | -207.133174 | 30.6 | -207.133226 |
| 1.6 | -206.996139 | 8.4 | -207.133115 | 19.6 | -207.133175 | 30.8 | -207.133227 |
| 1.7 | -207.023670 | 8.6 | -207.133067 | 19.8 | -207.133177 | 31.0 | -207.133227 |
| 1.8 | -207.046679 | 8.8 | -207.133031 | 20.0 | -207.133179 | 31.2 | -207.133227 |
| 1.9 | -207.065897 | 9.0 | -207.133006 | 20.2 | -207.133181 | 31.4 | -207.133228 |
| 2.0 | -207.081845 | 9.2 | -207.132988 | 20.4 | -207.133183 | 31.6 | -207.133228 |
| 2.1 | -207.094938 | 9.4 | -207.132976 | 20.6 | -207.133184 | 31.8 | -207.133228 |
| 2.2 | -207.105556 | 9.6 | -207.132970 | 20.8 | -207.133186 | 32.0 | -207.133229 |
| 2.3 | -207.114065 | 9.8 | -207.132967 | 21.0 | -207.133187 | 33.0 | -207.133230 |
| 2.4 | -207.120793 | 10.0 | -207.132966 | 21.2 | -207.133189 | 34.0 | -207.133232 |
| 2.5 | -207.126045 | 10.2 | -207.132968 | 21.4 | -207.133190 | 35.0 | -207.133233 |
| 2.6 | -207.130081 | 10.4 | -207.132972 | 21.6 | -207.133192 | 36.0 | -207.133234 |
| 2.7 | -207.133130 | 10.6 | -207.132977 | 21.8 | -207.133193 | 37.0 | -207.133235 |
| 2.8 | -207.135388 | 10.8 | -207.132982 | 22.0 | -207.133194 | 38.0 | -207.133236 |
| 2.9 | -207.137018 | 11.0 | -207.132988 | 22.2 | -207.133196 | 39.0 | -207.133237 |
| 3.0 | -207.138159 | 11.2 | -207.132995 | 22.4 | -207.133197 | 40.0 | -207.133238 |
| 3.1 | -207.138929 | 11.4 | -207.133002 | 22.6 | -207.133198 | 42.0 | -207.133239 |
| 3.2 | -207.139422 | 11.6 | -207.133009 | 22.8 | -207.133199 | 44.0 | -207.133240 |
| 3.3 | -207.139715 | 11.8 | -207.133016 | 23.0 | -207.133200 | 46.0 | -207.133241 |
| 3.4 | -207.139873 | 12.0 | -207.133023 | 23.2 | -207.133201 | 48.0 | -207.133242 |
| 3.5 | -207.139939 | 12.2 | -207.133030 | 23.4 | -207.133202 | 50.0 | -207.133243 |
| 3.6 | -207.139946 | 12.4 | -207.133037 | 23.6 | -207.133203 | 52.0 | -207.133243 |
| 3.7 | -207.139915 | 12.6 | -207.133043 | 23.8 | -207.133204 | 54.0 | -207.133244 |
| 3.8 | -207.139860 | 12.8 | -207.133050 | 24.0 | -207.133205 | 56.0 | -207.133244 |
| 3.9 | -207.139787 | 13.0 | -207.133056 | 24.2 | -207.133206 | 58.0 | -207.133245 |
| 4.0 | -207.139698 | 13.2 | -207.133062 | 24.4 | -207.133207 | 60.0 | -207.133245 |
| 4.1 | -207.139599 | 13.4 | -207.133068 | 24.6 | -207.133208 | 62.0 | -207.133245 |
| 4.2 | -207.139483 | 13.6 | -207.133073 | 24.8 | -207.133209 | 64.0 | -207.133246 |
| 4.3 | -207.139351 | 13.8 | -207.133079 | 25.0 | -207.133210 | 66.0 | -207.133246 |
| 4.4 | -207.139206 | 14.0 | -207.133084 | 25.2 | -207.133211 | 68.0 | -207.133246 |
| 4.5 | -207.139048 | 14.2 | -207.133089 | 25.4 | -207.133211 | 70.0 | -207.133246 |
| 4.6 | -207.138872 | 14.4 | -207.133094 | 25.6 | -207.133212 | 72.0 | -207.133247 |
| 4.7 | -207.138684 | 14.6 | -207.133099 | 25.8 | -207.133213 | 74.0 | -207.133247 |
| 4.8 | -207.138488 | 14.8 | -207.133104 | 26.0 | -207.133214 | 76.0 | -207.133247 |
| 4.9 | -207.138279 | 15.0 | -207.133108 | 26.2 | -207.133214 | 78.0 | -207.133247 |
| 5.0 | -207.138061 | 15.2 | -207.133112 | 26.4 | -207.133215 | 80.0 | -207.133247 |
| 5.1 | -207.137833 | 15.4 | -207.133116 | 26.6 | -207.133216 | 82.0 | -207.133247 |
| 5.2 | -207.137604 | 15.6 | -207.133120 | 26.8 | -207.133216 | 84.0 | -207.133247 |
| 5.3 | -207.137371 | 15.8 | -207.133124 | 27.0 | -207.133217 | 86.0 | -207.133247 |
| 5.4 | -207.137136 | 16.0 | -207.133128 | 27.2 | -207.133218 | 88.0 | -207.133247 |
| 5.5 | -207.136902 | 16.2 | -207.133131 | 27.4 | -207.133218 | 90.0 | -207.133247 |
| 5.6 | -207.136669 | 16.4 | -207.133135 | 27.6 | -207.133219 | 92.0 | -207.133248 |
| 5.7 | -207.136439 | 16.6 | -207.133138 | 27.8 | -207.133219 | 94.0 | -207.133248 |
| 5.8 | -207.136212 | 16.8 | -207.133141 | 28.0 | -207.133220 | 96.0 | -207.133248 |
| 5.9 | -207.135991 | 17.0 | -207.133144 | 28.2 | -207.133221 | 98.0 | -207.133248 |
| 6.0 | -207.135775 | 17.2 | -207.133147 | 28.4 | -207.133221 | 100.0 | -207.133248 |
| 6.2 | -207.135365 | 17.4 | -207.133150 | 28.6 | -207.133222 | 150.0 | -207.133248 |
| 6.4 | -207.134987 | 17.6 | -207.133153 | 28.8 | -207.133222 | 200.0 | -207.133248 |
| 6.6 | -207.134644 | 17.8 | -207.133155 | 29.0 | -207.133223 | | |
| 6.8 | -207.134340 | 18.0 | -207.133158 | 29.2 | -207.133223 | | |
| 7.0 | -207.134074 | 18.2 | -207.133160 | 29.4 | -207.133224 | | |
| 7.2 | -207.133846 | 18.4 | -207.133163 | 29.6 | -207.133224 | | |
| 7.4 | -207.133654 | 18.6 | -207.133165 | 29.8 | -207.133224 | | |
| 7.6 | -207.133494 | 18.8 | -207.133167 | 30.0 | -207.133225 | | |
| 7.8 | -207.133364 | 19.0 | -207.133169 | 30.2 | -207.133225 | | |

Table S13: Total IH-FS-CCSD (2,0)/unANO-RCC+ energies for the $2^1\Pi$ state of $LiMg^+$.

| R (Å) | E (a.u.) | R (Å) | E (a.u.) | R (Å) | E (a.u.) | R (Å) | E (a.u.) |
|---|---|---|---|---|---|---|---|
| 1.4 | -206.819661 | 8.0 | -207.123425 | 19.2 | -207.124111 | 30.4 | -207.124163 |
| 1.5 | -206.868022 | 8.2 | -207.123538 | 19.4 | -207.124113 | 30.6 | -207.124164 |
| 1.6 | -206.909460 | 8.4 | -207.123626 | 19.6 | -207.124115 | 30.8 | -207.124164 |
| 1.7 | -206.944904 | 8.6 | -207.123695 | 19.8 | -207.124117 | 31.0 | -207.124164 |
| 1.8 | -206.974897 | 8.8 | -207.123749 | 20.0 | -207.124118 | 31.2 | -207.124165 |
| 1.9 | -207.000031 | 9.0 | -207.123791 | 20.2 | -207.124120 | 31.4 | -207.124165 |
| 2.0 | -207.020964 | 9.2 | -207.123824 | 20.4 | -207.124122 | 31.6 | -207.124165 |
| 2.1 | -207.038336 | 9.4 | -207.123850 | 20.6 | -207.124123 | 31.8 | -207.124166 |
| 2.2 | -207.052721 | 9.6 | -207.123872 | 20.8 | -207.124125 | 32.0 | -207.124166 |
| 2.3 | -207.064635 | 9.8 | -207.123889 | 21.0 | -207.124126 | 33.0 | -207.124168 |
| 2.4 | -207.074496 | 10.0 | -207.123903 | 21.2 | -207.124127 | 34.0 | -207.124169 |
| 2.5 | -207.082666 | 10.2 | -207.123914 | 21.4 | -207.124129 | 35.0 | -207.124171 |
| 2.6 | -207.089432 | 10.4 | -207.123924 | 21.6 | -207.124130 | 36.0 | -207.124172 |
| 2.7 | -207.095028 | 10.6 | -207.123933 | 21.8 | -207.124131 | 37.0 | -207.124173 |
| 2.8 | -207.099647 | 10.8 | -207.123942 | 22.0 | -207.124133 | 38.0 | -207.124174 |
| 2.9 | -207.103438 | 11.0 | -207.123949 | 22.2 | -207.124134 | 39.0 | -207.124175 |
| 3.0 | -207.106525 | 11.2 | -207.123957 | 22.4 | -207.124135 | 40.0 | -207.124175 |
| 3.1 | -207.109012 | 11.4 | -207.123963 | 22.6 | -207.124136 | 42.0 | -207.124177 |
| 3.2 | -207.110980 | 11.6 | -207.123968 | 22.8 | -207.124137 | 44.0 | -207.124178 |
| 3.3 | -207.112500 | 11.8 | -207.123975 | 23.0 | -207.124138 | 46.0 | -207.124179 |
| 3.4 | -207.113634 | 12.0 | -207.123981 | 23.2 | -207.124139 | 48.0 | -207.124180 |
| 3.5 | -207.114441 | 12.2 | -207.123987 | 23.4 | -207.124140 | 50.0 | -207.124181 |
| 3.6 | -207.114976 | 12.4 | -207.123993 | 23.6 | -207.124141 | 52.0 | -207.124181 |
| 3.7 | -207.115291 | 12.6 | -207.123999 | 23.8 | -207.124142 | 54.0 | -207.124182 |
| 3.8 | -207.115437 | 12.8 | -207.124004 | 24.0 | -207.124143 | 56.0 | -207.124182 |
| 3.9 | -207.115461 | 13.0 | -207.124009 | 24.2 | -207.124144 | 58.0 | -207.124183 |
| 4.0 | -207.115405 | 13.2 | -207.124015 | 24.4 | -207.124145 | 60.0 | -207.124183 |
| 4.1 | -207.115306 | 13.4 | -207.124020 | 24.6 | -207.124146 | 62.0 | -207.124183 |
| 4.2 | -207.115198 | 13.6 | -207.124025 | 24.8 | -207.124147 | 64.0 | -207.124184 |
| 4.3 | -207.115107 | 13.8 | -207.124029 | 25.0 | -207.124148 | 66.0 | -207.124184 |
| 4.4 | -207.115051 | 14.0 | -207.124033 | 25.2 | -207.124148 | 68.0 | -207.124184 |
| 4.5 | -207.115045 | 14.2 | -207.124038 | 25.4 | -207.124149 | 70.0 | -207.124184 |
| 4.6 | -207.115096 | 14.4 | -207.124042 | 25.6 | -207.124150 | 72.0 | -207.124184 |
| 4.7 | -207.115208 | 14.6 | -207.124046 | 25.8 | -207.124151 | 74.0 | -207.124185 |
| 4.8 | -207.115379 | 14.8 | -207.124050 | 26.0 | -207.124151 | 76.0 | -207.124185 |
| 4.9 | -207.115604 | 15.0 | -207.124054 | 26.2 | -207.124152 | 78.0 | -207.124185 |
| 5.0 | -207.115877 | 15.2 | -207.124058 | 26.4 | -207.124153 | 80.0 | -207.124185 |
| 5.1 | -207.116190 | 15.4 | -207.124061 | 26.6 | -207.124153 | 82.0 | -207.124185 |
| 5.2 | -207.116534 | 15.6 | -207.124065 | 26.8 | -207.124154 | 84.0 | -207.124185 |
| 5.3 | -207.116902 | 15.8 | -207.124068 | 27.0 | -207.124155 | 86.0 | -207.124185 |
| 5.4 | -207.117285 | 16.0 | -207.124071 | 27.2 | -207.124155 | 88.0 | -207.124185 |
| 5.5 | -207.117677 | 16.2 | -207.124074 | 27.4 | -207.124156 | 90.0 | -207.124185 |
| 5.6 | -207.118071 | 16.4 | -207.124077 | 27.6 | -207.124156 | 92.0 | -207.124185 |
| 5.7 | -207.118462 | 16.6 | -207.124080 | 27.8 | -207.124157 | 94.0 | -207.124186 |
| 5.8 | -207.118847 | 16.8 | -207.124083 | 28.0 | -207.124157 | 96.0 | -207.124186 |
| 5.9 | -207.119222 | 17.0 | -207.124086 | 28.2 | -207.124158 | 98.0 | -207.124186 |
| 6.0 | -207.119585 | 17.2 | -207.124089 | 28.4 | -207.124159 | 100.0 | -207.124186 |
| 6.2 | -207.120264 | 17.4 | -207.124091 | 28.6 | -207.124159 | 150.0 | -207.124186 |
| 6.4 | -207.120875 | 17.6 | -207.124094 | 28.8 | -207.124160 | 200.0 | -207.124186 |
| 6.6 | -207.121413 | 17.8 | -207.124096 | 29.0 | -207.124160 | | |
| 6.8 | -207.121878 | 18.0 | -207.124099 | 29.2 | -207.124161 | | |
| 7.0 | -207.122274 | 18.2 | -207.124101 | 29.4 | -207.124161 | | |
| 7.2 | -207.122605 | 18.4 | -207.124103 | 29.6 | -207.124161 | | |
| 7.4 | -207.122879 | 18.6 | -207.124105 | 29.8 | -207.124162 | | |
| 7.6 | -207.123103 | 18.8 | -207.124107 | 30.0 | -207.124162 | | |
| 7.8 | -207.123282 | 19.0 | -207.124109 | 30.2 | -207.124163 | | |

Table S14: Total IH-FS-CCSD (2,0)/unANO-RCC+ energies for the $1^3\Pi$ state of $LiMg^+$.

| R (Å) | E (a.u.) | R (Å) | E (a.u.) | R (Å) | E (a.u.) | R (Å) | E (a.u.) |
|---|---|---|---|---|---|---|---|
| 1.4 | -206.999024 | 8.0 | -207.184202 | 19.2 | -207.184548 | 30.4 | -207.184596 |
| 1.5 | -207.040844 | 8.2 | -207.184211 | 19.4 | -207.184550 | 30.6 | -207.184596 |
| 1.6 | -207.074324 | 8.4 | -207.184222 | 19.6 | -207.184552 | 30.8 | -207.184596 |
| 1.7 | -207.101481 | 8.6 | -207.184233 | 19.8 | -207.184554 | 31.0 | -207.184597 |
| 1.8 | -207.123536 | 8.8 | -207.184245 | 20.0 | -207.184555 | 31.2 | -207.184597 |
| 1.9 | -207.141335 | 9.0 | -207.184257 | 20.2 | -207.184557 | 31.4 | -207.184597 |
| 2.0 | -207.155556 | 9.2 | -207.184269 | 20.4 | -207.184558 | 31.6 | -207.184598 |
| 2.1 | -207.166770 | 9.4 | -207.184281 | 20.6 | -207.184560 | 31.8 | -207.184598 |
| 2.2 | -207.175480 | 9.6 | -207.184295 | 20.8 | -207.184561 | 32.0 | -207.184598 |
| 2.3 | -207.182123 | 9.8 | -207.184307 | 21.0 | -207.184562 | 33.0 | -207.184600 |
| 2.4 | -207.187083 | 10.0 | -207.184319 | 21.2 | -207.184564 | 34.0 | -207.184601 |
| 2.5 | -207.190684 | 10.2 | -207.184329 | 21.4 | -207.184565 | 35.0 | -207.184602 |
| 2.6 | -207.193200 | 10.4 | -207.184340 | 21.6 | -207.184566 | 36.0 | -207.184603 |
| 2.7 | -207.194858 | 10.6 | -207.184351 | 21.8 | -207.184567 | 37.0 | -207.184604 |
| 2.8 | -207.195845 | 10.8 | -207.184361 | 22.0 | -207.184568 | 38.0 | -207.184605 |
| 2.9 | -207.196310 | 11.0 | -207.184371 | 22.2 | -207.184569 | 39.0 | -207.184606 |
| 3.0 | -207.196380 | 11.2 | -207.184380 | 22.4 | -207.184571 | 40.0 | -207.184606 |
| 3.1 | -207.196160 | 11.4 | -207.184389 | 22.6 | -207.184572 | 42.0 | -207.184607 |
| 3.2 | -207.195730 | 11.6 | -207.184391 | 22.8 | -207.184573 | 44.0 | -207.184608 |
| 3.3 | -207.195152 | 11.8 | -207.184399 | 23.0 | -207.184574 | 46.0 | -207.184609 |
| 3.4 | -207.194479 | 12.0 | -207.184407 | 23.2 | -207.184574 | 48.0 | -207.184610 |
| 3.5 | -207.193751 | 12.2 | -207.184415 | 23.4 | -207.184575 | 50.0 | -207.184610 |
| 3.6 | -207.192998 | 12.4 | -207.184422 | 23.6 | -207.184576 | 52.0 | -207.184611 |
| 3.7 | -207.192244 | 12.6 | -207.184429 | 23.8 | -207.184577 | 54.0 | -207.184611 |
| 3.8 | -207.191505 | 12.8 | -207.184436 | 24.0 | -207.184578 | 56.0 | -207.184612 |
| 3.9 | -207.190795 | 13.0 | -207.184442 | 24.2 | -207.184579 | 58.0 | -207.184612 |
| 4.0 | -207.190120 | 13.2 | -207.184448 | 24.4 | -207.184580 | 60.0 | -207.184612 |
| 4.1 | -207.189490 | 13.4 | -207.184454 | 24.6 | -207.184580 | 62.0 | -207.184612 |
| 4.2 | -207.188905 | 13.6 | -207.184459 | 24.8 | -207.184581 | 64.0 | -207.184613 |
| 4.3 | -207.188364 | 13.8 | -207.184464 | 25.0 | -207.184582 | 66.0 | -207.184613 |
| 4.4 | -207.187871 | 14.0 | -207.184469 | 25.2 | -207.184583 | 68.0 | -207.184613 |
| 4.5 | -207.187426 | 14.2 | -207.184474 | 25.4 | -207.184583 | 70.0 | -207.184613 |
| 4.6 | -207.187020 | 14.4 | -207.184479 | 25.6 | -207.184584 | 72.0 | -207.184613 |
| 4.7 | -207.186658 | 14.6 | -207.184483 | 25.8 | -207.184585 | 74.0 | -207.184613 |
| 4.8 | -207.186336 | 14.8 | -207.184487 | 26.0 | -207.184585 | 76.0 | -207.184613 |
| 4.9 | -207.186048 | 15.0 | -207.184491 | 26.2 | -207.184586 | 78.0 | -207.184614 |
| 5.0 | -207.185793 | 15.2 | -207.184495 | 26.4 | -207.184586 | 80.0 | -207.184614 |
| 5.1 | -207.185565 | 15.4 | -207.184499 | 26.6 | -207.184587 | 82.0 | -207.184614 |
| 5.2 | -207.185367 | 15.6 | -207.184502 | 26.8 | -207.184588 | 84.0 | -207.184614 |
| 5.3 | -207.185193 | 15.8 | -207.184506 | 27.0 | -207.184588 | 86.0 | -207.184614 |
| 5.4 | -207.185041 | 16.0 | -207.184509 | 27.2 | -207.184589 | 88.0 | -207.184614 |
| 5.5 | -207.184908 | 16.2 | -207.184512 | 27.4 | -207.184589 | 90.0 | -207.184614 |
| 5.6 | -207.184793 | 16.4 | -207.184516 | 27.6 | -207.184590 | 92.0 | -207.184614 |
| 5.7 | -207.184694 | 16.6 | -207.184519 | 27.8 | -207.184590 | 94.0 | -207.184614 |
| 5.8 | -207.184608 | 16.8 | -207.184521 | 28.0 | -207.184591 | 96.0 | -207.184614 |
| 5.9 | -207.184535 | 17.0 | -207.184524 | 28.2 | -207.184591 | 98.0 | -207.184614 |
| 6.0 | -207.184472 | 17.2 | -207.184527 | 28.4 | -207.184592 | 100.0 | -207.184614 |
| 6.2 | -207.184373 | 17.4 | -207.184529 | 28.6 | -207.184592 | 150.0 | -207.184615 |
| 6.4 | -207.184303 | 17.6 | -207.184532 | 28.8 | -207.184593 | 200.0 | -207.184615 |
| 6.6 | -207.184254 | 17.8 | -207.184534 | 29.0 | -207.184593 | | |
| 6.8 | -207.184222 | 18.0 | -207.184536 | 29.2 | -207.184593 | | |
| 7.0 | -207.184202 | 18.2 | -207.184539 | 29.4 | -207.184594 | | |
| 7.2 | -207.184192 | 18.4 | -207.184541 | 29.6 | -207.184594 | | |
| 7.4 | -207.184188 | 18.6 | -207.184543 | 29.8 | -207.184595 | | |
| 7.6 | -207.184190 | 18.8 | -207.184545 | 30.0 | -207.184595 | | |
| 7.8 | -207.184195 | 19.0 | -207.184547 | 30.2 | -207.184595 | | |

Table S15: Total IH-FS-CCSD (2,0)/unANO-RCC+ energies for the $2^3\Pi$ state of $LiMg^+$.

| R (Å) | E (a.u.) | R (Å) | E (a.u.) | R (Å) | E (a.u.) | R (Å) | E (a.u.) |
|---|---|---|---|---|---|---|---|
| 1.4 | -206.803500 | 8.0 | -207.132864 | 19.2 | -207.133172 | 30.4 | -207.133226 |
| 1.5 | -206.849861 | 8.2 | -207.132875 | 19.4 | -207.133174 | 30.6 | -207.133226 |
| 1.6 | -206.892290 | 8.4 | -207.132885 | 19.6 | -207.133175 | 30.8 | -207.133227 |
| 1.7 | -206.928168 | 8.6 | -207.132893 | 19.8 | -207.133177 | 31.0 | -207.133227 |
| 1.8 | -206.958480 | 8.8 | -207.132900 | 20.0 | -207.133179 | 31.2 | -207.133227 |
| 1.9 | -206.983978 | 9.0 | -207.132908 | 20.2 | -207.133181 | 31.4 | -207.133228 |
| 2.0 | -207.005322 | 9.2 | -207.132915 | 20.4 | -207.133183 | 31.6 | -207.133228 |
| 2.1 | -207.023112 | 9.4 | -207.132922 | 20.6 | -207.133184 | 31.8 | -207.133228 |
| 2.2 | -207.037896 | 9.6 | -207.132929 | 20.8 | -207.133186 | 32.0 | -207.133229 |
| 2.3 | -207.050177 | 9.8 | -207.132937 | 21.0 | -207.133187 | 33.0 | -207.133230 |
| 2.4 | -207.060390 | 10.0 | -207.132945 | 21.2 | -207.133189 | 34.0 | -207.133232 |
| 2.5 | -207.068918 | 10.2 | -207.132952 | 21.4 | -207.133190 | 35.0 | -207.133233 |
| 2.6 | -207.076078 | 10.4 | -207.132960 | 21.6 | -207.133192 | 36.0 | -207.133234 |
| 2.7 | -207.082135 | 10.6 | -207.132968 | 21.8 | -207.133193 | 37.0 | -207.133235 |
| 2.8 | -207.087308 | 10.8 | -207.132976 | 22.0 | -207.133194 | 38.0 | -207.133236 |
| 2.9 | -207.091773 | 11.0 | -207.132984 | 22.2 | -207.133196 | 39.0 | -207.133237 |
| 3.0 | -207.095670 | 11.2 | -207.132992 | 22.4 | -207.133197 | 40.0 | -207.133238 |
| 3.1 | -207.099112 | 11.4 | -207.133000 | 22.6 | -207.133198 | 42.0 | -207.133239 |
| 3.2 | -207.102186 | 11.6 | -207.133007 | 22.8 | -207.133199 | 44.0 | -207.133240 |
| 3.3 | -207.104958 | 11.8 | -207.133015 | 23.0 | -207.133200 | 46.0 | -207.133241 |
| 3.4 | -207.107479 | 12.0 | -207.133022 | 23.2 | -207.133201 | 48.0 | -207.133242 |
| 3.5 | -207.109788 | 12.2 | -207.133029 | 23.4 | -207.133202 | 50.0 | -207.133243 |
| 3.6 | -207.111913 | 12.4 | -207.133036 | 23.6 | -207.133203 | 52.0 | -207.133243 |
| 3.7 | -207.113875 | 12.6 | -207.133043 | 23.8 | -207.133204 | 54.0 | -207.133244 |
| 3.8 | -207.115689 | 12.8 | -207.133050 | 24.0 | -207.133205 | 56.0 | -207.133244 |
| 3.9 | -207.117367 | 13.0 | -207.133056 | 24.2 | -207.133206 | 58.0 | -207.133245 |
| 4.0 | -207.118916 | 13.2 | -207.133062 | 24.4 | -207.133207 | 60.0 | -207.133245 |
| 4.1 | -207.120344 | 13.4 | -207.133068 | 24.6 | -207.133208 | 62.0 | -207.133245 |
| 4.2 | -207.121657 | 13.6 | -207.133073 | 24.8 | -207.133209 | 64.0 | -207.133246 |
| 4.3 | -207.122860 | 13.8 | -207.133079 | 25.0 | -207.133210 | 66.0 | -207.133246 |
| 4.4 | -207.123959 | 14.0 | -207.133084 | 25.2 | -207.133211 | 68.0 | -207.133246 |
| 4.5 | -207.124959 | 14.2 | -207.133089 | 25.4 | -207.133211 | 70.0 | -207.133246 |
| 4.6 | -207.125866 | 14.4 | -207.133094 | 25.6 | -207.133212 | 72.0 | -207.133247 |
| 4.7 | -207.126686 | 14.6 | -207.133099 | 25.8 | -207.133213 | 74.0 | -207.133247 |
| 4.8 | -207.127425 | 14.8 | -207.133104 | 26.0 | -207.133214 | 76.0 | -207.133247 |
| 4.9 | -207.128089 | 15.0 | -207.133108 | 26.2 | -207.133214 | 78.0 | -207.133247 |
| 5.0 | -207.128682 | 15.2 | -207.133112 | 26.4 | -207.133215 | 80.0 | -207.133247 |
| 5.1 | -207.129211 | 15.4 | -207.133116 | 26.6 | -207.133216 | 82.0 | -207.133247 |
| 5.2 | -207.129682 | 15.6 | -207.133120 | 26.8 | -207.133216 | 84.0 | -207.133247 |
| 5.3 | -207.130099 | 15.8 | -207.133124 | 27.0 | -207.133217 | 86.0 | -207.133247 |
| 5.4 | -207.130468 | 16.0 | -207.133128 | 27.2 | -207.133218 | 88.0 | -207.133247 |
| 5.5 | -207.130793 | 16.2 | -207.133131 | 27.4 | -207.133218 | 90.0 | -207.133247 |
| 5.6 | -207.131079 | 16.4 | -207.133135 | 27.6 | -207.133219 | 92.0 | -207.133248 |
| 5.7 | -207.131330 | 16.6 | -207.133138 | 27.8 | -207.133219 | 94.0 | -207.133248 |
| 5.8 | -207.131549 | 16.8 | -207.133141 | 28.0 | -207.133220 | 96.0 | -207.133248 |
| 5.9 | -207.131741 | 17.0 | -207.133144 | 28.2 | -207.133221 | 98.0 | -207.133248 |
| 6.0 | -207.131907 | 17.2 | -207.133147 | 28.4 | -207.133221 | 100.0 | -207.133248 |
| 6.2 | -207.132176 | 17.4 | -207.133150 | 28.6 | -207.133222 | 150.0 | -207.133248 |
| 6.4 | -207.132375 | 17.6 | -207.133153 | 28.8 | -207.133222 | 200.0 | -207.133248 |
| 6.6 | -207.132522 | 17.8 | -207.133155 | 29.0 | -207.133223 | | |
| 6.8 | -207.132629 | 18.0 | -207.133158 | 29.2 | -207.133223 | | |
| 7.0 | -207.132706 | 18.2 | -207.133160 | 29.4 | -207.133224 | | |
| 7.2 | -207.132761 | 18.4 | -207.133163 | 29.6 | -207.133224 | | |
| 7.4 | -207.132800 | 18.6 | -207.133165 | 29.8 | -207.133224 | | |
| 7.6 | -207.132828 | 18.8 | -207.133167 | 30.0 | -207.133225 | | |
| 7.8 | -207.132849 | 19.0 | -207.133169 | 30.2 | -207.133225 | | |

Table S16: Total IH-FS-CCSD (2,0) DK3/ANO-RCC energies for the $X^1\Sigma^+$ state of $LiMg^+$.

| R (Å) | E (a.u.) | R (Å) | E (a.u.) | R (Å) | E (a.u.) |
|---|---|---|---|---|---|
| 1.4 | -207.352573 | 8.0 | -207.565415 | 14.6 | -207.564735 |
| 1.6 | -207.442780 | 8.2 | -207.565341 | 14.8 | -207.564731 |
| 1.8 | -207.504499 | 8.4 | -207.565276 | 15.0 | -207.564729 |
| 2.0 | -207.545018 | 8.6 | -207.565219 | 15.2 | -207.564726 |
| 2.2 | -207.570040 | 8.8 | -207.565168 | 15.4 | -207.564723 |
| 2.4 | -207.584342 | 9.0 | -207.565123 | 15.6 | -207.564721 |
| 2.6 | -207.591631 | 9.2 | -207.565084 | 15.8 | -207.564718 |
| 2.8 | -207.594396 | 9.4 | -207.565048 | 16.0 | -207.564716 |
| 3.0 | -207.594425 | 9.6 | -207.565016 | 17.0 | -207.564706 |
| 3.2 | -207.592843 | 9.8 | -207.564988 | 18.0 | -207.564700 |
| 3.4 | -207.590389 | 10.0 | -207.564962 | 19.0 | -207.564695 |
| 3.6 | -207.587499 | 10.2 | -207.564939 | 20.0 | -207.564690 |
| 3.8 | -207.584556 | 10.4 | -207.564918 | 21.0 | -207.564687 |
| 4.0 | -207.581718 | 10.6 | -207.564899 | 22.0 | -207.564685 |
| 4.2 | -207.579100 | 10.8 | -207.564882 | 23.0 | -207.564683 |
| 4.4 | -207.576765 | 11.0 | -207.564866 | 24.0 | -207.564681 |
| 4.6 | -207.574737 | 11.2 | -207.564852 | 25.0 | -207.564680 |
| 4.8 | -207.573013 | 11.4 | -207.564840 | 26.0 | -207.564679 |
| 5.0 | -207.571573 | 11.6 | -207.564828 | 27.0 | -207.564678 |
| 5.2 | -207.570385 | 11.8 | -207.564818 | 28.0 | -207.564677 |
| 5.4 | -207.569414 | 12.0 | -207.564808 | 29.0 | -207.564677 |
| 5.6 | -207.568625 | 12.2 | -207.564799 | 30.0 | -207.564676 |
| 5.8 | -207.567986 | 12.4 | -207.564791 | 50.0 | -207.564673 |
| 6.0 | -207.567468 | 12.6 | -207.564784 | 100.0 | -207.564673 |
| 6.2 | -207.567049 | 12.8 | -207.564777 | 200.0 | -207.564673 |
| 6.4 | -207.566701 | 13.0 | -207.564771 | 300.0 | -207.564673 |
| 6.6 | -207.566425 | 13.2 | -207.564765 | | |
| 6.8 | -207.566197 | 13.4 | -207.564760 | | |
| 7.0 | -207.566008 | 13.6 | -207.564755 | | |
| 7.2 | -207.565848 | 13.8 | -207.564750 | | |
| 7.4 | -207.565714 | 14.0 | -207.564746 | | |
| 7.6 | -207.565599 | 14.2 | -207.564742 | | |
| 7.8 | -207.565500 | 14.4 | -207.564738 | | |

Table S17: Total IH-FS-CCSD (2,0) DK3/ANO-RCC energies for the $2^1\Sigma^+$ state of $LiMg^+$.

| R (Å) | E (a.u.) | R (Å) | E (a.u.) | R (Å) | E (a.u.) |
|---|---|---|---|---|---|
| 1.4 | -207.175475 | 8.0 | -207.484060 | 14.6 | -207.482333 |
| 1.6 | -207.261966 | 8.2 | -207.483873 | 14.8 | -207.482325 |
| 1.8 | -207.323567 | 8.4 | -207.483707 | 15.0 | -207.482317 |
| 2.0 | -207.367810 | 8.6 | -207.483559 | 15.2 | -207.482310 |
| 2.2 | -207.398230 | 8.8 | -207.483429 | 15.4 | -207.482304 |
| 2.4 | -207.418180 | 9.0 | -207.483313 | 15.6 | -207.482298 |
| 2.6 | -207.430964 | 9.2 | -207.483210 | 15.8 | -207.482292 |
| 2.8 | -207.439126 | 9.4 | -207.483119 | 16.0 | -207.482287 |
| 3.0 | -207.444993 | 9.6 | -207.483038 | 17.0 | -207.482265 |
| 3.2 | -207.450169 | 9.8 | -207.482965 | 18.0 | -207.482248 |
| 3.4 | -207.455476 | 10.0 | -207.482900 | 19.0 | -207.482236 |
| 3.6 | -207.460999 | 10.2 | -207.482842 | 20.0 | -207.482227 |
| 3.8 | -207.466381 | 10.4 | -207.482790 | 21.0 | -207.482219 |
| 4.0 | -207.471299 | 10.6 | -207.482743 | 22.0 | -207.482213 |
| 4.2 | -207.475538 | 10.8 | -207.482700 | 23.0 | -207.482209 |
| 4.4 | -207.479013 | 11.0 | -207.482662 | 24.0 | -207.482205 |
| 4.6 | -207.481735 | 11.2 | -207.482627 | 25.0 | -207.482202 |
| 4.8 | -207.483767 | 11.4 | -207.482596 | 26.0 | -207.482200 |
| 5.0 | -207.485202 | 11.6 | -207.482567 | 27.0 | -207.482198 |
| 5.2 | -207.486142 | 11.8 | -207.482541 | 28.0 | -207.482196 |
| 5.4 | -207.486688 | 12.0 | -207.482516 | 29.0 | -207.482195 |
| 5.6 | -207.486934 | 12.2 | -207.482494 | 30.0 | -207.482194 |
| 5.8 | -207.486960 | 12.4 | -207.482474 | 50.0 | -207.482187 |
| 6.0 | -207.486831 | 12.6 | -207.482456 | 100.0 | -207.482186 |
| 6.2 | -207.486603 | 12.8 | -207.482439 | 200.0 | -207.482186 |
| 6.4 | -207.486315 | 13.0 | -207.482423 | 300.0 | -207.482186 |
| 6.6 | -207.485996 | 13.2 | -207.482408 | | |
| 6.8 | -207.485669 | 13.4 | -207.482395 | | |
| 7.0 | -207.485349 | 13.6 | -207.482383 | | |
| 7.2 | -207.485044 | 13.8 | -207.482371 | | |
| 7.4 | -207.484762 | 14.0 | -207.482360 | | |
| 7.6 | -207.484503 | 14.2 | -207.482351 | | |
| 7.8 | -207.484270 | 14.4 | -207.482341 | | |

Table S18: Total IH-FS-CCSD (2,0) DK3/ANO-RCC energies for the $3^1\Sigma^+$ state of $LiMg^+$.

| R (Å) | E (a.u.) | R (Å) | E (a.u.) | R (Å) | E (a.u.) |
|---|---|---|---|---|---|
| 1.4 | -207.146100 | 8.0 | -207.422942 | 14.6 | -207.414876 |
| 1.6 | -207.210328 | 8.2 | -207.422355 | 14.8 | -207.414846 |
| 1.8 | -207.270946 | 8.4 | -207.421752 | 15.0 | -207.414819 |
| 2.0 | -207.314901 | 8.6 | -207.421146 | 15.2 | -207.414793 |
| 2.2 | -207.344830 | 8.8 | -207.420548 | 15.4 | -207.414768 |
| 2.4 | -207.365347 | 9.0 | -207.419970 | 15.6 | -207.414745 |
| 2.6 | -207.379714 | 9.2 | -207.419419 | 15.8 | -207.414723 |
| 2.8 | -207.389989 | 9.4 | -207.418902 | 16.0 | -207.414702 |
| 3.0 | -207.397301 | 9.6 | -207.418425 | 17.0 | -207.414615 |
| 3.2 | -207.402168 | 9.8 | -207.417991 | 18.0 | -207.414548 |
| 3.4 | -207.405047 | 10.0 | -207.417602 | 19.0 | -207.414495 |
| 3.6 | -207.406682 | 10.2 | -207.417256 | 20.0 | -207.414453 |
| 3.8 | -207.407927 | 10.4 | -207.416951 | 21.0 | -207.414419 |
| 4.0 | -207.409402 | 10.6 | -207.416685 | 22.0 | -207.414391 |
| 4.2 | -207.411235 | 10.8 | -207.416454 | 23.0 | -207.414368 |
| 4.4 | -207.413266 | 11.0 | -207.416253 | 24.0 | -207.414349 |
| 4.6 | -207.415304 | 11.2 | -207.416079 | 25.0 | -207.414333 |
| 4.8 | -207.417228 | 11.4 | -207.415928 | 26.0 | -207.414320 |
| 5.0 | -207.418971 | 11.6 | -207.415796 | 27.0 | -207.414308 |
| 5.2 | -207.420502 | 11.8 | -207.415680 | 28.0 | -207.414298 |
| 5.4 | -207.421811 | 12.0 | -207.415578 | 29.0 | -207.414290 |
| 5.6 | -207.422896 | 12.2 | -207.415488 | 30.0 | -207.414283 |
| 5.8 | -207.423765 | 12.4 | -207.415407 | 50.0 | -207.414230 |
| 6.0 | -207.424426 | 12.6 | -207.415334 | 100.0 | -207.414218 |
| 6.2 | -207.424892 | 12.8 | -207.415269 | 200.0 | -207.414217 |
| 6.4 | -207.425181 | 13.0 | -207.415209 | 300.0 | -207.414217 |
| 6.6 | -207.425299 | 13.2 | -207.415155 | | |
| 6.8 | -207.425269 | 13.4 | -207.415105 | | |
| 7.0 | -207.425107 | 13.6 | -207.415060 | | |
| 7.2 | -207.424832 | 13.8 | -207.415017 | | |
| 7.4 | -207.424460 | 14.0 | -207.414978 | | |
| 7.6 | -207.424010 | 14.2 | -207.414942 | | |
| 7.8 | -207.423499 | 14.4 | -207.414908 | | |

Table S19: Total IH-FS-CCSD (2,0) DK3/ANO-RCC energies for the $4^1\Sigma^+$ state of $LiMg^+$.

| R (Å) | E (a.u.) | R (Å) | E (a.u.) | R (Å) | E (a.u.) |
|---|---|---|---|---|---|
| 1.4 | -207.121479 | 8.0 | -207.403623 | 14.6 | -207.404785 |
| 1.6 | -207.207748 | 8.2 | -207.404275 | 14.8 | -207.404751 |
| 1.8 | -207.251771 | 8.4 | -207.404835 | 15.0 | -207.404719 |
| 2.0 | -207.280816 | 8.6 | -207.405309 | 15.2 | -207.404688 |
| 2.2 | -207.301303 | 8.8 | -207.405699 | 15.4 | -207.404660 |
| 2.4 | -207.315288 | 9.0 | -207.406012 | 15.6 | -207.404633 |
| 2.6 | -207.325400 | 9.2 | -207.406252 | 15.8 | -207.404608 |
| 2.8 | -207.342097 | 9.4 | -207.406424 | 16.0 | -207.404585 |
| 3.0 | -207.355826 | 9.6 | -207.406535 | 17.0 | -207.404485 |
| 3.2 | -207.366607 | 9.8 | -207.406591 | 18.0 | -207.404409 |
| 3.4 | -207.374636 | 10.0 | -207.406599 | 19.0 | -207.404349 |
| 3.6 | -207.380172 | 10.2 | -207.406567 | 20.0 | -207.404302 |
| 3.8 | -207.383487 | 10.4 | -207.406503 | 21.0 | -207.404264 |
| 4.0 | -207.384940 | 10.6 | -207.406415 | 22.0 | -207.404233 |
| 4.2 | -207.385131 | 10.8 | -207.406311 | 23.0 | -207.404208 |
| 4.4 | -207.384745 | 11.0 | -207.406196 | 24.0 | -207.404187 |
| 4.6 | -207.384319 | 11.2 | -207.406076 | 25.0 | -207.404170 |
| 4.8 | -207.384203 | 11.4 | -207.405955 | 26.0 | -207.404155 |
| 5.0 | -207.384558 | 11.6 | -207.405837 | 27.0 | -207.404143 |
| 5.2 | -207.385395 | 11.8 | -207.405724 | 28.0 | -207.404132 |
| 5.4 | -207.386623 | 12.0 | -207.405616 | 29.0 | -207.404123 |
| 5.6 | -207.388114 | 12.2 | -207.405516 | 30.0 | -207.404115 |
| 5.8 | -207.389745 | 12.4 | -207.405423 | 50.0 | -207.404060 |
| 6.0 | -207.391422 | 12.6 | -207.405337 | 100.0 | -207.404048 |
| 6.2 | -207.393076 | 12.8 | -207.405258 | 200.0 | -207.404047 |
| 6.4 | -207.394645 | 13.0 | -207.405186 | 300.0 | -207.404047 |
| 6.6 | -207.396149 | 13.2 | -207.405120 | | |
| 6.8 | -207.397547 | 13.4 | -207.405059 | | |
| 7.0 | -207.398834 | 13.6 | -207.405004 | | |
| 7.2 | -207.400009 | 13.8 | -207.404953 | | |
| 7.4 | -207.401071 | 14.0 | -207.404906 | | |
| 7.6 | -207.402025 | 14.2 | -207.404862 | | |
| 7.8 | -207.402874 | 14.4 | -207.404822 | | |

Table S20: Total IH-FS-CCSD (2,0) DK3/ANO-RCC energies for the $5^1\Sigma^+$ state of $LiMg^+$.

| R (Å) | E (a.u.) | R (Å) | E (a.u.) | R (Å) | E (a.u.) |
|---|---|---|---|---|---|
| 1.4 | -207.028206 | 8.0 | -207.365308 | 14.6 | -207.365144 |
| 1.6 | -207.120052 | 8.2 | -207.366230 | 14.8 | -207.365095 |
| 1.8 | -207.177816 | 8.4 | -207.367051 | 15.0 | -207.365056 |
| 2.0 | -207.216127 | 8.6 | -207.367768 | 15.2 | -207.365025 |
| 2.2 | -207.249517 | 8.8 | -207.368380 | 15.4 | -207.365001 |
| 2.4 | -207.275805 | 9.0 | -207.368887 | 15.6 | -207.364981 |
| 2.6 | -207.294920 | 9.2 | -207.369291 | 15.8 | -207.364965 |
| 2.8 | -207.308036 | 9.4 | -207.369594 | 16.0 | -207.364952 |
| 3.0 | -207.317151 | 9.6 | -207.369802 | 17.0 | -207.364920 |
| 3.2 | -207.323822 | 9.8 | -207.369918 | 18.0 | -207.364910 |
| 3.4 | -207.329069 | 10.0 | -207.369948 | 19.0 | -207.364907 |
| 3.6 | -207.333441 | 10.2 | -207.369901 | 20.0 | -207.364906 |
| 3.8 | -207.337341 | 10.4 | -207.369783 | 21.0 | -207.364905 |
| 4.0 | -207.340869 | 10.6 | -207.369605 | 22.0 | -207.364905 |
| 4.2 | -207.344073 | 10.8 | -207.369375 | 23.0 | -207.364905 |
| 4.4 | -207.346932 | 11.0 | -207.369104 | 24.0 | -207.364904 |
| 4.6 | -207.349381 | 11.2 | -207.368802 | 25.0 | -207.364904 |
| 4.8 | -207.351347 | 11.4 | -207.368480 | 26.0 | -207.364904 |
| 5.0 | -207.352796 | 11.6 | -207.368147 | 27.0 | -207.364904 |
| 5.2 | -207.353770 | 11.8 | -207.367812 | 28.0 | -207.364904 |
| 5.4 | -207.354378 | 12.0 | -207.367481 | 29.0 | -207.364904 |
| 5.6 | -207.354760 | 12.2 | -207.367165 | 30.0 | -207.364904 |
| 5.8 | -207.355057 | 12.4 | -207.366867 | 50.0 | -207.364904 |
| 6.0 | -207.355391 | 12.6 | -207.366591 | 100.0 | -207.364904 |
| 6.2 | -207.355857 | 12.8 | -207.366339 | 200.0 | -207.364904 |
| 6.4 | -207.356514 | 13.0 | -207.366114 | 300.0 | -207.364904 |
| 6.6 | -207.357387 | 13.2 | -207.365916 | | |
| 6.8 | -207.358437 | 13.4 | -207.365743 | | |
| 7.0 | -207.359600 | 13.6 | -207.365595 | | |
| 7.2 | -207.360812 | 13.8 | -207.365469 | | |
| 7.4 | -207.362022 | 14.0 | -207.365363 | | |
| 7.6 | -207.363190 | 14.2 | -207.365275 | | |
| 7.8 | -207.364291 | 14.4 | -207.365203 | | |

Table S21: Total IH-FS-CCSD (2,0) DK3/ANO-RCC energies for the $6^1\Sigma^+$ state of $LiMg^+$.

| R (Å) | E (a.u.) | R (Å) | E (a.u.) | R (Å) | E (a.u.) |
|---|---|---|---|---|---|
| 1.4 | -207.012239 | 8.0 | -207.349135 | 14.6 | -207.355653 |
| 1.6 | -207.100448 | 8.2 | -207.349243 | 14.8 | -207.355612 |
| 1.8 | -207.160320 | 8.4 | -207.349368 | 15.0 | -207.355569 |
| 2.0 | -207.201624 | 8.6 | -207.349515 | 15.2 | -207.355524 |
| 2.2 | -207.230169 | 8.8 | -207.349690 | 15.4 | -207.355479 |
| 2.4 | -207.252355 | 9.0 | -207.349901 | 15.6 | -207.355433 |
| 2.6 | -207.269946 | 9.2 | -207.350150 | 15.8 | -207.355388 |
| 2.8 | -207.282772 | 9.4 | -207.350438 | 16.0 | -207.355345 |
| 3.0 | -207.292197 | 9.6 | -207.350765 | 17.0 | -207.355151 |
| 3.2 | -207.299383 | 9.8 | -207.351122 | 18.0 | -207.355002 |
| 3.4 | -207.305200 | 10.0 | -207.351503 | 19.0 | -207.354890 |
| 3.6 | -207.310297 | 10.2 | -207.351897 | 20.0 | -207.354805 |
| 3.8 | -207.315008 | 10.4 | -207.352296 | 21.0 | -207.354742 |
| 4.0 | -207.319481 | 10.6 | -207.352693 | 22.0 | -207.354692 |
| 4.2 | -207.323662 | 10.8 | -207.353079 | 23.0 | -207.354654 |
| 4.4 | -207.327430 | 11.0 | -207.353449 | 24.0 | -207.354624 |
| 4.6 | -207.330705 | 11.2 | -207.353798 | 25.0 | -207.354600 |
| 4.8 | -207.333490 | 11.4 | -207.354124 | 26.0 | -207.354580 |
| 5.0 | -207.335859 | 11.6 | -207.354421 | 27.0 | -207.354564 |
| 5.2 | -207.337917 | 11.8 | -207.354687 | 28.0 | -207.354551 |
| 5.4 | -207.339762 | 12.0 | -207.354922 | 29.0 | -207.354540 |
| 5.6 | -207.341465 | 12.2 | -207.355126 | 30.0 | -207.354531 |
| 5.8 | -207.343049 | 12.4 | -207.355298 | 50.0 | -207.354479 |
| 6.0 | -207.344493 | 12.6 | -207.355439 | 100.0 | -207.354472 |
| 6.2 | -207.345751 | 12.8 | -207.355551 | 200.0 | -207.354472 |
| 6.4 | -207.346731 | 13.0 | -207.355636 | 300.0 | -207.354472 |
| 6.6 | -207.347496 | 13.2 | -207.355696 | | |
| 6.8 | -207.348034 | 13.4 | -207.355733 | | |
| 7.0 | -207.348396 | 13.6 | -207.355751 | | |
| 7.2 | -207.348637 | 13.8 | -207.355753 | | |
| 7.4 | -207.348804 | 14.0 | -207.355742 | | |
| 7.6 | -207.348928 | 14.2 | -207.355720 | | |
| 7.8 | -207.349033 | 14.4 | -207.355689 | | |

Table S22: Total IH-FS-CCSD (2,0) DK3/ANO-RCC energies for the $1^3\Sigma^+$ state of $LiMg^+$.

| R (Å) | E (a.u.) | R (Å) | E (a.u.) | R (Å) | E (a.u.) |
|---|---|---|---|---|---|
| 1.4 | -207.212409 | 8.0 | -207.484468 | 14.6 | -207.482333 |
| 1.6 | -207.301367 | 8.2 | -207.484161 | 14.8 | -207.482325 |
| 1.8 | -207.370080 | 8.4 | -207.483908 | 15.0 | -207.482317 |
| 2.0 | -207.421066 | 8.6 | -207.483700 | 15.2 | -207.482310 |
| 2.2 | -207.456723 | 8.8 | -207.483526 | 15.4 | -207.482304 |
| 2.4 | -207.480772 | 9.0 | -207.483380 | 15.6 | -207.482298 |
| 2.6 | -207.496676 | 9.2 | -207.483256 | 15.8 | -207.482292 |
| 2.8 | -207.506730 | 9.4 | -207.483150 | 16.0 | -207.482287 |
| 3.0 | -207.512832 | 9.6 | -207.483059 | 17.0 | -207.482265 |
| 3.2 | -207.516191 | 9.8 | -207.482980 | 18.0 | -207.482248 |
| 3.4 | -207.517643 | 10.0 | -207.482910 | 19.0 | -207.482236 |
| 3.6 | -207.517749 | 10.2 | -207.482849 | 20.0 | -207.482227 |
| 3.8 | -207.516953 | 10.4 | -207.482794 | 21.0 | -207.482219 |
| 4.0 | -207.515523 | 10.6 | -207.482746 | 22.0 | -207.482213 |
| 4.2 | -207.513667 | 10.8 | -207.482703 | 23.0 | -207.482209 |
| 4.4 | -207.511538 | 11.0 | -207.482663 | 24.0 | -207.482205 |
| 4.6 | -207.509249 | 11.2 | -207.482628 | 25.0 | -207.482202 |
| 4.8 | -207.506885 | 11.4 | -207.482596 | 26.0 | -207.482200 |
| 5.0 | -207.504512 | 11.6 | -207.482567 | 27.0 | -207.482198 |
| 5.2 | -207.502181 | 11.8 | -207.482541 | 28.0 | -207.482196 |
| 5.4 | -207.499932 | 12.0 | -207.482517 | 29.0 | -207.482195 |
| 5.6 | -207.497797 | 12.2 | -207.482495 | 30.0 | -207.482194 |
| 5.8 | -207.495801 | 12.4 | -207.482474 | 50.0 | -207.482187 |
| 6.0 | -207.493961 | 12.6 | -207.482456 | 100.0 | -207.482186 |
| 6.2 | -207.492291 | 12.8 | -207.482439 | 200.0 | -207.482186 |
| 6.4 | -207.490796 | 13.0 | -207.482423 | 300.0 | -207.482186 |
| 6.6 | -207.489480 | 13.2 | -207.482408 | | |
| 6.8 | -207.488340 | 13.4 | -207.482395 | | |
| 7.0 | -207.487367 | 13.6 | -207.482383 | | |
| 7.2 | -207.486548 | 13.8 | -207.482371 | | |
| 7.4 | -207.485866 | 14.0 | -207.482360 | | |
| 7.6 | -207.485304 | 14.2 | -207.482351 | | |
| 7.8 | -207.484844 | 14.4 | -207.482341 | | |

Table S23: Total IH-FS-CCSD (2,0) DK3/ANO-RCC energies for the $2^3\Sigma^+$ state of $LiMg^+$.

| R (Å) | E (a.u.) | R (Å) | E (a.u.) | R (Å) | E (a.u.) |
|---|---|---|---|---|---|
| 1.4 | -207.123408 | 8.0 | -207.468856 | 14.6 | -207.465973 |
| 1.6 | -207.227173 | 8.2 | -207.468696 | 14.8 | -207.465951 |
| 1.8 | -207.293123 | 8.4 | -207.468521 | 15.0 | -207.465931 |
| 2.0 | -207.335288 | 8.6 | -207.468341 | 15.2 | -207.465912 |
| 2.2 | -207.362959 | 8.8 | -207.468160 | 15.4 | -207.465893 |
| 2.4 | -207.381534 | 9.0 | -207.467986 | 15.6 | -207.465876 |
| 2.6 | -207.394460 | 9.2 | -207.467819 | 15.8 | -207.465860 |
| 2.8 | -207.403959 | 9.4 | -207.467662 | 16.0 | -207.465844 |
| 3.0 | -207.411446 | 9.6 | -207.467515 | 17.0 | -207.465778 |
| 3.2 | -207.417753 | 9.8 | -207.467379 | 18.0 | -207.465726 |
| 3.4 | -207.423355 | 10.0 | -207.467253 | 19.0 | -207.465685 |
| 3.6 | -207.428498 | 10.2 | -207.467137 | 20.0 | -207.465652 |
| 3.8 | -207.433317 | 10.4 | -207.467030 | 21.0 | -207.465626 |
| 4.0 | -207.437851 | 10.6 | -207.466932 | 22.0 | -207.465604 |
| 4.2 | -207.442100 | 10.8 | -207.466842 | 23.0 | -207.465587 |
| 4.4 | -207.446044 | 11.0 | -207.466759 | 24.0 | -207.465572 |
| 4.6 | -207.449663 | 11.2 | -207.466682 | 25.0 | -207.465559 |
| 4.8 | -207.452939 | 11.4 | -207.466612 | 26.0 | -207.465549 |
| 5.0 | -207.455864 | 11.6 | -207.466547 | 27.0 | -207.465540 |
| 5.2 | -207.458441 | 11.8 | -207.466487 | 28.0 | -207.465532 |
| 5.4 | -207.460680 | 12.0 | -207.466431 | 29.0 | -207.465525 |
| 5.6 | -207.462597 | 12.2 | -207.466379 | 30.0 | -207.465519 |
| 5.8 | -207.464214 | 12.4 | -207.466331 | 50.0 | -207.465478 |
| 6.0 | -207.465552 | 12.6 | -207.466286 | 100.0 | -207.465469 |
| 6.2 | -207.466636 | 12.8 | -207.466245 | 200.0 | -207.465468 |
| 6.4 | -207.467469 | 13.0 | -207.466206 | 300.0 | -207.465468 |
| 6.6 | -207.468114 | 13.2 | -207.466170 | | |
| 6.8 | -207.468575 | 13.4 | -207.466136 | | |
| 7.0 | -207.468877 | 13.6 | -207.466104 | | |
| 7.2 | -207.469044 | 13.8 | -207.466075 | | |
| 7.4 | -207.469103 | 14.0 | -207.466047 | | |
| 7.6 | -207.469077 | 14.2 | -207.466021 | | |
| 7.8 | -207.468988 | 14.4 | -207.465996 | | |

Table S24: Total IH-FS-CCSD (2,0) DK3/ANO-RCC energies for the $3^3\Sigma^+$ state of $LiMg^+$.

| R (Å) | E (a.u.) | R (Å) | E (a.u.) | R (Å) | E (a.u.) |
|---|---|---|---|---|---|
| 1.4 | -207.037340 | 8.0 | -207.417924 | 14.6 | -207.414875 |
| 1.6 | -207.132332 | 8.2 | -207.417944 | 14.8 | -207.414846 |
| 1.8 | -207.199409 | 8.4 | -207.417906 | 15.0 | -207.414818 |
| 2.0 | -207.242571 | 8.6 | -207.417822 | 15.2 | -207.414792 |
| 2.2 | -207.280226 | 8.8 | -207.417704 | 15.4 | -207.414768 |
| 2.4 | -207.302217 | 9.0 | -207.417561 | 15.6 | -207.414745 |
| 2.6 | -207.316805 | 9.2 | -207.417402 | 15.8 | -207.414723 |
| 2.8 | -207.326468 | 9.4 | -207.417234 | 16.0 | -207.414702 |
| 3.0 | -207.333039 | 9.6 | -207.417062 | 17.0 | -207.414615 |
| 3.2 | -207.338051 | 9.8 | -207.416891 | 18.0 | -207.414548 |
| 3.4 | -207.345562 | 10.0 | -207.416724 | 19.0 | -207.414495 |
| 3.6 | -207.354783 | 10.2 | -207.416564 | 20.0 | -207.414453 |
| 3.8 | -207.363456 | 10.4 | -207.416411 | 21.0 | -207.414419 |
| 4.0 | -207.371323 | 10.6 | -207.416268 | 22.0 | -207.414391 |
| 4.2 | -207.378317 | 10.8 | -207.416135 | 23.0 | -207.414368 |
| 4.4 | -207.384442 | 11.0 | -207.416011 | 24.0 | -207.414349 |
| 4.6 | -207.389756 | 11.2 | -207.415897 | 25.0 | -207.414333 |
| 4.8 | -207.394340 | 11.4 | -207.415791 | 26.0 | -207.414320 |
| 5.0 | -207.398282 | 11.6 | -207.415694 | 27.0 | -207.414308 |
| 5.2 | -207.401668 | 11.8 | -207.415605 | 28.0 | -207.414298 |
| 5.4 | -207.404574 | 12.0 | -207.415522 | 29.0 | -207.414290 |
| 5.6 | -207.407064 | 12.2 | -207.415446 | 30.0 | -207.414283 |
| 5.8 | -207.409193 | 12.4 | -207.415377 | 50.0 | -207.414230 |
| 6.0 | -207.411005 | 12.6 | -207.415312 | 100.0 | -207.414218 |
| 6.2 | -207.412537 | 12.8 | -207.415253 | 200.0 | -207.414217 |
| 6.4 | -207.413813 | 13.0 | -207.415198 | 300.0 | -207.414217 |
| 6.6 | -207.414878 | 13.2 | -207.415147 | | |
| 6.8 | -207.415746 | 13.4 | -207.415099 | | |
| 7.0 | -207.416438 | 13.6 | -207.415055 | | |
| 7.2 | -207.416975 | 13.8 | -207.415014 | | |
| 7.4 | -207.417375 | 14.0 | -207.414976 | | |
| 7.6 | -207.417655 | 14.2 | -207.414940 | | |
| 7.8 | -207.417833 | 14.4 | -207.414907 | | |

Table S25: Total IH-FS-CCSD (2,0) DK3/ANO-RCC energies for the $4^3\Sigma^+$ state of $LiMg^+$.

| R (Å) | E (a.u.) | R (Å) | E (a.u.) | R (Å) | E (a.u.) |
|---|---|---|---|---|---|
| 1.4 | -206.994033 | 8.0 | -207.377068 | 14.6 | -207.376862 |
| 1.6 | -207.089249 | 8.2 | -207.377910 | 14.8 | -207.376833 |
| 1.8 | -207.162104 | 8.4 | -207.378625 | 15.0 | -207.376808 |
| 2.0 | -207.214517 | 8.6 | -207.379213 | 15.2 | -207.376786 |
| 2.2 | -207.251275 | 8.8 | -207.379677 | 15.4 | -207.376768 |
| 2.4 | -207.277235 | 9.0 | -207.380024 | 15.6 | -207.376751 |
| 2.6 | -207.296289 | 9.2 | -207.380262 | 15.8 | -207.376737 |
| 2.8 | -207.311096 | 9.4 | -207.380399 | 16.0 | -207.376724 |
| 3.0 | -207.323474 | 9.6 | -207.380447 | 17.0 | -207.376678 |
| 3.2 | -207.334136 | 9.8 | -207.380416 | 18.0 | -207.376648 |
| 3.4 | -207.340303 | 10.0 | -207.380319 | 19.0 | -207.376628 |
| 3.6 | -207.343386 | 10.2 | -207.380169 | 20.0 | -207.376612 |
| 3.8 | -207.345918 | 10.4 | -207.379976 | 21.0 | -207.376600 |
| 4.0 | -207.348187 | 10.6 | -207.379754 | 22.0 | -207.376591 |
| 4.2 | -207.350269 | 10.8 | -207.379513 | 23.0 | -207.376583 |
| 4.4 | -207.352186 | 11.0 | -207.379262 | 24.0 | -207.376578 |
| 4.6 | -207.353950 | 11.2 | -207.379010 | 25.0 | -207.376573 |
| 4.8 | -207.355577 | 11.4 | -207.378763 | 26.0 | -207.376569 |
| 5.0 | -207.357088 | 11.6 | -207.378528 | 27.0 | -207.376566 |
| 5.2 | -207.358514 | 11.8 | -207.378306 | 28.0 | -207.376563 |
| 5.4 | -207.359889 | 12.0 | -207.378102 | 29.0 | -207.376561 |
| 5.6 | -207.361245 | 12.2 | -207.377916 | 30.0 | -207.376559 |
| 5.8 | -207.362608 | 12.4 | -207.377750 | 50.0 | -207.376548 |
| 6.0 | -207.363996 | 12.6 | -207.377601 | 100.0 | -207.376546 |
| 6.2 | -207.365414 | 12.8 | -207.377471 | 200.0 | -207.376546 |
| 6.4 | -207.366856 | 13.0 | -207.377357 | 300.0 | -207.376546 |
| 6.6 | -207.368309 | 13.2 | -207.377258 | | |
| 6.8 | -207.369755 | 13.4 | -207.377172 | | |
| 7.0 | -207.371171 | 13.6 | -207.377099 | | |
| 7.2 | -207.372534 | 13.8 | -207.377036 | | |
| 7.4 | -207.373821 | 14.0 | -207.376982 | | |
| 7.6 | -207.375015 | 14.2 | -207.376935 | | |
| 7.8 | -207.376101 | 14.4 | -207.376896 | | |

Table S26: Total IH-FS-CCSD (2,0) DK3/ANO-RCC energies for the $5^3\Sigma^+$ state of $LiMg^+$.

| R (Å) | E (a.u.) | R (Å) | E (a.u.) | R (Å) | E (a.u.) |
|---|---|---|---|---|---|
| 1.4 | -206.992518 | 8.0 | -207.347823 | 14.6 | -207.355798 |
| 1.6 | -207.073635 | 8.2 | -207.348972 | 14.8 | -207.355727 |
| 1.8 | -207.139808 | 8.4 | -207.350007 | 15.0 | -207.355659 |
| 2.0 | -207.185227 | 8.6 | -207.350935 | 15.2 | -207.355595 |
| 2.2 | -207.216366 | 8.8 | -207.351765 | 15.4 | -207.355534 |
| 2.4 | -207.238085 | 9.0 | -207.352505 | 15.6 | -207.355477 |
| 2.6 | -207.255150 | 9.2 | -207.353164 | 15.8 | -207.355422 |
| 2.8 | -207.268767 | 9.4 | -207.353749 | 16.0 | -207.355371 |
| 3.0 | -207.279055 | 9.6 | -207.354266 | 17.0 | -207.355159 |
| 3.2 | -207.286970 | 9.8 | -207.354720 | 18.0 | -207.355004 |
| 3.4 | -207.293398 | 10.0 | -207.355117 | 19.0 | -207.354890 |
| 3.6 | -207.299143 | 10.2 | -207.355460 | 20.0 | -207.354806 |
| 3.8 | -207.304655 | 10.4 | -207.355753 | 21.0 | -207.354742 |
| 4.0 | -207.309651 | 10.6 | -207.355999 | 22.0 | -207.354692 |
| 4.2 | -207.313794 | 10.8 | -207.356201 | 23.0 | -207.354654 |
| 4.4 | -207.317074 | 11.0 | -207.356362 | 24.0 | -207.354624 |
| 4.6 | -207.319647 | 11.2 | -207.356485 | 25.0 | -207.354600 |
| 4.8 | -207.321760 | 11.4 | -207.356574 | 26.0 | -207.354580 |
| 5.0 | -207.323725 | 11.6 | -207.356630 | 27.0 | -207.354564 |
| 5.2 | -207.325746 | 11.8 | -207.356657 | 28.0 | -207.354551 |
| 5.4 | -207.327770 | 12.0 | -207.356660 | 29.0 | -207.354540 |
| 5.6 | -207.329691 | 12.2 | -207.356642 | 30.0 | -207.354531 |
| 5.8 | -207.331466 | 12.4 | -207.356606 | 50.0 | -207.354479 |
| 6.0 | -207.333095 | 12.6 | -207.356556 | 100.0 | -207.354472 |
| 6.2 | -207.334606 | 12.8 | -207.356496 | 200.0 | -207.354472 |
| 6.4 | -207.336047 | 13.0 | -207.356426 | 300.0 | -207.354472 |
| 6.6 | -207.337476 | 13.2 | -207.356351 | | |
| 6.8 | -207.338952 | 13.4 | -207.356272 | | |
| 7.0 | -207.340502 | 13.6 | -207.356191 | | |
| 7.2 | -207.342094 | 13.8 | -207.356110 | | |
| 7.4 | -207.343667 | 14.0 | -207.356028 | | |
| 7.6 | -207.345163 | 14.2 | -207.355949 | | |
| 7.8 | -207.346553 | 14.4 | -207.355872 | | |

Table S27: Total IH-FS-CCSD (2,0) DK3/ANO-RCC energies for the $1^1\Pi$ state of $LiMg^+$.

| R (Å) | E (a.u.) | R (Å) | E (a.u.) | R (Å) | E (a.u.) |
|---|---|---|---|---|---|
| 1.4 | -207.206075 | 8.0 | -207.414083 | 14.6 | -207.414066 |
| 1.6 | -207.279738 | 8.2 | -207.414018 | 14.8 | -207.414070 |
| 1.8 | -207.329354 | 8.4 | -207.413970 | 15.0 | -207.414075 |
| 2.0 | -207.363834 | 8.6 | -207.413937 | 15.2 | -207.414079 |
| 2.2 | -207.387108 | 8.8 | -207.413915 | 15.4 | -207.414083 |
| 2.4 | -207.402068 | 9.0 | -207.413902 | 15.6 | -207.414088 |
| 2.6 | -207.411175 | 9.2 | -207.413894 | 15.8 | -207.414091 |
| 2.8 | -207.416305 | 9.4 | -207.413892 | 16.0 | -207.414095 |
| 3.0 | -207.419055 | 9.6 | -207.413893 | 17.0 | -207.414112 |
| 3.2 | -207.420280 | 9.8 | -207.413897 | 18.0 | -207.414126 |
| 3.4 | -207.420707 | 10.0 | -207.413903 | 19.0 | -207.414137 |
| 3.6 | -207.420774 | 10.2 | -207.413910 | 20.0 | -207.414147 |
| 3.8 | -207.420704 | 10.4 | -207.413917 | 21.0 | -207.414155 |
| 4.0 | -207.420555 | 10.6 | -207.413926 | 22.0 | -207.414162 |
| 4.2 | -207.420344 | 10.8 | -207.413934 | 23.0 | -207.414168 |
| 4.4 | -207.420068 | 11.0 | -207.413943 | 24.0 | -207.414173 |
| 4.6 | -207.419732 | 11.2 | -207.413952 | 25.0 | -207.414178 |
| 4.8 | -207.419341 | 11.4 | -207.413960 | 26.0 | -207.414182 |
| 5.0 | -207.418905 | 11.6 | -207.413969 | 27.0 | -207.414185 |
| 5.2 | -207.418437 | 11.8 | -207.413977 | 28.0 | -207.414188 |
| 5.4 | -207.417955 | 12.0 | -207.413985 | 29.0 | -207.414191 |
| 5.6 | -207.417471 | 12.2 | -207.413993 | 30.0 | -207.414193 |
| 5.8 | -207.416999 | 12.4 | -207.414000 | 50.0 | -207.414211 |
| 6.0 | -207.416548 | 12.6 | -207.414007 | 100.0 | -207.414216 |
| 6.2 | -207.416126 | 12.8 | -207.414014 | 200.0 | -207.414217 |
| 6.4 | -207.415739 | 13.0 | -207.414021 | 300.0 | -207.414217 |
| 6.6 | -207.415393 | 13.2 | -207.414027 | | |
| 6.8 | -207.415089 | 13.4 | -207.414033 | | |
| 7.0 | -207.414828 | 13.6 | -207.414039 | | |
| 7.2 | -207.414609 | 13.8 | -207.414045 | | |
| 7.4 | -207.414429 | 14.0 | -207.414050 | | |
| 7.6 | -207.414284 | 14.2 | -207.414056 | | |
| 7.8 | -207.414170 | 14.4 | -207.414061 | | |

Table S28: Total IH-FS-CCSD (2,0) DK3/ANO-RCC energies for the $2^1\Pi$ state of $LiMg^+$.

| R (Å) | E (a.u.) | R (Å) | E (a.u.) | R (Å) | E (a.u.) |
|---|---|---|---|---|---|
| 1.4 | -207.103776 | 8.0 | -207.403546 | 14.6 | -207.403893 |
| 1.6 | -207.192743 | 8.2 | -207.403608 | 14.8 | -207.403898 |
| 1.8 | -207.257276 | 8.4 | -207.403650 | 15.0 | -207.403902 |
| 2.0 | -207.302707 | 8.6 | -207.403678 | 15.2 | -207.403907 |
| 2.2 | -207.334080 | 8.8 | -207.403697 | 15.4 | -207.403911 |
| 2.4 | -207.355615 | 9.0 | -207.403709 | 15.6 | -207.403916 |
| 2.6 | -207.370403 | 9.2 | -207.403717 | 15.8 | -207.403920 |
| 2.8 | -207.380427 | 9.4 | -207.403723 | 16.0 | -207.403923 |
| 3.0 | -207.387279 | 9.6 | -207.403727 | 17.0 | -207.403941 |
| 3.2 | -207.391665 | 9.8 | -207.403732 | 18.0 | -207.403955 |
| 3.4 | -207.394259 | 10.0 | -207.403736 | 19.0 | -207.403966 |
| 3.6 | -207.395530 | 10.2 | -207.403741 | 20.0 | -207.403976 |
| 3.8 | -207.395960 | 10.4 | -207.403747 | 21.0 | -207.403984 |
| 4.0 | -207.395926 | 10.6 | -207.403753 | 22.0 | -207.403991 |
| 4.2 | -207.395734 | 10.8 | -207.403760 | 23.0 | -207.403997 |
| 4.4 | -207.395615 | 11.0 | -207.403767 | 24.0 | -207.404002 |
| 4.6 | -207.395694 | 11.2 | -207.403775 | 25.0 | -207.404006 |
| 4.8 | -207.396010 | 11.4 | -207.403783 | 26.0 | -207.404010 |
| 5.0 | -207.396535 | 11.6 | -207.403791 | 27.0 | -207.404013 |
| 5.2 | -207.397209 | 11.8 | -207.403799 | 28.0 | -207.404016 |
| 5.4 | -207.397966 | 12.0 | -207.403806 | 29.0 | -207.404019 |
| 5.6 | -207.398748 | 12.2 | -207.403814 | 30.0 | -207.404021 |
| 5.8 | -207.399512 | 12.4 | -207.403822 | 50.0 | -207.404038 |
| 6.0 | -207.400227 | 12.6 | -207.403830 | 100.0 | -207.404046 |
| 6.2 | -207.400876 | 12.8 | -207.403837 | 200.0 | -207.404047 |
| 6.4 | -207.401444 | 13.0 | -207.403844 | 300.0 | -207.404047 |
| 6.6 | -207.401937 | 13.2 | -207.403851 | | |
| 6.8 | -207.402350 | 13.4 | -207.403858 | | |
| 7.0 | -207.402690 | 13.6 | -207.403864 | | |
| 7.2 | -207.402962 | 13.8 | -207.403870 | | |
| 7.4 | -207.403174 | 14.0 | -207.403876 | | |
| 7.6 | -207.403337 | 14.2 | -207.403882 | | |
| 7.8 | -207.403458 | 14.4 | -207.403887 | | |

Table S29: Total IH-FS-CCSD (2,0) DK3/ANO-RCC energies for the $1^3\Pi$ state of $LiMg^+$.

| R (Å) | E (a.u.) | R (Å) | E (a.u.) | R (Å) | E (a.u.) |
|---|---|---|---|---|---|
| 1.4 | -207.283387 | 8.0 | -207.465021 | 14.6 | -207.465335 |
| 1.6 | -207.357797 | 8.2 | -207.465033 | 14.8 | -207.465339 |
| 1.8 | -207.406068 | 8.4 | -207.465046 | 15.0 | -207.465343 |
| 2.0 | -207.437409 | 8.6 | -207.465059 | 15.2 | -207.465347 |
| 2.2 | -207.456920 | 8.8 | -207.465073 | 15.4 | -207.465351 |
| 2.4 | -207.468266 | 9.0 | -207.465087 | 15.6 | -207.465355 |
| 2.6 | -207.474221 | 9.2 | -207.465100 | 15.8 | -207.465359 |
| 2.8 | -207.476681 | 9.4 | -207.465114 | 16.0 | -207.465362 |
| 3.0 | -207.477202 | 9.6 | -207.465127 | 17.0 | -207.465377 |
| 3.2 | -207.476500 | 9.8 | -207.465140 | 18.0 | -207.465390 |
| 3.4 | -207.475204 | 10.0 | -207.465152 | 19.0 | -207.465400 |
| 3.6 | -207.473660 | 10.2 | -207.465164 | 20.0 | -207.465408 |
| 3.8 | -207.472157 | 10.4 | -207.465176 | 21.0 | -207.465416 |
| 4.0 | -207.470774 | 10.6 | -207.465187 | 22.0 | -207.465422 |
| 4.2 | -207.469562 | 10.8 | -207.465198 | 23.0 | -207.465427 |
| 4.4 | -207.468540 | 11.0 | -207.465208 | 24.0 | -207.465431 |
| 4.6 | -207.467705 | 11.2 | -207.465218 | 25.0 | -207.465435 |
| 4.8 | -207.467036 | 11.4 | -207.465228 | 26.0 | -207.465438 |
| 5.0 | -207.466509 | 11.6 | -207.465237 | 27.0 | -207.465441 |
| 5.2 | -207.466098 | 11.8 | -207.465246 | 28.0 | -207.465444 |
| 5.4 | -207.465783 | 12.0 | -207.465254 | 29.0 | -207.465446 |
| 5.6 | -207.465545 | 12.2 | -207.465262 | 30.0 | -207.465448 |
| 5.8 | -207.465368 | 12.4 | -207.465270 | 50.0 | -207.465462 |
| 6.0 | -207.465239 | 12.6 | -207.465277 | 100.0 | -207.465467 |
| 6.2 | -207.465147 | 12.8 | -207.465284 | 200.0 | -207.465467 |
| 6.4 | -207.465081 | 13.0 | -207.465291 | 300.0 | -207.465468 |
| 6.6 | -207.465040 | 13.2 | -207.465297 | | |
| 6.8 | -207.465015 | 13.4 | -207.465303 | | |
| 7.0 | -207.465002 | 13.6 | -207.465309 | | |
| 7.2 | -207.464996 | 13.8 | -207.465315 | | |
| 7.4 | -207.464997 | 14.0 | -207.465320 | | |
| 7.6 | -207.465002 | 14.2 | -207.465325 | | |
| 7.8 | -207.465011 | 14.4 | -207.465330 | | |

Table S30: Total IH-FS-CCSD (2,0) DK3/ANO-RCC energies for the $2^3\Pi$ state of $LiMg^+$.

| R (Å) | E (a.u.) | R (Å) | E (a.u.) | R (Å) | E (a.u.) |
|---|---|---|---|---|---|
| 1.4 | -207.087240 | 8.0 | -207.413751 | 14.6 | -207.414066 |
| 1.6 | -207.175876 | 8.2 | -207.413770 | 14.8 | -207.414070 |
| 1.8 | -207.241108 | 8.4 | -207.413786 | 15.0 | -207.414075 |
| 2.0 | -207.287256 | 8.6 | -207.413801 | 15.2 | -207.414079 |
| 2.2 | -207.319409 | 8.8 | -207.413815 | 15.4 | -207.414083 |
| 2.4 | -207.341647 | 9.0 | -207.413829 | 15.6 | -207.414088 |
| 2.6 | -207.357172 | 9.2 | -207.413841 | 15.8 | -207.414091 |
| 2.8 | -207.368244 | 9.4 | -207.413854 | 16.0 | -207.414095 |
| 3.0 | -207.376598 | 9.6 | -207.413865 | 17.0 | -207.414112 |
| 3.2 | -207.383084 | 9.8 | -207.413877 | 18.0 | -207.414126 |
| 3.4 | -207.388361 | 10.0 | -207.413889 | 19.0 | -207.414137 |
| 3.6 | -207.392773 | 10.2 | -207.413900 | 20.0 | -207.414147 |
| 3.8 | -207.396542 | 10.4 | -207.413910 | 21.0 | -207.414155 |
| 4.0 | -207.399772 | 10.6 | -207.413920 | 22.0 | -207.414162 |
| 4.2 | -207.402517 | 10.8 | -207.413931 | 23.0 | -207.414168 |
| 4.4 | -207.404821 | 11.0 | -207.413940 | 24.0 | -207.414173 |
| 4.6 | -207.406731 | 11.2 | -207.413950 | 25.0 | -207.414178 |
| 4.8 | -207.408291 | 11.4 | -207.413959 | 26.0 | -207.414182 |
| 5.0 | -207.409546 | 11.6 | -207.413968 | 27.0 | -207.414185 |
| 5.2 | -207.410543 | 11.8 | -207.413976 | 28.0 | -207.414188 |
| 5.4 | -207.411326 | 12.0 | -207.413984 | 29.0 | -207.414191 |
| 5.6 | -207.411933 | 12.2 | -207.413992 | 30.0 | -207.414193 |
| 5.8 | -207.412400 | 12.4 | -207.414000 | 50.0 | -207.414211 |
| 6.0 | -207.412755 | 12.6 | -207.414007 | 100.0 | -207.414216 |
| 6.2 | -207.413022 | 12.8 | -207.414014 | 200.0 | -207.414217 |
| 6.4 | -207.413223 | 13.0 | -207.414021 | 300.0 | -207.414217 |
| 6.6 | -207.413371 | 13.2 | -207.414027 | | |
| 6.8 | -207.413481 | 13.4 | -207.414033 | | |
| 7.0 | -207.413561 | 13.6 | -207.414039 | | |
| 7.2 | -207.413622 | 13.8 | -207.414045 | | |
| 7.4 | -207.413667 | 14.0 | -207.414050 | | |
| 7.6 | -207.413702 | 14.2 | -207.414056 | | |
| 7.8 | -207.413729 | 14.4 | -207.414061 | | |
